# Design Considerations for a Phase II Platform Trial in Major Depressive Disorder

**DOI:** 10.1002/pst.70025

**Published:** 2025-08-27

**Authors:** Michaela Maria Freitag, Dario Zocholl, Elias Laurin Meyer, Stefan M. Gold, Marta Bofill Roig, Heidi De Smedt, Martin Posch, Franz König, the EU‐PEARL MDD Investigators, Jelena Brasanac, Woo Ri Chae, Michaela Maria Freitag, Stefan Gold, Eugenia Kulakova, Christian Otte, Dario Zocholl, Marta Bofill‐Roig, Elias Laurin Meyer, Franz König, Martin Posch, Heidi de Smedt, Yanina Flossbach, Melissa Kose, Giulia Lombardi, Carmine Pariante, Luca Sforzini, Courtney Worrell, Tasneem Arsiwala, Alexandra Bobirca, Fernanda Baroso de Sousa, Pol Ibanez‐Jimenez, Gabriela Perez‐Fuentes, Toni Ramos‐Quiroga, Witte Hoogendijk, Francesco Benedetti, Fanni Laura Mäntylä

**Affiliations:** ^1^ Institute of Biometry and Clinical Epidemiology Charité—Universitätsmedizin Berlin, Corporate Member of Freie Universität Berlin, Humboldt‐Universität zu Berlin, and Berlin Institute of Health Berlin Germany; ^2^ Institute for Medical Biometry, Informatics and Epidemiology Faculty of Medicine, University of Bonn Bonn Germany; ^3^ Center for Medical Data Science, Medical University of Vienna Vienna Austria; ^4^ Berry Consultants Vienna Austria; ^5^ Department of Psychiatry and Psychotherapy Charité—Universitätsmedizin Berlin, Corporate Member of Freie Universität Berlin, Humboldt‐Universität zu Berlin, and Berlin Institute of Health Berlin Germany; ^6^ Medical Department, Section Psychosomatics Charité—Universitätsmedizin Berlin, Corporate Member of Freie Universität Berlin, Humboldt‐Universität zu Berlin, and Berlin Institute of Health Berlin Germany; ^7^ DZPG, German Center for Mental Health Berlin Germany; ^8^ INIMS, Universitätsklinikum Hamburg‐Eppendorf Hamburg Germany; ^9^ Johnson & Johnson Innovative Medicine Beerse Belgium; ^10^ Membership of The EU‐PEARL MDD Investigators is Provided in the Acknowledgments

**Keywords:** allocation, clinical trial simulations, futility stopping, major depressive disorder, multiple treatment arms, platform trial

## Abstract

Major depressive disorder (MDD) is one of the leading causes of disability globally. Despite its prevalence, approximately one‐third of patients do not benefit sufficiently from available treatments, and few new drugs have been developed recently. Consequently, more efficient methods are needed to evaluate a broader range of treatment options quickly. Platform trials offer a promising solution, as they allow for the assessment of multiple investigational treatments simultaneously by sharing control groups and by reducing both trial activation and patient recruitment times. The objective of this simulation study was to support the design and optimisation of a phase II superiority platform trial for MDD, considering the disease‐specific characteristics. In particular, we assessed the efficiency of platform trials compared to traditional two‐arm trials by investigating key design elements, including allocation and randomisation strategies, as well as per‐treatment arm sample sizes and interim futility analyses. Through extensive simulations, we refined these design components and evaluated their impact on trial performance. The results demonstrated that platform trials not only enhance efficiency but also achieve higher statistical power in evaluating individual treatments compared to conventional trials. The efficiency of platform trials is particularly prominent when interim futility analyses are performed to eliminate treatments that have either no or a negligible treatment effect early. Overall, this work provides valuable insights into the design of platform trials in the superiority setting and underscores their potential to accelerate therapy development in MDD and other therapeutic areas, providing a flexible and powerful alternative to traditional trial designs.

## 1 Introduction

Major depressive disorder (MDD) is a leading cause of disability worldwide, affecting an estimated 5%–6% of the population at any given time [1]. Additionally, MDD is associated with a twofold increased risk of developing other diseases and about 8–10 life years lost compared to the general population [2]. While several antidepressant treatments exist, a large proportion of patients experience only partial response (Partially Responsive Depression, PRD) or no response (Treatment‐resistant Depression, TRD) to at least two different treatments [3]. Up to one‐third of MDD patients do not achieve full symptomatic remission despite multiple medication attempts [4]. This highlights the urgent need for novel therapeutic options.

Unfortunately, approval rates of new psychiatric medications are low compared to other areas of medicine [5]. Innovative trial designs, such as platform trials, offer a potential solution to expedite drug development, particularly in early phases. Platform trials allow for the simultaneous evaluation of multiple interventions against a common control within a single protocol, enabling efficient screening of promising candidates. This approach can accelerate development timelines, reduce costs and potentially generate higher quality data compared to traditional trials while being more patient‐centric [6, 7]. Some successful adaptive platform trials have already been implemented in the past, e.g., REMAP‐CAP in Covid and lung diseases [8] and I‐SPY 2 in breast cancer [9, 10].

There are multiple definitions of platform trials [7, 11, 12]. This article considers them as clinical trials allowing for simultaneous and sequential evaluation of multiple interventions in one indication against a common control. They can possibly take into account specific disease sub‐types. Their unique feature is the possibility of treatments joining or leaving the trial over time [7, 13]. This definition is consistent with the one used in the Food and Drug Administration (FDA) guidance document on master protocols [14].

The Innovative Medicines Initiative (IMI) project EU‐PEARL (EU Patient‐Centric clinicAl tRial pLatforms) aimed to promote platform trials by establishing a framework for integrated research platforms across various disease areas, including MDD [15, 16]. This paper focuses on the design considerations for a platform trial specifically targeting TRD developed within the EU‐PEARL project.

We present a phase II platform trial design developed through collaboration with clinicians, statisticians, and other stakeholders. The trial aims to screen novel treatments and repurposed drugs for efficacy and safety in TRD patients.

Summaries of platform design considerations for one of the other use cases of EU‐PEARL, Non‐Alcoholic Steatohepatitis (NASH), have already been published [17, 18] alongside a general master protocol template [19].

During the design process, the aim is to maintain some of the flexibility that is available in separate trials, also within the platform trial. Still, the flexibility will be limited, as, e.g., endpoints should be the same in all arms. With these shared elements and a common framework the platform trial gains efficiency while introducing statistical challenges and design challenges. Adaptations, design and analysis elements have to be carefully tailored specifically to the area of application.

One critical consideration in platform trials is the definition of the control group for the analyses. Using all control data accumulated up to the point of the analysis offers higher statistical power, but may be susceptible to time trends, especially in long‐running trials. Alternatively, including only concurrent control data (i.e., only the data of control patients who could have been randomised to the treatment arm in question) can mitigate time trends but potentially reduce power. Recent methods incorporating time as a covariate can address these concerns [20, 21, 22, 23].

Another statistical challenge is the implementation of adaptive interim analyses, during which possible adaptations can be made. One such adaptation is the possibility of terminating treatment arms early and enabling faster decisions. In a platform trial, it is critical to drop non‐promising treatments due to futility and continue with the promising ones. By dropping one arm, resources become available to test another arm. This is especially valuable in phase II settings when screening for potentially active treatments is emphasised.

This paper investigates a broad range of design options for a phase II platform trial in TRD, including allocation strategies and dropping ineffective treatments. Section 2 details the hypotheses, analysis methods, design options and simulation setup. Section 3 presents the simulation results analysing the impact of these design choices on trial performance. We compare the proposed platform trial to traditional two‐arm trials, highlighting the potential benefits of the platform approach. Finally, Section 4 provides a discussion and concludes the paper.

## 2 Methods

### 2.1 General Design Aspects

**FIGURE 1:**
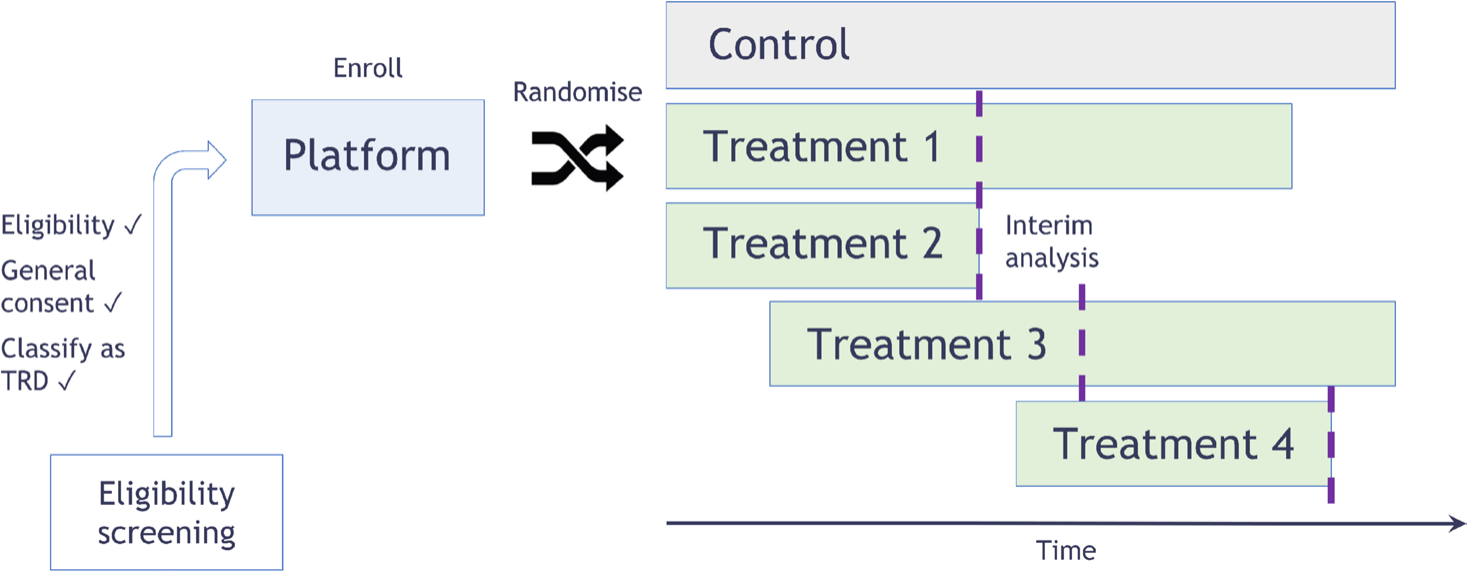
TRD platform trial design proposal. If patients are eligible for the platform, they are randomised to either control or one of the treatment arms within the platform. Different drugs can enter the trial at different time points. The design might also allow for interim analyses to drop treatment arms early. The time point of the interim analyses is indicated by a dotted line.

The target population for the platform trial are patients with TRD. If patients are eligible for the trial, they are invited to enrol and subsequently be randomised to either the placebo control arm or a treatment arm. The substances will be added to current treatment with antidepressants of one of two classes (selective serotonin reuptake inhibitors SSRIs or serotonin norepinephrine reuptake inhibitors SNRIs). Add‐on of a placebo to current SSRI or SNRI treatment is compatible with clinical practice and was deemed the appropriate comparison and ethically acceptable based on the consultations the EU‐PEARL consortium had with key stakeholders, including regulatory agencies, ethics committee members and patient/lived experience representatives. As the primary outcome measure, we evaluate the change in Montgomery‐Åsberg Depression Rating Scale (MADRS) score between week 6 and the baseline value. Both the duration of 6 weeks for short‐term trials and the use of MADRS score are common in depression trials [24]. The efficacy of each experimental treatment is compared against a shared control in a superiority setting, i.e., when the objective is to show that the respective experimental arm is better than the control arm. The arms are tested individually without comparing the effect of one experimental treatment to that of another. The platform trial also does not investigate a global hypothesis. For the individual analyses in the platform trial, we use a one‐sided significance level of 0.05 and only concurrent controls, meaning only information from patients in the control arm that could have been randomised to the treatment arm in question. Figure 1 shows a schematic of the specific platform trial design for TRD patients. Furthermore, the allocation ratio to control is dependent on the actual number of enrolling treatment arms. We suggest a modified block randomisation, which is discussed in detail in Section 2.2.3. For the specific design elements of the platform trial, we mainly focus on the selection of adequate allocation ratios, futility boundaries and targeted sample sizes per experimental arm compared to placebo. We will also consider standard two‐arm trials and compare their performance later on in the Results Section to the performance of platform trials. For better comparability, we selected the same general design aspects for both types of trials.

### 2.2 Statistical Methods

Consider a platform trial where investigational treatments j, $j\in \left\{1,\ldots ,J\right\}$, are compared to a common control c. The objective of this platform trial is to find any efficacious treatment, i.e., any treatment that lowers the MADRS score at week 6 compared to a control treatment. The corresponding baseline value of the MADRS score will be measured at the time point when patients are being randomised into either treatment arm j or control arm c. For comparing arm j with the control arm, only control data that is concurrently collected shall be used, thereby defining a different set $c_{j}$ of control data for every treatment j.

#### 2.2.1 Statistical Model and Hypothesis

For the analysis of the primary endpoint, i.e., the change in MADRS score between week 6 and baseline controlled for the baseline value, an analysis of covariance (ANCOVA) is conducted using the factor treatment and adjusting for baseline MADRS score and time period as covariates. We define the categorical covariate time period as time intervals that change whenever a treatment arm enters or leaves the platform trial. This means that the change of time periods is dependent on the actual platform trial and the results of the interim analyses. This model highlights the influence of a patient's expectation to receive a placebo on the placebo response.

ANCOVA assumes linearity of the covariate effect and the absence of covariate‐by‐group interaction. The idea of the ANCOVA is to use regression to control (i.e., adjust) for additional covariates such as the baseline value so one can study the post‐treatment measure free of the proportion of variance linearly associated with the baseline. The ANCOVA is the preferred analysis method for randomised clinical trials with a pre‐ and post‐treatment measurement in case of continuous endpoints and homogeneous covariance matrices [25, 26, 27]. In the context of a platform trial, it is reasonable to adjust not only for the MADRS score at baseline but also for the factor time period to avoid bias in the estimates due to time trends and to address the change in allocation ratios due to entering or dropping of arms. Including the factor time period is also suggested by the FDA guidance on master protocols [28] to ensure that the type I error rate is not negatively affected when allocation ratios and control responses change over time. In the Supporting Information, we explore the advantages of including the covariate time period by comparing the model to one adjusting only for baseline. We refer to Bofill et al. [20] for a formal definition of time periods in the context of platform trials without interim analyses and for more detailed methods on how to adjust for potential time trends.

As mentioned before, we test the different null hypotheses $H_{j}^{0}$ separately for every treatment j, $j\in \left\{1,\ldots ,J\right\}$. We therefore fit different models for every treatment arm j using the data of treatment arm j and the corresponding concurrent control data $c_{j}$ only. The formal models $M^{j}$, $j\in \left\{1,\ldots ,J\right\}$ can be written as

(1) $$\begin{array}{c} M^{j}:Y_{i,h_{j}}=\alpha_{j}+\beta_{j}G_{i,h_{j}}+\gamma_{j}X_{i,h_{j}}+\sum_{l=t_{j,\text{start}}+1}^{t_{j,\text{exit}}}\delta_{l}1_{\left\{P_{i,h_{j}},=,l\right\}}+e_{i,h_{j}} \end{array}$$

whereby $Y_{i,h_{j}}$ is the change from baseline to 6‐week MADRS score of person i in treatment group $h_{j}$, and $X_{i,h_{j}}$ the corresponding baseline value. In every model $M_{j}$, the index $h_{j}$ only takes two different values, j for the treatment arm in question and $c_{j}$ for the concurrent control group. Thus $Y_{i,j},i=1,\ldots ,n_{j}$ and $Y_{i,c_{j}},i=1,\ldots ,n_{c_{j}}$ denote the observations in treatment group j and the corresponding concurrent control. $G_{i,h_{j}}$ is the treatment indicator for patient i in group $h_{j}$ (i.e., $G_{i,c_{j}}=0$ if the patient is in the control group and $G_{i,j}=1$ if he or she is in the treatment group), $\beta_{j}$ is the treatment effect and $\gamma_{j}$ the coefficient for the baseline value. $P_{i,h_{j}}$ is the period where the i−th patient in group $h_{j}$ is recruited (please note that we index the patients in each group separately), and $t_{j,\text{start}}$ and $t_{j,\text{exit}}$ are the first, respectively, last time periods where treatment arm j is in the platform trial. Therefore, $\alpha_{j}$ is the response in the control arm in the period where arm j enters the platform trial. If there is no time trend, then $\delta_{l}=0$ for all periods l where treatment j is part of the platform trial and $\alpha_{j}$ can be interpreted as the overall response in the control arm. The variable $e_{i,h_{j}}$ denotes the residual term, which is assumed to be normally distributed with zero mean and constant variance $\sigma^{2}$.

Practical use of ANCOVA requires estimation of $\gamma_{j}$, which is a function of the within‐group variances and correlation of the pre‐treatment and post‐treatment scores. For the fitting of the models $M^{j}$, only the data of the $n_{j}$ subjects randomised to the experimental treatment arm j of interest and the corresponding concurrent control data of $n_{c_{j}}$ subjects in the control group is used. Please note that the size $n_{c_{j}}$ of concurrent control data might differ distinctly between experimental treatments j because the number of concurrently running treatment arms and therefore the allocation ratio differs. More details on the size of individual control groups can be found in the Supporting Information.

Based on the models $M^{j}$, elementary null hypotheses $H_{j}^{0}$ are tested at a one‐sided significance level α to demonstrate the efficacy of an experimental treatment j against the control group $c_{j}$, i.e.,

$$H_{j}^{0}:\beta_{j}\leq 0\;\mathrm{vs}\;H_{j}^{A}:\beta_{j}>0$$

#### 2.2.2 Effect Size Definition

The standardised effect sizes are based on the model defined in (1). They follow a logic similar to the calculation of Cohen's d, see Equation 2. In the presented version, we assume that there is no time trend and by using σ as the standard deviation of the change in MADRS score, we adjust for the correlation between the baseline value and the value at week 6 as well as for the covariate baseline value. Assuming $e_{i,h_{j}}∼N\left(\mu ,\sigma^{2}\right)$ implies equal variances for both treatment and control arms.

(2) $$d=\frac{\beta_{j}}{\sigma }$$

The value used in the simulations for the standard deviations is based on a variance–covariance matrix of a placebo control arm in a past phase II study in augmentation treatments in MDD. Based on the same data, it is assumed that the MADRS score reduces from 32 points at baseline to 20 at week 6 in the control group and that the correlation between baseline value and week 6 is about 0.2. According to Equation (2), a value of d=0.2 corresponds to an absolute reduction of 2.3 in the MADRS score compared to the control group, d=0.35 corresponds to an absolute reduction of about 4, and d=0.5 of about 5.7.

We assume a standardised effect size d=0.35 to be the clinically relevant effect for augmentation strategies in TRD. An effect of 0.5 is regarded as large.

#### 2.2.3 Allocation Ratios and Randomisation Methods

There are many different options to define allocation rates to different treatment arms and the control arm in multi‐arm trials like a platform trial. Common ones are a 1:1:…:1 allocation where every treatment arm and the control arm are allocated the same proportion of patients, and a 1:1:..:1:x allocation where x relates to the ratio in the control arm. All treatment arms get the same number of patients, but the control arm is allocated a different fraction. The value x can either be constant or dependent on the number k of treatment arms concurrently enrolling in the trial. For multi‐arm trials a square‐root allocation, i.e., 1:1:…:1:$\sqrt{k}$, yields good results [29]. It minimises the standard error of treatment effect estimates for normally distributed endpoints with equal variances across groups. However, adding and terminating treatment arms during the course of a platform trial impacts the performance of allocation rates. In these scenarios, other rules than the one for traditional multi‐arm trials may offer the best results [30].

It should be noted that the (placebo) response in patients with MDD strongly depends on factors like the expectancy to receive a placebo, meaning it depends on the number of treatment arms that are recruiting at the same time [31]. In order to avoid large variations in the treatment effect over time, the allocation probability to the control arm needs to be carefully regulated. Therefore, it is reasonable to consider a Minimum Allocation Probability to Control (MAPC). Throughout the project, this MAPC was discussed multiple times with clinical experts. Finally, it was recommended that at least about one‐third of patients should be randomised to the control arm. For the simulations presented in the Results Section, we used a MAPC of 35%. Other values were also investigated and are presented in the Supporting Information.

All considered allocation ratios can be easily achieved by using simple randomisation for any x by modifying the randomisation probabilities accordingly. In order to limit the variability introduced by simple randomisation, we implemented a modified version of block randomisation. We were aiming to get blocks of minimal size to reach the targeted allocation ratio of 1:1:…:1:x, where x corresponds to the weight for the control arm. For the 1:1:…:1 allocation, basic permuted blocks are created with the number of open arms (including control) as the length of the block to be permuted, i.e., number of currently enrolling treatment arms (=k) + 1. For example, aiming at an allocation ratio of 1:1:1:1, a block with the spots $T_{1}T_{2}T_{3}C$ is permuted. In the other allocation methods aiming at a 1:1:..:1:x ratio, there is more than one control spot in each block. If x is an integer, the final block length is simply k+x, with x spots for the control in a permuted block. As an example, for an allocation ratio of 1:1:1:3, a block with the spots $T_{1}T_{2}T_{3}\mathrm{CCC}$ is permuted. If x is not an integer, we combine block randomisation with some random elements to determine how many spots should eventually be added. The basic block has k spots for experimental treatments and y spots for the control arm, with y=⌊x⌋. Furthermore, for each block, it is decided whether to add an additional spot for the control arm with a probability of $\text{Frac}\left(x\right)$ or not. $\text{Frac}\left(\right)$ returns the fraction to the right of the decimal point of x, where x is a real number. For example, for 1:1:1:$\sqrt{3}$ allocation with $x=\sqrt{3}=1.73$, this method results in a minimal block length of 4. With a probability of 1−0.73=0.27, such a permuted block $T_{1}T_{2}T_{3}C$ of length 4 is selected, while with probability 0.73, a block $T_{1}T_{2}T_{3}\mathrm{CC}$ of length 5 is chosen, adding an additional spot for control. By applying this principle to randomly add an additional control spot, the targeted allocation ratio is approximately reached while implementing a minimal block length in the randomisation procedure. This modified procedure combines elements from traditional block randomisation and random elements like in simple randomisation. With the blinding of the study and the random element in allocation, the study is not easily decodable. However, for the practical implementation in a study, one should consider a randomisation list that is even harder to decode, e.g., by larger and variable block sizes. For the simulations, we have therefore implemented the described randomisation with double the block size.

### 2.3 Simulations

During the design stage and simulation of any randomised controlled trial, several design specifications and assumptions are needed to understand the behaviour and operating characteristics of the trial. Design choices are aspects that can be controlled by the decision maker, and assumptions refer to unknown quantities that have to be estimated from preexisting data [32, 33]. For a trial with continuous pre‐treatment and post‐treatment values analysed by an ANCOVA, the sample size per group, the expected (or clinically relevant) effect, the assumed correlation between pre‐treatment and post‐treatment values, and the significance level are needed to calculate the power. In this simple case, the sample size and significance level are design choices, and the rest are assumptions about the true nature of the treatment effect, while the operating characteristic of interest is the power. For designs with adaptive elements, such as group sequential and platform trial designs, typically, many more design choices and assumptions need to be made while at the same time evaluating additional operating characteristics. As an example, in the case of a group sequential design with one interim analysis and the option to stop for futility, additionally, the time point of the interim analysis and the futility stopping boundary are needed as design parameters and another operating characteristic to evaluate is the probability to stop at interim. Due to their flexibility with respect to incorporating adaptive design features, platform trials require even more design choices and assumptions for the simulation setup, and many more operating characteristics are evaluated.

In this subsection, we will first give a description of the general simulation settings with the design choices and assumptions considered. Then we specify operating characteristics of interest. R version 4.2.1 was used for the simulation, and the code is publicly available on Github [34].

The main objective of the simulations was to investigate whether a platform trial offers more efficiency in terms of sample size or runtime compared to separate two‐arm trials in the context of phase II MDD trials. A corresponding sequence of two‐arm trials will therefore act as a reference for the platform trial.

#### 2.3.1 Simulated Trial Designs

For the simulations, we consider a base design with some fixed parameters and some that vary over a range of options. All parameters that are varied are described in Table 1. For each combination of simulation parameters, 10 000 simulation runs were performed. Ten thousand replicates correspond to a Monte Carlo standard error for a rate of 0.05 (=significance level) of $\sqrt{0.05\cdot \left(1-0.05\right)⁄10000}\approx 0.002$ and the worst case simulation error for rates of $\sqrt{\left(0.5\cdot 0.5\right)⁄10000}=0.005$.

**TABLE 1:** Parameters required to be specified for the simulation study. They are classified as either design choices or assumptions made regarding the platform trajectory or treatment effects. Some values are fixed, and some vary for different simulation scenarios.

Name	Type	Investigated values	Description
Randomisation	Design choice	(Modified) Block	A modified block randomisation was implemented, which combines traditional block randomisation with random adding of controls to allow for minimal block length (details see Section 2.2.3)
Allocation ratio	Design choice	1:…:1 1:…:1:k 1:…:1:$\sqrt{k}$ 1:…:1:$\sqrt{k}$ with MAPC*	Patients are randomised to one of the treatment arms or to the control arm with the given ratio. The variable k denotes the number of concurrently enrolling treatment arms. * Different possible values for the MAPC are investigated in the Supporting Information. Here, the results of the selected value 35% are shown.
Analysis method	Design choice	One covariate* Two covariates	The analysis is based on an ANCOVA, which either adjusts only for the covariate baseline value or additionally for the time period.
Timing of the interim analysis	Design choice	No interim analysis, 50%	Timing of the interim analysis of a single treatment arm as a proportion of the planned sample size for this treatment arm (counting observed outcomes).
Futility stopping rule	Design choice	No futility stopping, 0.2, 0.25, 0.3, 0.35, 0.4, 0.45, 0.5	Treatment arms will be stopped for futility at the time point of the interim analysis if the one‐sided interim *p*‐value is above this threshold.
Sample size per treatment arm	Design choice	Variable options from 40 to 120	Number of patients after which the final analysis of one treatment arm is conducted.
Initial treatments	Assumption	3, 6	Number of treatments available at the beginning of the platform trial
Timing of new treatments	Assumption	20%, 100%	Every month, a new treatment can enter the platform with a given probability if the maximal number of concurrently enrolling treatment arms is not yet reached.
Standardised effect size	Assumption	0, 0.2, 0.35, 0.5	Every treatment that enters the platform trial is randomly assigned one of these standardised effect sizes d with predefined probability $\theta_{d}$, fulfilling $\theta_{0}+\theta_{0.2}+\theta_{0.35}+\theta_{0.5}=1$. The effect sizes are expressed in standardised mean differences between baseline and 6‐week MADRS scores. A variance–covariance matrix based on data from past studies is used for standardisation.
Effect size distribution	Assumption	Equal, pessimistic	Different scenarios for the probabilities of the effect sizes are investigated. The equal scenario sets $\theta_{0}=\theta_{0.2}=\theta_{0.35}=\theta_{0.5}=0.25$ and the pessimistic scenario $\theta_{0}=0.5$,$\theta_{0.2}=0.3$, $\theta_{0.35}=0.1$, and $\theta_{0.5}=0.1$.
Time trend	Assumption	No time trend, as step function*	In the Supporting Information, the possible drift over time is modelled by a step function with steps at the beginning of every time period. The time trend model is exemplary and only serves the purpose of illustration.

* Values are only presented in the Supporting Information.

As the base design, we used a platform trial with a common control group and a maximum of six concurrently running treatment arms. Only concurrent controls are used for the analyses. Every week, a mean number of seven patients is recruited, with seven being most likely (90%) and a slight variability to six or eight patients per week (both with probability 5%). Assumptions about the recruitment speed were made based on a site‐readiness survey of candidate clinical sites by the EU‐PEARL consortium. The expected site network for this trial will comprise 30 centres. Their number of annual patient contacts, likelihood of meeting eligibility criteria, and enrollment probability based on the sites' documented experience in previous trials of pharmacological treatments for MDD were taken into account.

Every week, an analysis can take place if the primary outcome has been observed for the required number of patients in a treatment arm. The targeted sample size per treatment arm j is $n_{j}=80$ patients for the final analysis with an optional interim analysis after 50%, if not stated otherwise. This sample size is based on considerations for a two‐arm trial with the same effect size assumptions and unequal sample sizes between the treatment arm and control arm. Assuming always running at least three experimental treatment arms (not counting control) in the platform and using a ‘$\sqrt{k}$ allocation’, we get an equivalent two‐arm trial with a ratio of at least 1:1.73. A sample size of $n_{j}=80$ then leads to 80% power for the clinically relevant effect size of 0.35 and a one‐sided significance level of α=0.05. If a 1:1 randomisation was used, a sample size of $n_{j}=80$ would result in a power of about 72%.

In the simulations, whenever a new treatment arm enters, we randomly draw the effect size of this treatment. The four standardised effect sizes d=0, d=0.2, d=0.35, and d=0.5 are assumed as either equally likely, meaning the probability of each effect size is $\theta_{0}=\theta_{0.2}=\theta_{0.35}=\theta_{0.5}=0.25$, or more pessimistically distributed, meaning $\theta_{0}=0.5$, $\theta_{0.2}=0.3$, $\theta_{0.35}=0.1$, and $\theta_{0.5}=0.1$. We assume that the outcome is observed immediately and analyses (and possible trial adaptations) are conducted as soon as a target number of outcomes has been observed. The decisions are based on *p*‐values using an ANCOVA adjusting for the baseline value and time period. A nominal one‐sided significance level α=0.05 was applied for the final analyses.

The platform starts with three arms. At the beginning of each month, a new arm enters the trial with 20% probability if the maximal number of six concurrently enrolling treatments (not counting control) in the platform has not yet been reached. For simulation purposes, we set 1 month to 4 weeks. Regarding the availability of new experimental arms, approximately 2–3 new arms per year seemed reasonable for the consortium. Especially since the trial's design allows for a wide range of research questions that consider not only new drugs in a phase II setting but can also accommodate many repurposed drugs or drugs already licensed for MDD. This includes comparative effectiveness studies of drugs or combinations and augmentation strategies that are already established in clinical practice but lack head‐to‐head comparisons. Thus, the trial is not restricted to only the industry drug development pipeline but can also be used for these additional research questions, which could be supported by public or philanthropic funders. Therefore, the suggested use of an entry probability for new arms of 20% each month, if a slot becomes available, was deemed realistic by the consortium. We also investigate a scenario with a more optimistic assumption that there are always treatments available to be entered into the platform trial. So, at the beginning of each month, if a treatment arm has been removed from the platform, another treatment arm will replace it. This shows the maximum benefit of a platform trial compared to multiple two‐arm trials and will act as a best‐case benchmark scenario. In the Comparison Section, results for both scenarios are shown. More results for the maximal setting are provided in the Supporting Information.

After 60 months, the platform trial will fade out. This means it ends once all treatment arms have completed recruitment or stopped. In order to achieve a more homogeneous total sample size in the investigated platform trial designs, we only allow treatments to enter if at least one‐fifth of the targeted per‐arm sample size is expected to be recruited until month 60.

Starting with this base design, we investigate different design elements separately. We first evaluate the impact of different allocation ratios to control and select the most promising one, which will be fixed for the following simulations. Secondly, we investigate the impact of an interim analysis after the primary endpoint is observed for half of the targeted patients per treatment arm. We only consider early stopping of a treatment arm for futility, which is triggered if the *p*‐value is larger than a pre‐defined futility boundary. Different values for this futility boundary are investigated. In our design, we do not account for futility stopping in the significance level of the final analysis. Therefore, the futility boundaries can be considered non‐binding. This offers more flexibility because it allows in principle overruling the futility stopping decision at interim while still preserving the type I error rate. However, in order to present operating characteristics, predictability is needed. So, in the visualisation of the simulation results, we treat the futility boundaries as binding, i.e., drop an arm if the futility threshold is reached at interim. As a third step, we analyse the impact of the targeted sample size per treatment arm on the operating characteristics of the platform trial. Additionally, in order to be able to compare the results of the platform trial to a more traditional approach, we simulate standard parallel group designs with 1:1 allocation between treatment and control, both with and without an interim analysis after the primary endpoint is observed for half of the targeted per‐arm sample size. For comparability, we always use the same general assumptions (e.g., effect size and per‐arm sample size) as for the platform trial design.

#### 2.3.2 Operating Characteristics

Standard operating characteristics reported for traditional parallel group designs are power, type I error per investigated treatment, as well as the expected sample size. In the context of platform trials, many more operating characteristics can be of interest depending on the objective of the platform trial. Table 2 gives an overview of operating characteristics we considered important for the platform trial in MDD. Some of them are only presented in the Supporting Information. As several treatments are investigated within a platform, operating characteristics can be defined on several levels, e.g., on the platform level, by treatment arms or conditional on a certain effect size. For example, one could be interested in the expected sample sizes both on the treatment and platform levels for budgeting reasons. Different power definitions have been suggested in the context of multiple testing and multi‐armed clinical trials [35], e.g., conjunctive (‘reject all false null hypotheses’) or disjunctive power (‘reject at least one false null hypothesis’) [36]. The goal of this platform trial is to identify any treatment that could have a beneficial impact on the health of patients. Therefore, we are interested in individual power conditional on the true effect size. We are also focusing on pairwise errors and do not investigate multiple testing or family‐wise error rates as this is not necessarily required for relevantly distinct treatments in exploratory settings [37, 38].

**TABLE 2:** Operating characteristics of interest in platform trials. This table gives names and descriptions of different operating characteristics that will be evaluated in the Simulation Section. It also states on which levels the characteristics are calculated, i.e., if they are characteristics of the overall platform, of arms in the platform or stratified by different effect sizes.

Name	Level	Description
Concurrently investigated treatment arms	Platform	Gives the mean number of investigational treatments (not counting control) that are concurrently evaluated in the platform at any time. The time point when new treatment arms enter the platform and the duration of their stay are dependent on different random factors.
Duration	Treatment*	Gives the number of weeks it takes for a treatment to finish within the platform trial.
Rate of decisions made for treatment arms	Per effect size	The rate of the different types of decisions among all treatment arms with a specific effect size is evaluated. Decisions per treatment arm can be ‘success’, ‘failure at final analysis’ or ‘stopped for futility’. If the effect size is d=0 the success rate corresponds to the type I error rate. If the effect size is greater than 0, the success rate corresponds to the power and the rates for failure and stopped for futility together give the type II error rate.
Sample size	Platform*, treatment*	Gives the average number of patients in the whole platform trial and the individual treatments, taking into account potential interim analysis.
Size of control group for interim analyses and final analyses	Platform*, treatment*	Only concurrent controls are used for the interim and final analyses of a specific treatment. Because the allocation to control is not fixed, this value can vary notably.
Standardised expected number of treatment arms	Platform	Gives the mean number of different experimental treatments that can be investigated per 1000 patients in the entire platform trial.

* Values are only presented in the Supporting Information.

## 3 Simulation Results

We will first present the results of the impact of different allocation ratios to control. Secondly, interim stopping rules are investigated, followed by an analysis of the targeted sample size per treatment arm. Please note that the efficacy boundaries have not been adjusted to account for the futility stopping rule. This approach is sometimes referred to as ‘non‐binding futility stopping’. However, when evaluating operating characteristics such as the actual Type I error rate and power, we fully adhere to the pre‐specified futility rule as if it was binding. Specifically, an arm is discontinued whenever the futility boundary is met. Consequently, our calculations of pairwise/marginal power incorporate the impact of the futility rule, ensuring that the empirical power reflects its influence on trial outcomes. Using non‐binding futility boundaries, i.e., not adjusting the efficacy boundaries, but considering them as binding when evaluating the operating characteristics, is in line with regulatory guidance and FDA experience [39, 40]. Finally, platform trials for the selected allocation strategy and a possible interim analysis for futility are compared to a sequence of standalone two‐arm trials.

At the beginning of every Subsection, we highlight parameters that differ from the ones in previous Subsections. All others stay the same. In this main paper, we only present a limited number of operating characteristics and simulation scenarios. We refer to the Supporting Information for more details.

### 3.1 Comparison of Allocation Ratios

**FIGURE 2:**
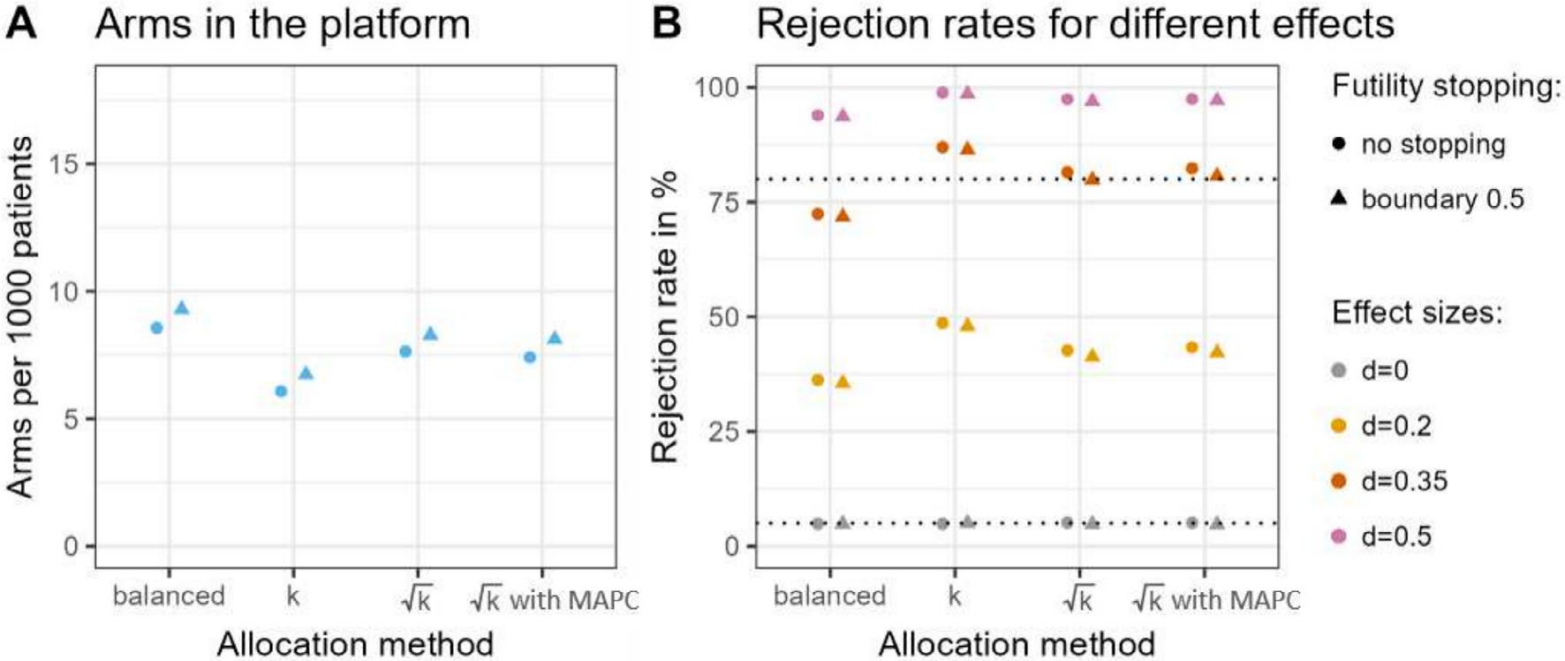
Experimental treatment arms per 1000 patients (without control arm) during the runtime of the platform trial, and rejection rates for platforms running at expected capacity, meaning three treatment arms at the beginning of the platform trial and a probability of 20% per month for a new treatment arm to enter. The targeted sample size per experimental treatment arm was fixed at $n_{j}=80$, and all effect sizes were assumed to be equally likely, i.e., $\theta_{0}=\theta_{0.2}=\theta_{0.35}=\theta_{0.5}=0.25$. The circles give the values without the implementation of an interim analysis, and the triangles the corresponding values when a futility boundary of 0.5 for the *p*‐value is applied. The MAPC for the ‘$\sqrt{k}$ allocation’ was set to 35%. (A) depicts the mean number of arms that can be evaluated per 1000 patients in a corresponding platform trial. In (B), the percentage of rejected null hypotheses is depicted stratified by the four different investigated effect sizes. It equals the type I error rate for d=0 and the power for the other values of d. The type I error rate is always controlled at 5%. This value is indicated by the lower dotted line. The higher dotted line highlights the 80% mark.

We simulated platform trials with different choices of allocation methods. The targeted sample size per treatment arm was set to $n_{j}=80$, and we considered both a version with no interim analysis and a version with an interim analysis. The interim analysis may take place after the primary endpoint has been observed for 50% of the targeted sample size, and a futility boundary of 0.5 for the *p*‐value was incorporated. The allocation methods considered are 1:1:…:1 randomisation (called ‘balanced allocation’), 1:1:…:k randomisation (called ‘k allocation’), 1:1:…:$\sqrt{k}$ (called ‘$\sqrt{k}$ allocation’) and 1:1:…:$\sqrt{k}$ with a MAPC of 35%. For a maximum of 6 concurrently running treatment arms (not counting control), the ranges for allocation to control are 14.3%–50% for the ‘balanced allocation’, 29%–50% for the ‘$\sqrt{k}$ allocation’, and 35%–50% for the ‘$\sqrt{k}$ allocation’ with a MAPC. In the ‘k allocation’, always 50% of the patients are randomised to the control arm. The mean number of experimental treatment arms per 1000 patients in the platform trial is the highest for the ‘balanced allocation’ with 8.6 arms in case no interim analysis takes place and 9.3 arms when an interim analysis with a futility boundary of 0.5 is implemented. When applying the ‘k allocation’, substantially fewer arms (6.1 without futility and 6.7 with futility) can be investigated due to the higher proportion of controls. The ‘$\sqrt{k}$ allocation’ and ‘$\sqrt{k}$ allocation’ with a minimum allocation to control of 35% are somewhere in between with a mean of 7.6 arms per platform for the version without a futility analysis and 8.3 with a futility analysis when we do not apply a MAPC, and 7.4 and 8.1, respectively, when we apply a MAPC, as seen in Figure 2A. The average number of concurrently running experimental treatment arms (without control) follows the same relation between the methods. The ‘balanced allocation’ has the lowest average number of concurrently running experimental treatment arms (2.2 with futility and 2.4 without), the ‘k allocation’ has the highest (3.8 with futility and 3.9 without) and the ‘$\sqrt{k}$ allocation’ methods are in between (2.9 with futility and 3.1 without for the version without a MAPC, and 3.1 and 3.3 for the version with one of 35%). Directly correlated to the number of controls per comparison, the duration of experimental treatment arms is the shortest for the ‘balanced allocation’ and the longest for the ‘k allocation’. The arms in the scenario with ‘$\sqrt{k}$ allocation’ take more time than the ones in the ‘balanced allocation’ scenario, and when using a MAPC of 35% they again take a little longer, see Supporting Information.

Figure 2B shows the percentage of rejected null hypotheses in platform trials using the different allocation methods stratified by the four standardised effect sizes d=0, d=0.2, d=0.35, and d=0.5. All effect sizes are assumed to be equally likely, i.e., $\theta_{0}=\theta_{0.2}=\theta_{0.35}=\theta_{0.5}=0.25$. For d=0, the rejection rate equals the type I error rate, which is always controlled at a one‐sided level of 5%. In the other scenarios, the power is always higher the number of control comparators per decision. So the ‘k allocation’ yields the highest power, followed by ‘$\sqrt{k}$ allocation’ with a MAPC and ‘$\sqrt{k}$ allocation’ without one. The lowest power is achieved using ‘balanced allocation’. With only 72% it even yields under 80% power in the case of d=0.35 (the clinically relevant effect size) where all other methods have over 80% power.

Overall, even though the ‘k allocation’ method yields the highest power, it also has the lowest ratio of patients on treatment, by far the highest duration per experimental treatment arm, and the lowest (standardised) number of arms that can be investigated in the platform. The ‘balanced allocation’ enables investigation of the most treatment arms per 1000 patients and the lowest ratio of patients on control but the power is much smaller than for the other allocation methods. As a trade‐off between number of arms investigated and power, we selected the ‘$\sqrt{k}$ allocation’ method with a MAPC of 35% for control. It achieves the second highest power of all the allocation methods considered and enables the investigation of more experimental treatment arms within the platform compared to the ‘balanced allocation’ method. Furthermore, this MAPC was chosen based on clinical considerations: In patients with MDD, the placebo response may vary due to expectancy effects of whether placebo is given or an experimental drug. Randomising at least one‐third to the control arm may help to mitigate this risk. A more thorough investigation of the impact of different values for the MAPC can be found in the Supporting Information.

### 3.2 Comparison of Futility Stopping Rules

**FIGURE 3:**
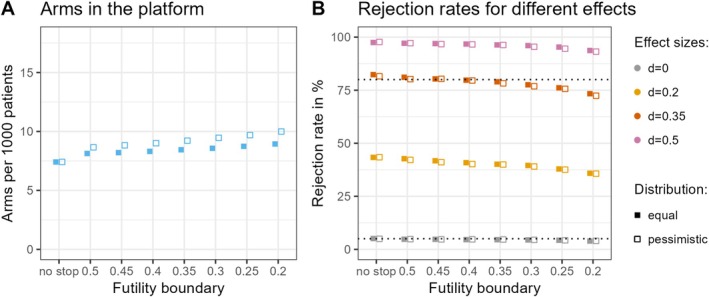
Experimental treatment arms per 1000 patients (without control arm) during the runtime of the platform trial, and rejection rates when implementing different futility rules. On the *x*‐axis the futility boundary on the *p*‐value scale is shown, i.e., we stop for futility if the corresponding *p*‐value is larger than the futility boundary. No stop means that no futility analysis is performed. In the scenario with the filled squares all effect sizes are assumed to be equally likely, i.e., $\theta_{0}=\theta_{0.2}=\theta_{0.35}=\theta_{0.5}=0.25$. With the empty squares we present results for a more pessimistic scenario with probabilities of assignment $\theta_{0}=0.5$, $\theta_{0.2}=0.3$, $\theta_{0.35}=0.1$, and $\theta_{0.5}=0.1$. (A) gives the mean number of experimental treatment arms per 1000 patients in the platform trial. All experimental treatment arms are included in this number regardless of the underlying effect size. In (B), the percentage of rejected null hypotheses is depicted stratified by the four different investigated effect sizes. It equals the type I error rate for d=0 and the power for the other values of d. The type I error rate is always controlled at 5%. This value is indicated by the lower dotted line. The higher dotted line highlights the 80% mark.

We simulated platform trials with different choices of futility boundaries. We applied a ‘$\sqrt{k}$ allocation’ with a MAPC of 35% and a targeted sample size of 80 patients per experimental treatment arm. The interim analysis was carried out after the primary endpoint was observed for 40 patients in an experimental treatment arm. We present two different scenarios for the assignment probabilities of the four considered standardised effect sizes: an equal scenario and a more pessimistic one. In the equal scenario, all effect sizes are assumed to be equally likely, i.e., $\theta_{0}=\theta_{0.2}=\theta_{0.35}=\theta_{0.5}=0.25$. For the more pessimistic scenario, these probabilities of assignment are $\theta_{0}=0.5$, $\theta_{0.2}=0.3$, $\theta_{0.35}=0.1$, and $\theta_{0.5}=0.1$. In the Supporting Information, we additionally report the observed probabilities for stopping for futility depending on the effect sizes. Overall, the rejection rates seen in Figure 3B are quite similar in both scenarios. With a stricter stopping rule, the duration of the platform decreases, as does the number of control comparators per decision. As a trade‐off for using non‐binding futility boundaries, the power decreases with stricter boundaries but also more arms can be tested. The number of experimental treatment arms that can be tested per 1000 patients in the platform differs notably between the scenarios, see Figure 3A. In the more pessimistic scenario, more experimental treatment arms can be investigated than in the equal scenario. This difference becomes more prominent the stricter the applied futility boundary for the *p*‐value. It goes as high as a difference of 1.06 arms per 1000 patients for a futility boundary of 0.2. Here we only present the overall number of arms. The mean number of concurrently running experimental treatment arms (without control) is 3.3 for both scenarios in case of no interim analysis. When a futility boundary is implemented, the number goes from 2.8 to 3.1 in the equal scenario and from 2.5 to 2.9 in the pessimistic scenario with the smaller numbers corresponding to the strictest futility boundaries. The exact values are presented in the Supporting Information.

Figure 3 shows that in both scenarios the difference between not stopping at all and a very soft boundary of 0.5 is relatively large for the standardised number of experimental treatment arms that can be evaluated in the platform. The impact on the power, however, is very small. The differences in these operating characteristics between the individual steps for the futility boundary are not very pronounced when comparing one step with the next. Nevertheless, the differences become clearly perceptible when comparing more distant values, such as the boundaries 0.5 and 0.25. Here, not only does the number of arms that can be examined increase, but the power also decreases notably. Thus, one has to decide for the specific use case, how aggressive the applied stopping rules should be. As this is a phase II study, we recommend applying futility stopping with a boundary of at least 0.5. It is also a rather intuitive boundary, because it stops the investigation when the treatment effect points in the opposite direction.

### 3.3 Analysis of Per Arm Sample Sizes

**FIGURE 4:**
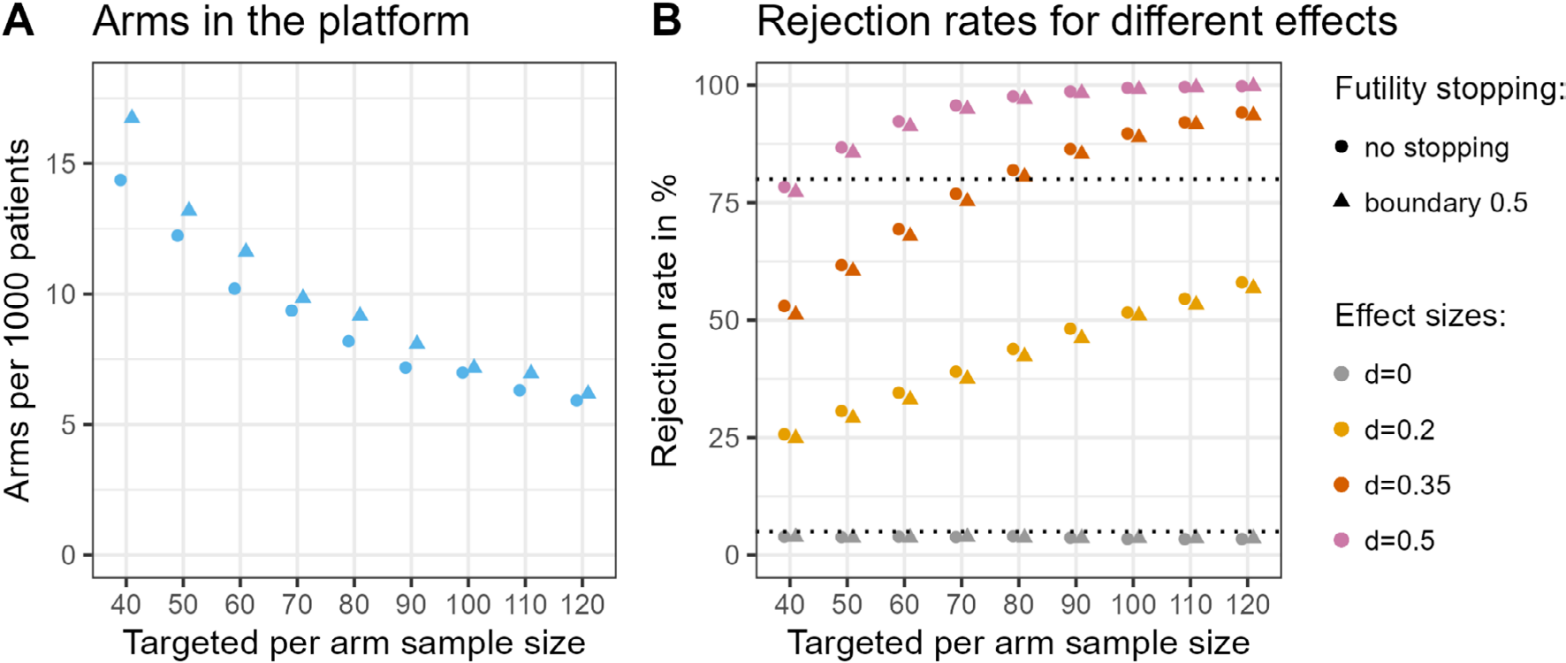
Experimental treatment arms per 1000 patients (without control arm) during the runtime of the platform trial, and rejection rates for different per‐arm sample sizes. All effect sizes are assumed to be equally likely, i.e., $\theta_{0}=\theta_{0.2}=\theta_{0.35}=\theta_{0.5}=0.25$. The circles give the values without the implementation of an interim analysis and the triangles the corresponding values when a futility boundary of 0.5 for the *p*‐value is applied. The sample size depicted on the *x*‐axis was examined in steps of 10. The small variation in the *x*‐direction is based on jittering for better readability. (A) shows the mean number of experimental treatment arms per 1000 patients. In (B), the percentage of rejected null hypotheses is depicted stratified by the four different investigated effect sizes. The rejection rate equals the type I error rate for d=0 and the power for the other values of d. The type I error rate is always controlled at 5%. This value is indicated by the lower dotted line. The higher dotted line highlights the 80% mark.

We simulated platform trials with different choices of the per treatment arm sample size. The ‘$\sqrt{k}$ allocation’ with a MAPC of 35% was used. We investigated designs without the option to stop for futility, as well as designs with an interim analysis after 50% of the targeted sample size and a futility boundary of 0.5 for the *p*‐value. The higher the targeted sample size per experimental treatment arm, the longer the investigation of this treatment arm takes, but the higher the power. The mean number of concurrently running experimental treatment arms goes from 2 for $n_{j}=40$ to 4 for $n_{j}=120$ in case of no interim analysis. The value for $n_{j}=80$ is 3.3. When a futility boundary of 0.5 is implemented, this number goes from 1.8 for $n_{j}=40$ to 3.9 for $n_{j}=120$ with 3.1 for the case of $n_{j}=80$. Figure 4B shows the power reached when targeting different per treatment arm sample sizes spanning from $n_{j}=40$ to $n_{j}=120$ with a step width of 10. The standardised number of experimental treatment arms (without control arm) that can be investigated is depicted in Figure 4A. The power is higher the higher the targeted sample size, but fewer arms can be investigated. When futility stopping is implemented, generally more arms can be investigated, and the power is lower. Applying the specified interim analysis, for the clinically relevant effect size d=0.35, a sample size of $n_{j}=70$ gives a power of 75.27%. When the futility boundary 0.5 is used, $n_{j}=80$ is the smallest investigated sample size with power above 80%, and $n_{j}=110$ the smallest with power above 90%. It is most cost‐efficient to use the smallest sufficient sample size for the desired power. However, the caveat with a smaller sample size is that it introduces higher variability, making it more likely to stop for futility.

### 3.4 Comparison to the Traditional Parallel Group Design

**FIGURE 5:**
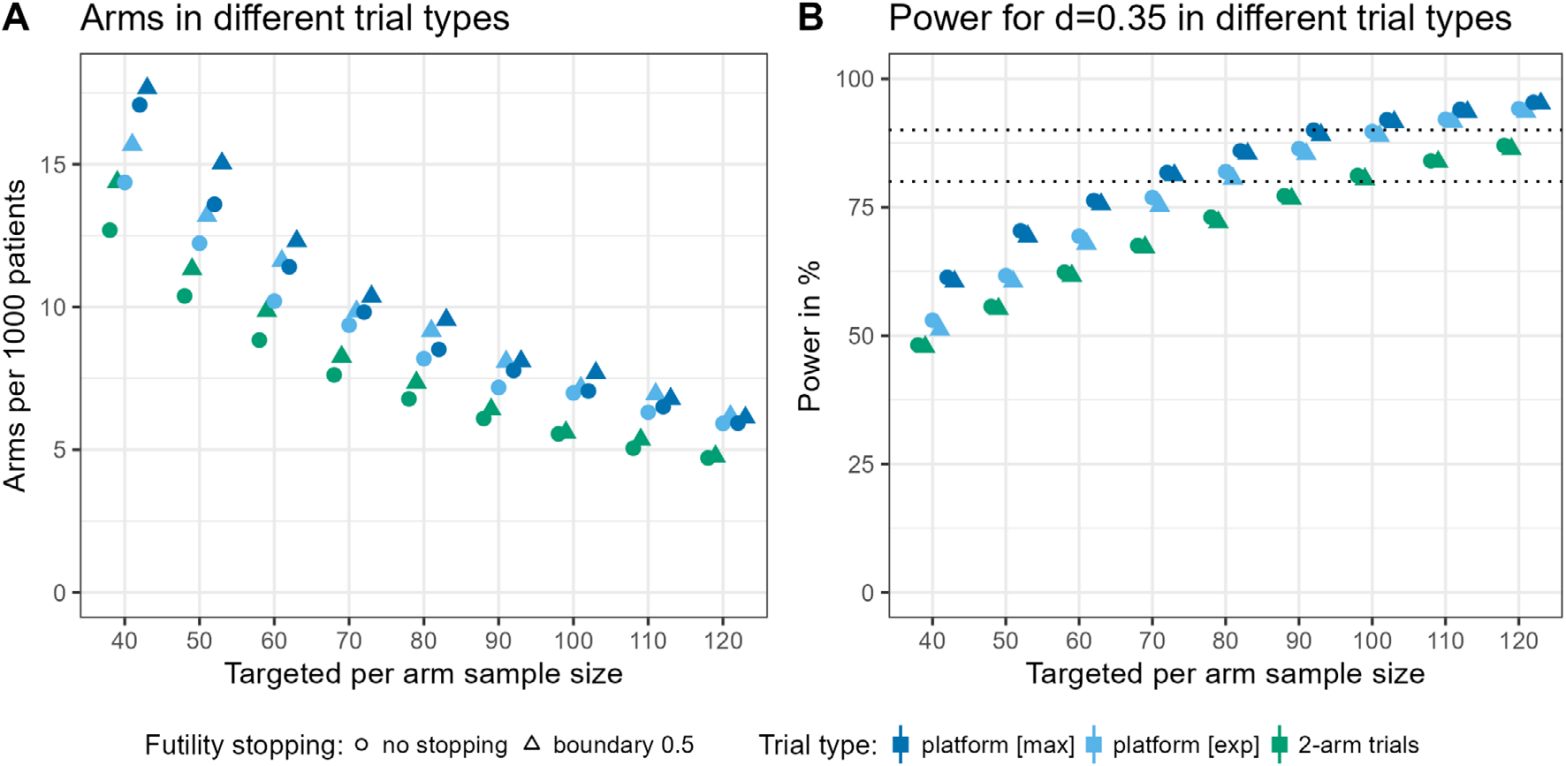
Comparison of operating characteristics in different trial types. All effect sizes are assumed to be equally likely, i.e., $\theta_{0}=\theta_{0.2}=\theta_{0.35}=\theta_{0.5}=0.25$. The circles give the values without the implementation of an interim analysis and the triangles the corresponding values when a futility boundary of 0.5 for the *p*‐value is applied. The sample size depicted on the x‐axis was examined in steps of 10. The small variation in the *x*‐direction is based on jittering for better readability. (A) shows the mean number of experimental treatment arms per 1000 patients for the three different trial types: Platform trial with maximum capacity utilisation, platform trial with expected load in the MDD case, and the traditional approach with a series of individual two‐arm randomised controlled trials. (B) gives the power for the same type of trials. The dotted lines indicate the 80% and 90% marks.

The classical approach for evaluating multiple treatments in the context of one disease, especially if more than one company is involved, is to use multiple randomised controlled trial designs. Typically, they have one experimental treatment arm with one control arm and 1:1 allocation between treatment and control. We simulated a series of such traditional trials in order to compare the operating characteristics to those of the designed platform trial. For the two‐arm trials, we make the same assumptions as in the platform design regarding sample size per experimental treatment arm, assumed effect sizes (and their distribution), and analysis method. We also included the option to stop for futility at an interim analysis after the primary endpoint is observed for half of the total sample size. Figure 5 shows the results of the comparison between sequential two‐arm trials and platform trials. The circles give the values for designs without interim analysis for futility and the triangles the values with a futility analysis after 50% of the sample size was reached and a futility boundary of 0.5 for the *p*‐value. Besides the platform trial running at the expected capacity, meaning three experimental treatment arms at the beginning of the platform and a 20% probability each month for a new arm to enter, we also included the maximum capacity scenario in the comparison, i.e., always running six experimental treatment arms in parallel (not counting control). This is notable as a platform running at maximum capacity shows the maximum benefit reached when using a platform trial, though this level of benefit is often not achieved in practice. Figure 5A shows the number of experimental treatment arms that can be investigated per 1000 patients. The inclusion of a futility analysis leads in all cases to a larger standardised number of experimental treatment arms that can be investigated. The platform trial running at maximum capacity enables the evaluation of the most arms. However, the difference compared to the more realistic platform setting diminishes with larger targeted sample sizes per treatment arm. This is due to treatment arms remaining in the platform longer when more patients are needed, which often results in more arms running in parallel. An important benefit of using platform trials is generated by the investigation of multiple treatment arms in parallel. By sharing the control group, fewer control patients are needed per treatment arm. The number of control comparators increases and, consequently, the power. This, however, only holds for certain allocation methods, such as the ‘$\sqrt{k}$ allocation’ method with a MAPC for control used here. If a 1:1:…:1 allocation were applied in the platform, the power would not increase as much, and one could use a traditional two‐arm trial with 1:1 allocation. With the platform design options used in the simulations, we consistently get higher power than for a series of two‐arm trials, even when the platform does not run at full capacity (see Figure 5B). The figure only shows the power for the clinically relevant effect size d=0.35 because this effect is the most relevant when deciding about the sample size and design of a trial. Rejection rates for the other effect sizes can be found in the Supporting Information. When using two‐arm trials, a per‐arm sample size of 100 is required to achieve a power of over 80% for the clinically relevant effect size. In a platform running at maximum capacity, a per‐arm sample size of 70 suffices, while in the more realistic workload setting, a per‐arm sample size of 80 would be needed. In a real‐world setting, however, the actual workload of the platform trial cannot be predicted with certainty. Hence, the decision about the targeted per‐arm sample size should not be made solely based on the power reached in a platform trial running at maximum capacity. For our specific use‐case, we would instead recommend a targeted per‐arm sample size of 80 when a power of 80% is desired.

### 3.5 Summary and Recommendations

Based on our simulations, we recommend using ‘$\sqrt{k}$ allocation’ with a MAPC of 35%, inclusion of time period as a factor, and futility stopping with at least a boundary of 0.5 (or even stricter, e.g., 0.25 if the platform is used for screening purposes). Like the futility boundary, the sample size also depends on the specific use case. It is especially influenced by the desired power. Note that the power reached is also dependent on the available concurrent experimental treatment arms, as we show in Section 3.4. One should therefore select a sample size that is a little higher than the minimum needed for the desired power.

In the Supporting Information, we explore additional simulation settings and present more operating characteristics. We show the number of control comparators per experimental treatment arm, the duration of arms and the mean number of concurrently running treatment arms without the control arm. For the comparison of trial types, we additionally report the proportion of patients randomised to control. In addition to the expected workload of the platform trial and an equal effect size distribution—which was the focus of the main manuscript—we provide in the Supporting Information all results for the pessimistic effect distribution as well. It also contains both effect size distributions for the maximal workload on the platform trial.

The Supporting Information results align with the ones presented in the main manuscript. This means that even though the values are slightly different, their relation is the same. For example, the option providing the highest power for the equal effect size distribution in a platform trial running at an expected workload also provides the highest power in the other investigated simulation settings. This holds for every subsection, and the recommendations are robust for the investigated scenarios.

Overall, the implementation of a platform trial provides both a higher power and a larger number of experimental treatment arms that can be tested with the same number of patients. It should therefore be preferred in settings when it is anticipated that multiple treatments would participate. If there is uncertainty about the potential availability of new treatments for investigation in a specific disease context and only very few are anticipated, the traditional two‐arm trial approach should be preferred. In such cases, the organisational effort involved in launching a platform would not be justified by the gain.

## 4 Discussion

This paper outlines the design considerations for a phase II platform trial in MDD [41], developed by an EU‐PEARL working group, with insights from an Innovation Task Force (ITF) meeting with the European Medicines Agency (EMA) [19, 42]. The corresponding platform trial is expected to launch in the near future in six European countries (Germany, Netherlands, Denmark, Spain, Italy, UK) [43]. Our primary focus was on the advantages of platform trials compared to separate two‐arm trials in the superiority setting. We conducted simulations to refine decision rules, randomisation methods, analysis strategies, and sample sizes. Note that the results of the simulations depend on the design type and assumptions. Different settings, such as a non‐inferiority setting, may yield different results. Due to the inherent complexities and random influences in platform trials, deterministic calculation of specific operating characteristics can become challenging, although some research has attempted this [37, 44, 45, 46, 47].

For reproducibility, our extensive simulation code is available on Github [34]. Meyer et al. [48] provide an overview of available software. While recently more generally applicable simulation and visualisation tools for platform trials have been proposed [49, 50], the code for the simulation studies is highly case‐specific, as are platform trials themselves. Often, substantial rewriting of code is required to apply it in other use cases.

Platform trials can differ in implemented group sequential boundaries, the addressing of multiplicity, error control, and many more. Design elements could even vary between treatment arms. For example, experimental treatment arms may target different sample sizes within the same trial, allowing sponsors with varying power targets (e.g., 80% vs. 90%) to participate in the same trial. However, the specific implications need further exploration. Assumptions about frequency distributions of the different targeted per‐arm sample sizes at different time points in the platform would be needed in order to investigate how this changes the respective concurrent control arms and thus also the power. In this platform trial, potential interim analyses are conducted to drop non‐promising treatment arms based on non‐binding futility‐stopping rules. The design could be further extended by incorporating group sequential boundaries both for efficacy and futility [51, 52].

Multiplicity adjustment in platform trials generally remains a topic of debate [37, 53, 54, 55, 56], particularly when comparing multiple treatment arms with a common control arm. No consensus has been reached yet [57, 58]. EU and US guidance documents do not provide definitive answers on when or how to adjust for multiplicity [59, 60, 61]. If separate trials were run, no adjustment for multiplicity would be required, and, e.g., in platform trials with individual control arms, the correlation of test statistics leads to the family‐wise error rate (FWER) being lower compared to running individual trials with individual controls [58]. Therefore, on the one hand, it could be argued that no adjustment for multiplicity should be required in a platform trial setting. On the other hand, the dependency in the test statistics impacts the decisions made and should be considered in confirmatory settings [53]. Another point of consideration in this regard is the interdependence of the individual hypotheses. Current practice suggests that no adjustment for multiplicity has to take place when the hypotheses are fairly unrelated, such as when treatments come from different sponsors and use different mechanisms of action. Conversely, when the hypotheses are related, such as when testing several doses of the same drug, adjustment should be made [62, 63]. In our platform trial, we do not adjust for multiplicity as we design an exploratory phase II trial, and the hypotheses tested can be considered inferentially independent.

Also, the handling of error rates is still discussed. For exploratory platform trials, the online control of the false discovery rate (FDR) has been suggested [64, 65]. If control of the FWER is crucial, methods proposed for multi‐armed multi‐stage clinical trials could be applied [44, 45, 47].

In the context of patients with TRD, we explored the potential for re‐randomising patients who did not benefit from the initial treatment. Re‐entry into the trial would require conditions such as constant treatment effects, independence, and adequate follow‐up or washout periods [66]. If different routes of administration were allowed, re‐entry could be restricted to another method of administration. However, the statistical implications would need further investigation.

We considered only oral administration of treatments as this is the predominant way in MDD. While other ways of administration, e.g., intravenous and intranasal, are promising, there are strong indications that the placebo response to these ways of administration differs considerably. Accordingly, if different routes of administration should be investigated in a platform trial, the use of a common control arm could lead to type I error inflation or reduced power. The impact of lacking similarity between control groups has, e.g., been investigated in the context of using historical controls [67]. We defined ‘domains’ of the platform trial as such drugs that can share a control group with blinding maintained, e.g., appropriately similar route of administration and dosing regimen. The need for several control arms would result in smaller allocation rates to individual experimental treatment arms and, thus, also longer durations per investigation. Overall, the benefit of using a platform trial would be split between the different ways of administration. Additionally, the absence of ‘objective’ outcome measures in MDD, with reliance on clinical rating scales [24], highlights the importance of careful trial design when different administration routes are involved.

The allocation methods investigated here are adaptive only in the sense that the allocation ratio to control is determined by the number of concurrently open treatment arms. More controversial adaptive approaches, such as response‐adaptive randomisation, are also implemented in some existing trials [68, 69, 70, 71, 72]. Response‐adaptive randomisation adjusts based on observed treatment effects. Its goal is to accelerate the evaluation of beneficial arms. While often all arms reach their targeted sample size eventually, sometimes only one good treatment is selected. Challenges arise if considering response‐adaptive randomisation in the presence of time trends, as response‐adaptive randomisation could lead to biased estimates if the analyses used are not appropriate for this situation. Current literature reflects considerations in more traditional two‐arm [73] and multi‐arm designs [74], and now also in the context of long‐running platform trials [75].

The statistical analysis model proposed will also account for the possibility of time trends in platform trials. The adjustment for potential time effects is achieved by including time period as a categorical factor in the ANCOVA model, ensuring control of the type I error rate and preservation of power without the need to specify the time trend structure. The results presented in the main paper's investigated scenarios assume no time trend. Specific investigations of stepwise time trends are presented in the Supporting Information (Section S1, Figures S1–S3) to illustrate the impact of strong time trends and changes in allocation ratios. Incorporating time period as a factor entails minimal power loss in the absence of time trends and can increase power when trends are present. More importantly, the assumption of no time trend being present is non‐testable in real world settings and failing to account for an actual time trend can result in a drastic reduction of power and severe inflation of the type I error rate for a specific treatment‐control comparison, depending on the strength of the time trend and change of allocation ratio. Our approach is consistent with the recent FDA draft guidance on master protocols, which recommends stratification by time period when randomisation ratios change [28]. As also pointed out by one reviewer, if the change is not specified a priori, this will impact both the type I and type II error rates. By changing the allocation ratio, the variance of the treatment effect estimator is implicitly also altered. Another issue in platform trials arises if patients can be randomised under different designs at the same time (e.g., when consent can be given to only a subgroup of treatments). In such cases, additionally, adjusting for the experimental design is necessary to address biases. A key principle in this respect is that for a specific treatment control comparison only subjects should be used, which could have been randomised to the treatment of interest—even when using concurrent controls [42, 76, 77].

Based on our simulations, we recommend a phase II platform trial in MDD utilising a ‘$\sqrt{k}$ allocation’ with a MAPC of 35%. We further suggest conducting futility analyses after 50% of the targeted sample size per treatment arm, using a futility boundary of 0.5 for the *p*‐value, and including time period as a covariate in the ANCOVA analysis. A sample size of 80 patients per experimental treatment arm is considered appropriate to reach a power of at least 80% for the clinically relevant effect size and relevant scenarios. However, it is crucial to thoroughly investigate the design choices for each specific use case, as they greatly impact performance characteristics. We advise conducting a larger number of simulations for specific points of interest. Ours were set to 10 000 repetitions each due to computational constraints and the broadly investigated scenarios.

In conclusion, our findings demonstrate that platform trials offer a more efficient approach to testing multiple treatments compared to separate two‐arm trials, resulting in greater power for the evaluation of individual arms. The benefit depends on how many experimental treatments are actually enrolling concurrently and the possible recruitment rate of the platform trial. If too many arms are enrolling at the same time, the duration of single arms might be prolonged. But, generally, due to the large site network established in platform settings, higher accrual rates can be achieved compared to single trials. The more enrolling treatments, the greater the overall cost benefit and benefit to the patients as fewer control data is required compared to the traditional approach. To maximise these benefits, interim futility analyses should be conducted to eliminate treatments with no or negligible effects early in the trial.

## Author Contributions

Conceptualization: Michaela Maria Freitag, Dario Zocholl, Stefan M. Gold, Martin Posch and Franz König. Methodology: Michaela Maria Freitag, Dario Zocholl, Martin Posch and Franz König. Software: Michaela Maria Freitag, Dario Zocholl and Elias Laurin Meyer. Conduct of simulation study: Michaela Maria Freitag. Supervision: Stefan M. Gold, Martin Posch and Franz König. Validation: Franz König. Visualisation: Michaela Maria Freitag. Writing – original draft: Michaela Maria Freitag and Franz König. Writing – review and editing: Michaela Maria Freitag, Dario Zocholl, Elias Laurin Meyer, Stefan M. Gold, Marta Bofill Roig, Heidi De Smedt, Martin Posch and Franz König.

## Conflicts of Interest

E.L.M. is a salaried employee of Berry Consultants. S.M.G. reports honoraria from Hexal and Streamed‐up. All other authors did not report any conflicts of interest.

## Supporting information


**Data S1:** Supporting Information.

## Data Availability

Data sharing not applicable to this article as no datasets were generated or analysed during the current study.
