# Supplementary material for: Design Considerations for a Phase II Platform Trial in Major Depressive Disorder
Source: Pharm Stat. 2025 Aug 27;24(5):e70025. doi: 10.1002/pst.70025 (PMC12384050; doi:10.1002/pst.70025)
Supplement: Supplementary file 1 — Data S1: Supporting Information. [file PST-24-0-s001.pdf]

Supplementary Material for Design Considerations for a Phase II Platform Trial in Major Depressive Disorder

Michaela Maria Freitag[1], Dario Zocholl[1], Elias Laurin Meyer[2,3], Stefan M. Gold[4,5,6,7], Marta Bofill Roig[2], Heidi De Smedt[8], Martin Posch[2], Franz König *[2], and on behalf of the EU-PEARL MDD Investigators[9]

[1]Charité – Universitätsmedizin Berlin, corporate member of Freie Universität Berlin, Humboldt-Universität zu Berlin, and Berlin Institute of Health, Institute of Biometry and Clinical Epidemiology, Berlin, Germany
[2]Medical University of Vienna, Center for Medical Data Science, Vienna, Austria
[3]Berry Consultants, Vienna, Austria
[4]Charité – Universitätsmedizin Berlin, corporate member of Freie Universität Berlin, Humboldt-Universität zu Berlin, and Berlin Institute of Health, Department of Psychiatry and Psychotherapy, Berlin, Germany
[5]Charité – Universitätsmedizin Berlin, corporate member of Freie Universität Berlin, Humboldt-Universität zu Berlin, and Berlin Institute of Health, Medical Department, Section Psychosomatics, Berlin, Germany
[6]DZPG, German Center for Mental Health
[7]Universitätsklinikum Hamburg-Eppendorf, INIMS, Hamburg, Germany
[8]Johnson & Johnson Innovative Medicine, Beerse, Belgium
[9]Membership of The EU-PEARL MDD Investigators is provided in the Acknowledgements

May 1, 2025

General simulation settings

We simulated platform trials for various scenarios. The different settings are introduced in Table 1 of Section 2.3.1 in the main manuscript, but some of the results are only presented in this supplementary material.
We use the terminology "expected workload" when we refer to a platform starting with a shared control arm, 3 treatment arms and a probability of 20% for a new arm to enter at the beginning of every month. "Maximal workload" refers to a platform always running with a shared control arm and 6 treatment arms, meaning whenever a treatment arm leaves the trial, a new one enters. For the "equal effect size distribution", all four effect sizes $d = 0$, $d = 0.2$, $d = 0.35$, $d = 0.5$ are equally likely, meaning $\theta_0 = \theta_{0.2} = \theta_{0.35} = \theta_{0.5} = 0.25$. For the "pessimistic effect size distribution", those probabilities are $\theta_0 = 0.5$, $\theta_{0.2} = 0.3$, $\theta_{0.35} = 0.1$, and $\theta_{0.5} = 0.1$.

1 Inclusion of time period as a factor in the ANCOVA model

1.1 Definition of analysis models

Time trends can influence the results of a clinical trial and lead to bias and loss of power if not properly addressed. One possible way to prevent this is to include the time period in the analysis. Time period refers to a section of time during which no treatment leaves the trial and no new treatment enters. In order

*Correspondence to franz.koenig@meduniwien.ac.at

to demonstrate the benefit of including time period as a categorical factor, we compared two ANCOVA models to test the null hypothesis of no treatment effect against the one-sided alternative hypothesis. Potential covariates to include are the baseline value and the time period. The first model, including time period, is the same one that is used in the main manuscript (see Section 2.2.1, equation 1):
The formal models M^j, $j \in \{1, ..., J\}$ can be written as

$$M^j : Y_{i,h_j} = \alpha_j + \beta_j G_{i,h_j} + \gamma_j X_{i,h_j} + \sum_{l=t_{j,\text{start}}+1}^{t_{j,\text{exit}}} \delta_l 1_{\{P_{i,h_j}=l\}} + e_{i,h_j} \quad (1)$$

The second model doesn't include the term for the time period and is defined as follows:

$$M^j : Y_{i,h_j} = \alpha_j + \beta_j G_{i,h_j} + \gamma_j X_{i,h_j} + e_{i,h_j} \quad (2)$$

The meanings of the terms are the same as defined in the main manuscript.

1.2 Rejection rates stratified by effect size using ANCOVA with and without including the factor time period

To evaluate the impact on type I error rate and power, we investigate a platform trial using the following scenario: No futility analysis was conducted and the expected workload of the platform trial was applied with 3 arms at the beginning of the platform and a probability of 20% per month for a new arm to enter. Table 1 illustrates the possible difference between including and not including the time period when a time trend is present. The trend in this exemplary simulation is assumed to be a step function on the 6-week MADRS score with steps at time points where treatments enter or leave the platform trial (i.e., at the beginning of every time period). The step width is set to an increase of about 1% and 5% of the variance of the 6-week MADRS score, which correspond to absolute values of 1.3 and 6.5, respectively (see Table 1). The modelled time trend only affects the follow-up values, as ranges for the baseline value are specified in the inclusion criteria. Please note that the time trend in the 6-week MADRS score impacts all active treatment arms and the control arm equally during the same period, i.e., the time trend is assumed to be consistent across all arms concerned. The values selected and also the time trend model are used exemplarily and only serve the purpose of illustrating the potential impact on type I error rate and power in settings where time trends are present, thus highlighting the the differences between ANCOVA models with and without the factor time period. All simulations presented in the main manuscript assume that there are no time trends. The key message is that the type I error rate may not be controlled when using ANCOVA models without the factor time period, and statistical power could also be negatively impacted. The reason is that treatment estimates can be biased in the presence of time trends, and the variance may be overestimated when not adjusting for the factor time period. As there is only a minimal loss in

Table 1: Rejection rates stratified by effect size: type I error rate (d=0) and power (stratified by $d = 0.2$, $d = 0.35$, and $d = 0.5$) of ANCOVA models with and without adjustment for time period as a factor. Either no time trend is assumed or a time trend modelled by a step function with a step width of either 1% or 5% of the 6-week MADRS score variance at each time point when the time period changes due to adding/dropping of treatment arms. Only the 6-week MADRS score is affected by the time trend.

	no time trend		step-wise time trend 1%		step-wise time trend 5%	
Effect Size d	without time period	with time period	without time period	with time period	without time period	with time period
0	4.20	4.32	5.56	4.24	8.03	4.34
0.2	43.9	43.9	39.1	43.0	26.0	43.6
0.35	81.8	81.1	70.3	82.0	42.4	82.4
0.5	97.8	97.6	86.7	97.7	56.0	97.4

power when using an ANCOVA model with the factor time period in the absence of any time trend, and the power is preserved even if there are time trends, regardless of their strength, we strongly recommend including time period as a factor in the ANCOVA for real-world applications. This is because one can never be certain if there really is no time trend in real-world settings. This suggestion aligns with the FDA guidance document on platform trials. Omitting the factor time period can lead to severe inflation of type I error rate and also a substantial loss in power, depending on the strength of the time trend, see Table 1. The investigation of the impact of different shapes of time trend is beyond the scope of this paper. These simulations are intended solely to highlight why it is important to include the factor time period.

In Table 2, we also show the results if both the baseline and the follow-up values are affected in the same way by the time trend. There is a loss in power in the ANCOVA model without the factor time period compared to the ANCOVA model including the factor time trend. However, it is less dramatic even when assuming a large step size of about 5% of the variance of the 6-week MADRS score. The reason is that in such cases the covariate baseline acts as a kind of proxy for the factor time period, as the baseline values of patients included in different time periods will quite differ.

Table 2: Rejection rates stratified by effect size: type I error rate (d=0) and power (stratified by $d = 0.2$, $d = 0.35$, and $d = 0.5$) of ANCOVA models with and without adjustment for time period as a factor. Either no time trend is assumed or a time trend modelled by a step function with a step width of 5% of the 6-week MADRS score variance at each time point when the time period changes due to adding/dropping of treatment arms. Both, the baseline and the 6-week MADRS scores are affected by the time trend.

	no time trend		step-wise time trend 5%	
Effect Size d	without time period	with time period	without time period	with time period
0	4.79	4.73	4.76	4.83
0.2	43.8	43.9	41.3	43.3
0.35	82.3	82.2	79.9	81.6
0.5	97.6	97.5	95.9	97.4

1.3 Rejection rates for treatment arm 1 with and without including the factor time period

When focusing on a specific treatment arm, the change in allocation ratio introduced by adding or removing other arms influences the operating characteristics when not adjusting for the factor time period. In Figures 1, 2, and 3, we examine the operating characteristics of a single specific treatment arm, e.g., treatment arm 1. For the simulation presented in Figure 1 we assumed a platform trial starting with three treatment arms (treatment arm 1 being part of them), corresponding to an initial allocation to control of 1:$\sqrt{3}$. After half of the desired sample size is recruited ($n_1 = 40$), the number of treatment arms and hence the allocation to control changes either to 1 (purple line) or $\sqrt{6}$ (yellow line) or stays the same (green line). The solid lines represent results without adjustment for the factor time period, while the dotted lines represent results with such adjustment. Overall, we observe an impact on type I error rate, with potentially substantial inflation, depending on the direction and magnitude of the allocation ratio change, when not adjusting for time period. The power is similarly affected. Even when there is no change in the allocation ratio (green line), the type I error is controlled, but the power is reduced, due to higher residual variance. When adjusting for the factor time period, the type I error rate remains controlled, and the power is preserved over a wide range of trend sizes. Figures 2 and 3 show results for scenarios starting with 1 or 6 treatment arms, respectively. Importantly, the type I error rates (including any potential inflation) and power would be identical for another treatment arm, even if it was added later in the trial. This holds as long as the number of active treatments is the same at the beginning of each relevant time period.

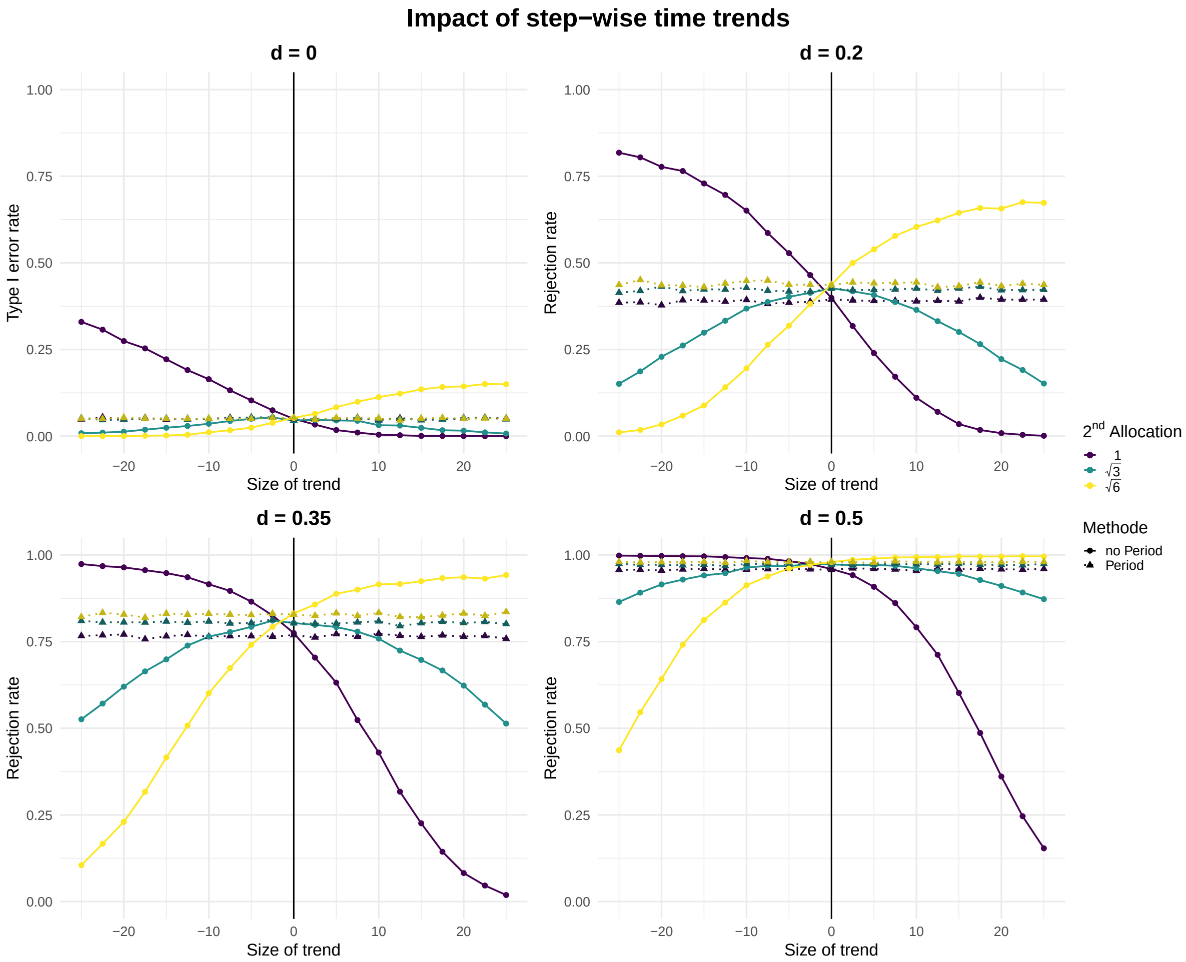

Figure 1: Rejection rate for treatment arm 1 assuming different values of the step-wise time trend (x-axis) stratified by treatment effect. It equals the type I error rate for $d = 0$ and the power for the other values of d. The vertical lines mark the absence of a time trend. This scenario starts with three treatment arms, i.e., an allocation to control of 1:$\sqrt{3}$. After half of the desired sample size is recruited ($n_1 = 40$), the number of treatment arms and consequently the allocation to control changes either to 1 (purple line) or $\sqrt{6}$ (yellow line) or stays the same (green line). The solid lines show the results when not adjusting for time period, and the dotted lines show the results when adjusting for time period.

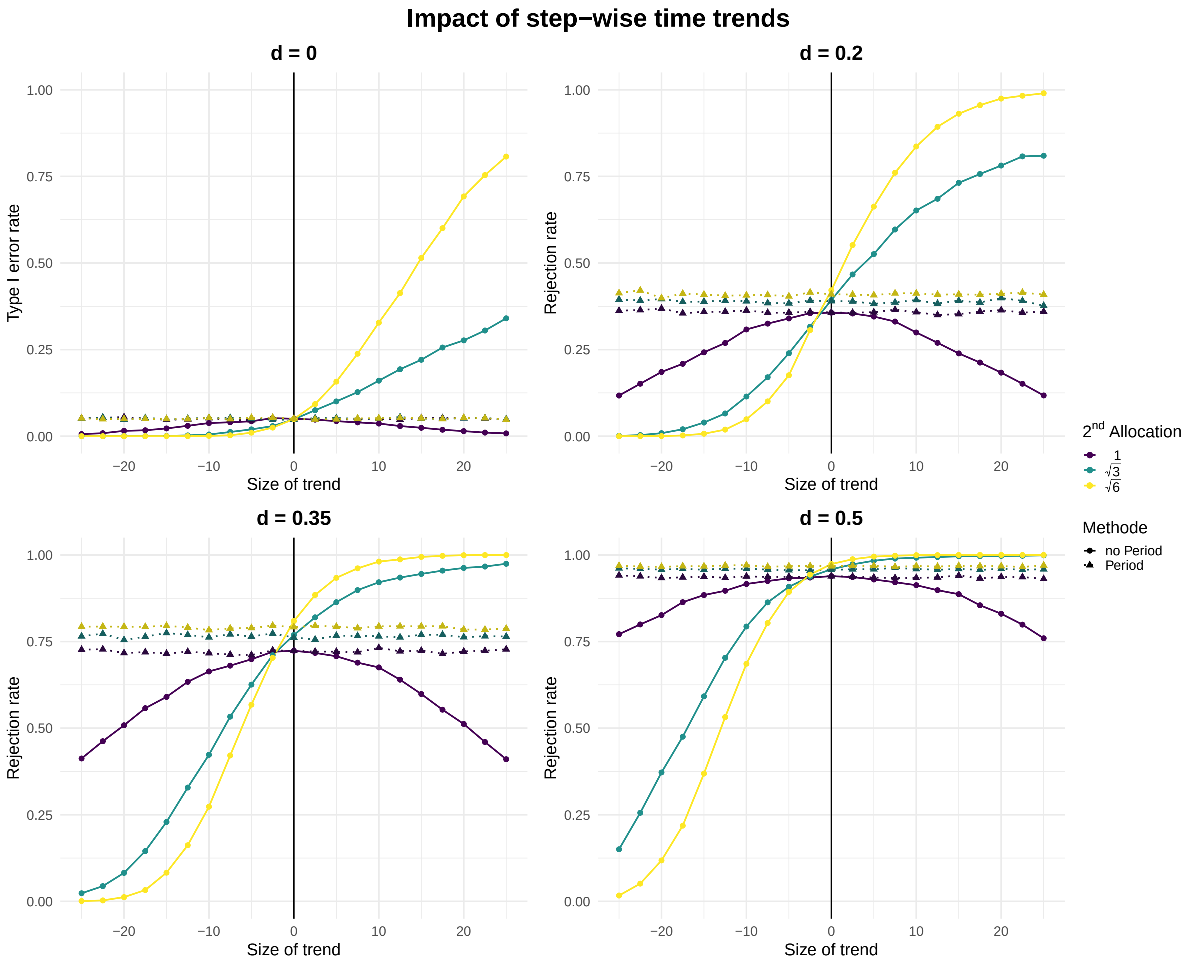

Figure 2: Rejection rate for treatment arm 1 assuming different values of the step-wise time trend (x-axis) stratified by treatment effect. It equals the type I error rate for $d = 0$ and the power for the other values of d. The vertical lines mark the absence of a time trend. This scenario starts with one treatment arm, i.e., an allocation to control of 1:1. After half of the desired sample size is recruited ($n_1 = 40$), the number of treatment arms and consequently the allocation to control either stays the same (purple line) or changes to $\sqrt{3}$ (green line) or $\sqrt{6}$ (yellow line). The solid lines show the results when not adjusting for time period, and the dotted lines show the results when adjusting for time period.

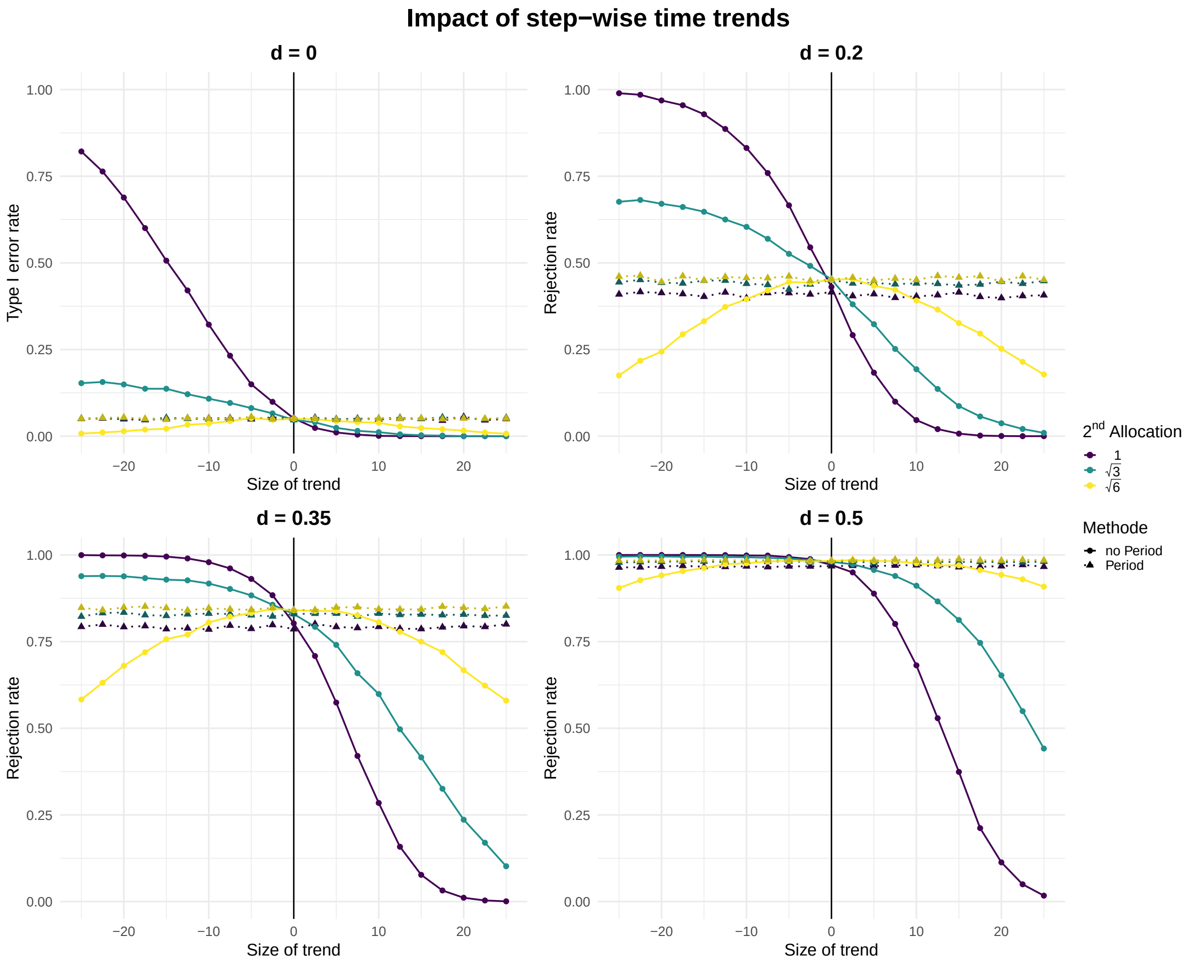

Figure 3: Rejection rate for treatment arm 1 assuming different values of the step-wise time trend (x-axis) stratified by treatment effect. It equals the type I error rate for $d = 0$ and the power for the other values of d. The vertical lines mark the absence of a time trend. This scenario starts with six treatment arms, i.e., an allocation to control of 1:$\sqrt{6}$. After half of the desired sample size is recruited ($n_1 = 40$), the number of treatment arms and consequently the allocation to control changes either to 1 (purple line) or $\sqrt{3}$ (green line) or stays the same (yellow line). The solid lines show the results when not adjusting for time period, and the dotted lines show the results when adjusting for time period.

2 Selection of the Minimum Allocation Probability to Control (MAPC)

In order to ensure that the allocation to control is always above a certain percentage, one can set a Minimum Allocation Probability to Control (MAPC). Due to the nature of MDD, we considered doing so. In this disease type, a lower likelihood of receiving a placebo is linked to an increase in the placebo response. From a clinical perspective, having an allocation probability to control of at least one-third is recommended for limiting the placebo response. The statistical implications of different MAPC were investigated by simulations and are summarised here. Note that a MAPC of 0.275 corresponds to having no lower limit at all but the standard $\sqrt{k}$ allocation because the platform trial only allows for a maximum of 6 concurrently running treatment arms k plus one shared control arm. A MAPC of 0.5, on the other hand, corresponds to the k allocation, i.e., always half of all patients receiving the control (placebo) treatment.

The per-arm sample size was set to 80, no interim analyses were conducted and an equal effect size distribution was assumed, i.e., the probability of each effect size was set to $\theta_0 = \theta_{0.2} = \theta_{0.35} = \theta_{0.5} = 0.25$. We investigated platform trials under the expected workload (i.e., starting with three arms and a probability of 20% per month for new arms to enter), and under the maximum workload (i.e., always 6 treatment arms running concurrently). Figures 4, 6, 8, and 10 show the implications of different MAPC on the standardised number of arms that can be tested in the platform trial and on the overall rejection rates. Figures 5, 7, 9, and 11 show the implications of different MAPC on the size of the individual control groups and on the duration of individual treatment arms. Tables 3 and 4 give the mean number of concurrently running experimental treatment arms without the control arm.

2.1 Results for the expected workload scenario

A) Equal effect size distribution

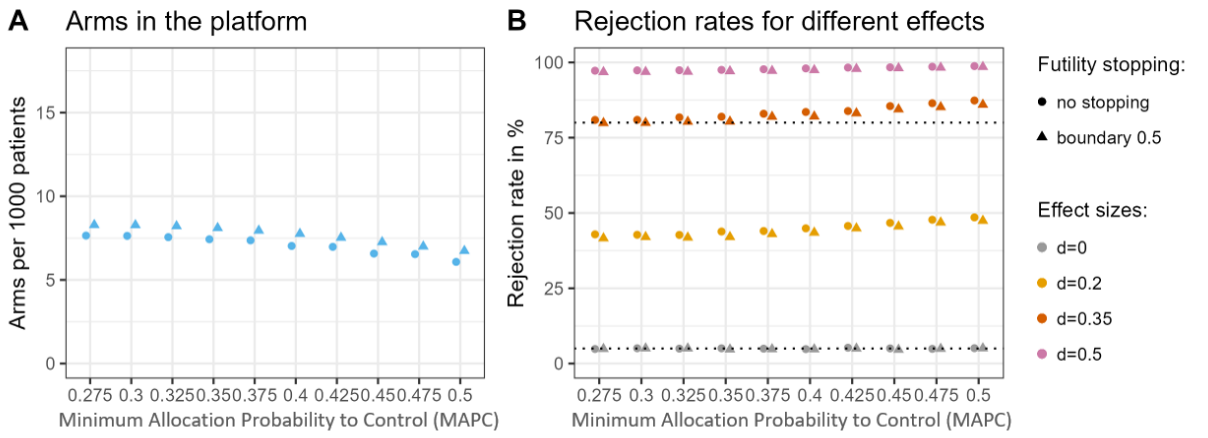

Figure 4: Standardised number of experimental treatment arms (without control arm) during the runtime of the platform trial and rejection rates for different Minimum Allocation Probabilities to Control (MAPC). In the depicted scenario, all effect sizes are assumed to be equally likely and an expected workload is assumed. (A) gives the number of experimental treatment arms (without control arm) standardised for 1000 patients in the platform. In (B), the percentage of rejected null hypotheses stratified by treatment effect is depicted. It equals the type I error rate for $d = 0$ and the power for the other values of d. The dotted lines mark the 5% and the 80% levels.

Table 3: Mean number of concurrently running experimental treatment arms (without control arm) in platform trials running at expected capacity with an equal effect size distribution for different Minimum Allocation Probabilities to Control (MAPC). The first line gives the values if no futility stopping is implemented and the second line gives the ones for a futility boundary of 0.5 for the p-value.

	Minimum Allocation Probabilities to Control (MAPC)									
Futility rule	0.275	0.3	0.325	0.35	0.375	0.4	0.425	0.45	0.475	0.5
no stop	3.09	3.11	3.17	3.27	3.38	3.49	3.61	3.72	3.81	3.92
0.5	2.92	2.93	2.99	3.07	3.18	3.30	3.44	3.57	3.67	3.79

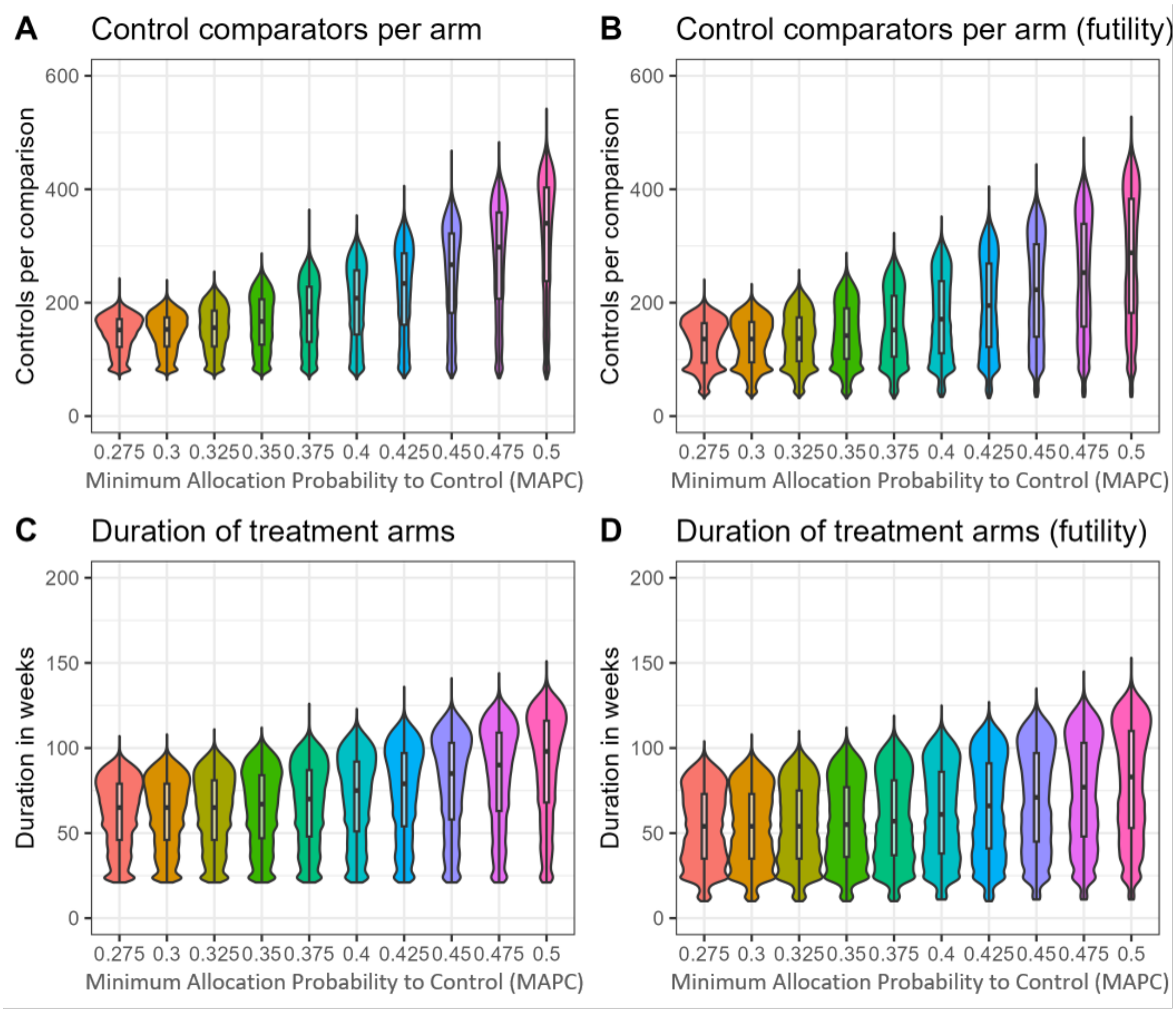

Figure 5: Duration of experimental treatment arms and number of control comparators per experimental treatment arm for different Minimum Allocation Probabilities to Control (MAPC). In the depicted scenario, all effect sizes are assumed to be equally likely and an expected workload is assumed. (A) and (C) give the results without implementation of a futility analysis, and (B) and (D) for a futility boundary of 0.5 for the p-value. The values are depicted as violin plots with integrated box plots.

B) Pessimistic effect size distribution

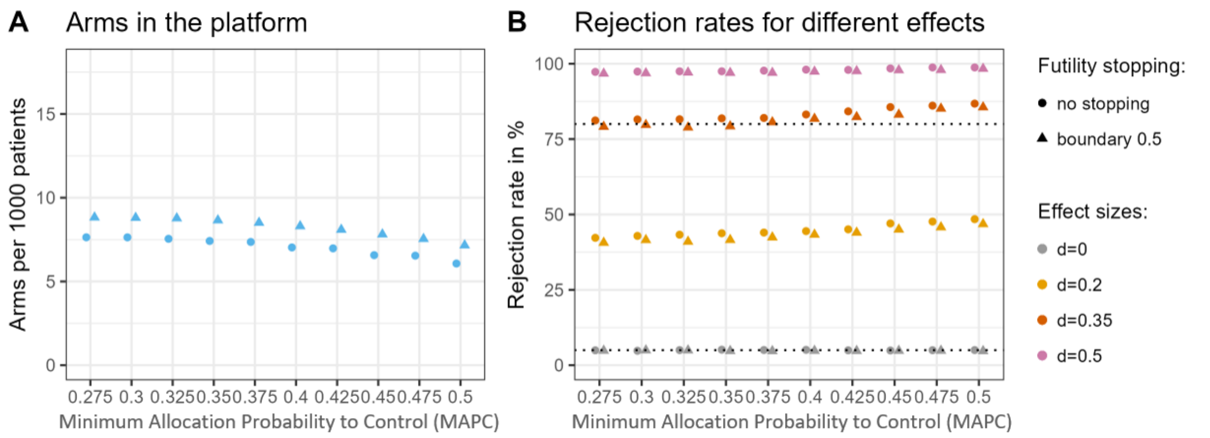

Figure 6: Standardised number of experimental treatment arms (without control arm) during the runtime of the platform trial and rejection rates for different Minimum Allocation Probabilities to Control (MAPC). In the depicted scenario, a pessimistic effect size distribution and an expected workload are assumed. (A) gives the number of experimental treatment arms (without control arm) standardised for 1000 patients in the platform. In (B), the percentage of rejected null hypotheses stratified by treatment effect is depicted. It equals the type I error rate for $d = 0$ and the power for the other values of d. The dotted lines mark the 5% and the 80% levels.

Table 4: Mean number of concurrently running experimental treatment arms (without control arm) in platform trials running at expected capacity with a pessimistic effect size distribution for different Minimum Allocation Probabilities to Control (MAPC). The first line gives the values if no futility stopping is implemented and the second line gives the ones for a futility boundary of 0.5 for the p-value.

	Minimum Allocation Probabilities to Control (MAPC)									
Futility rule	0.275	0.3	0.325	0.35	0.375	0.4	0.425	0.45	0.475	0.5
no stop	3.09	3.11	3.17	3.26	3.38	3.49	3.60	3.72	3.81	3.91
0.5	2.76	2.79	2.82	2.90	3.01	3.14	3.26	3.39	3.52	3.66

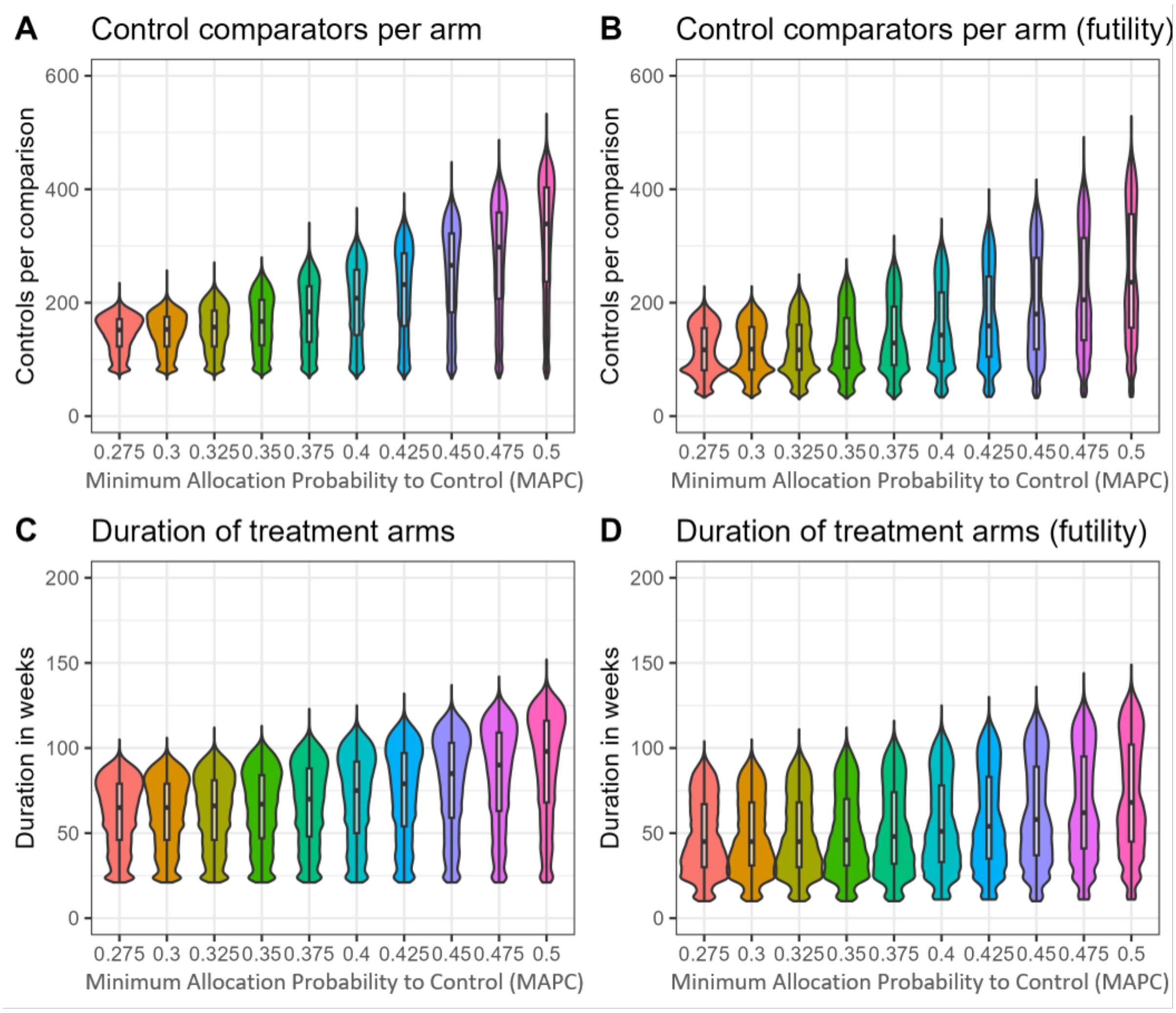

Figure 7: Duration of experimental treatment arms and number of control comparators per experimental treatment arm for different Minimum Allocation Probabilities to Control (MAPC). In the depicted scenario, a pessimistic effect size distribution and an expected workload are assumed. (A) and (C) give the results without implementation of a futility analysis, and (B) and (D) for a futility boundary of 0.5 for the p-value. The values are depicted as violin plots with integrated box plots.

2.2 Results for the maximal workload scenario

A) Equal effect size distribution

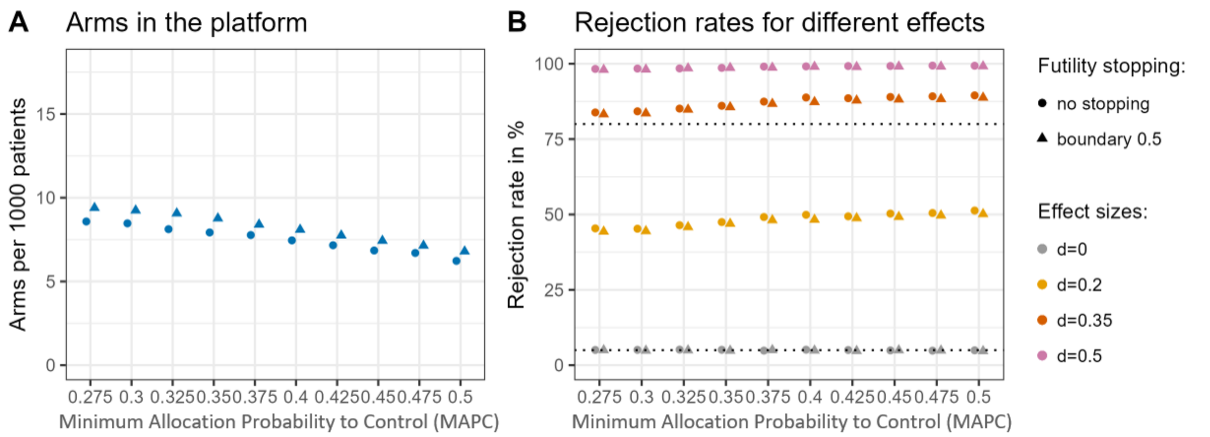

Figure 8: Standardised number of experimental treatment arms (without control arm) during the run-time of the platform trial and rejection rates for different Minimum Allocation Probabilities to Control (MAPC). In the depicted scenario, all effect sizes are assumed to be equally likely and a maximal workload is assumed. (A) gives the number of experimental treatment arms (without control arm) standardised for 1000 patients in the platform. In (B), the percentage of rejected null hypotheses stratified by treatment effect is depicted. It equals the type I error rate for $d = 0$ and the power for the other values of d. The dotted lines mark the 5% and the 80% levels.

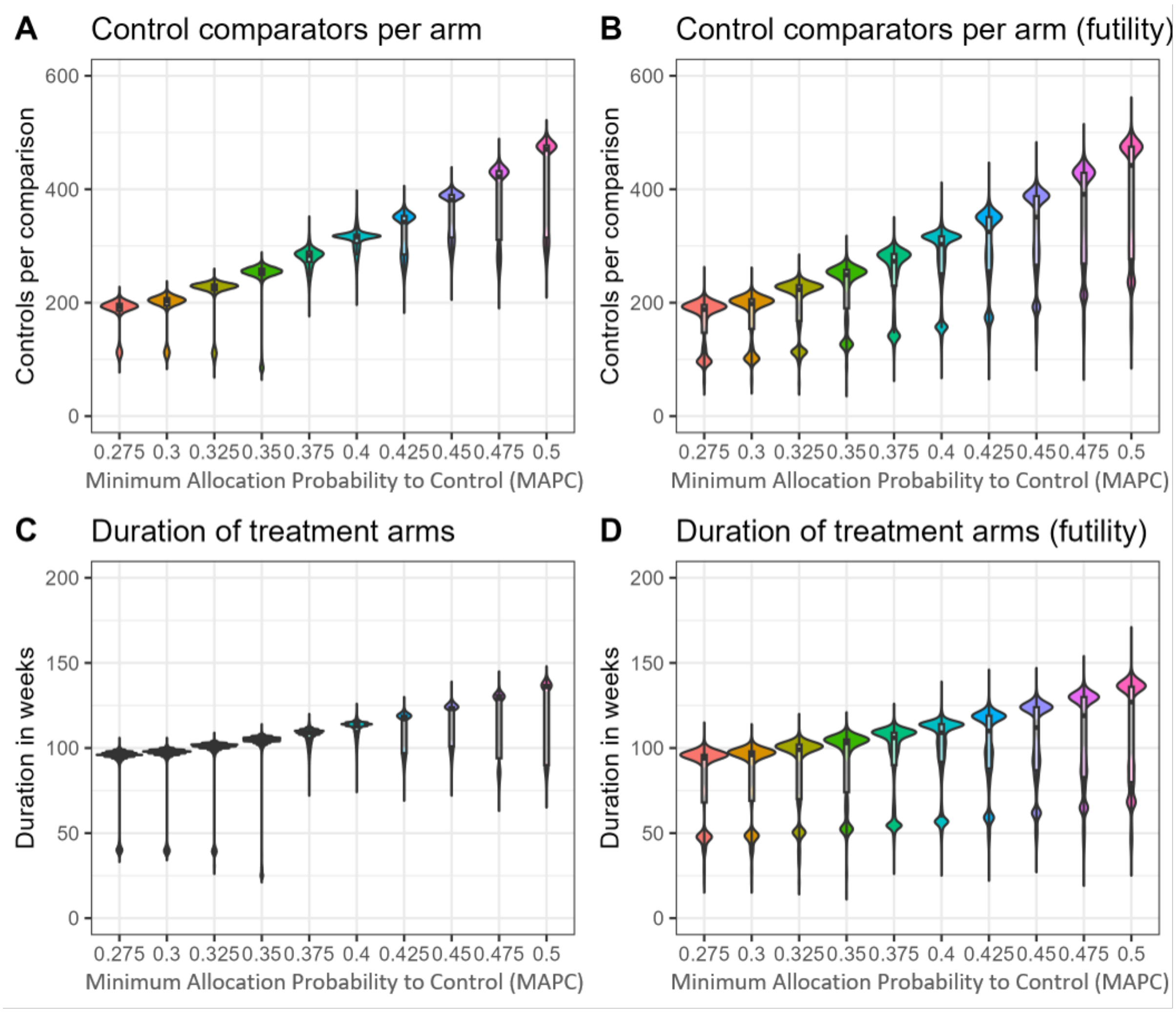

Figure 9: Duration of experimental treatment arms and number of control comparators per experimental treatment arm for different Minimum Allocation Probabilities to Control (MAPC). In the depicted scenario, all effect sizes are assumed to be equally likely and a maximal workload is assumed. (A) and (C) give the results without implementation of a futility analysis, and (B) and (D) for a futility boundary of 0.5 for the p-value. The values are depicted as violin plots with integrated box plots.

B) Pessimistic effect size distribution

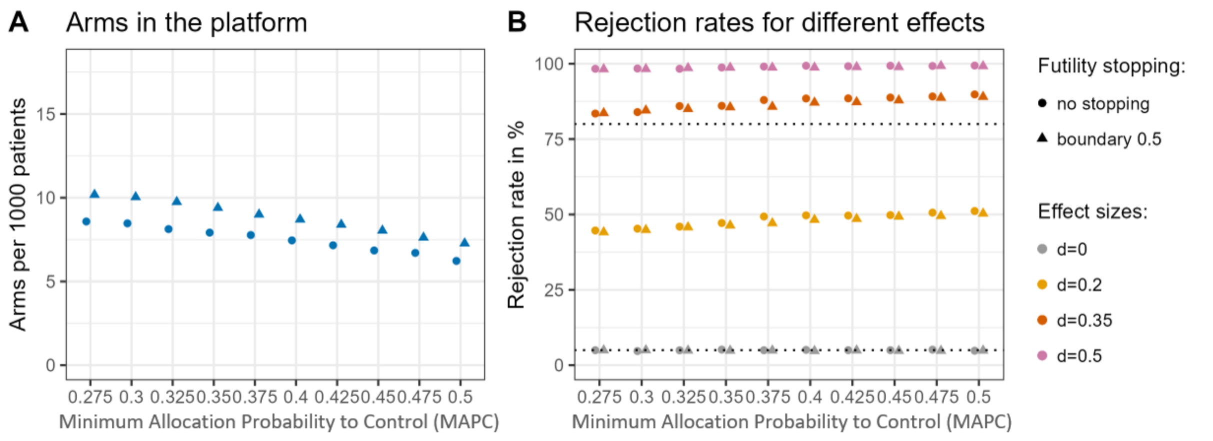

Figure 10: Standardised number of experimental treatment arms (without control arm) during the runtime of the platform trial and rejection rates for different Minimum Allocation Probabilities to Control (MAPC). In the depicted scenario, a pessimistic effect size distribution and a maximal workload are assumed. (A) gives the number of experimental treatment arms (without control arm) standardised for 1000 patients in the platform. In (B), the percentage of rejected null hypotheses stratified by treatment effect is depicted. It equals the type I error rate for $d = 0$ and the power for the other values of d. The dotted lines mark the 5% and the 80% levels.

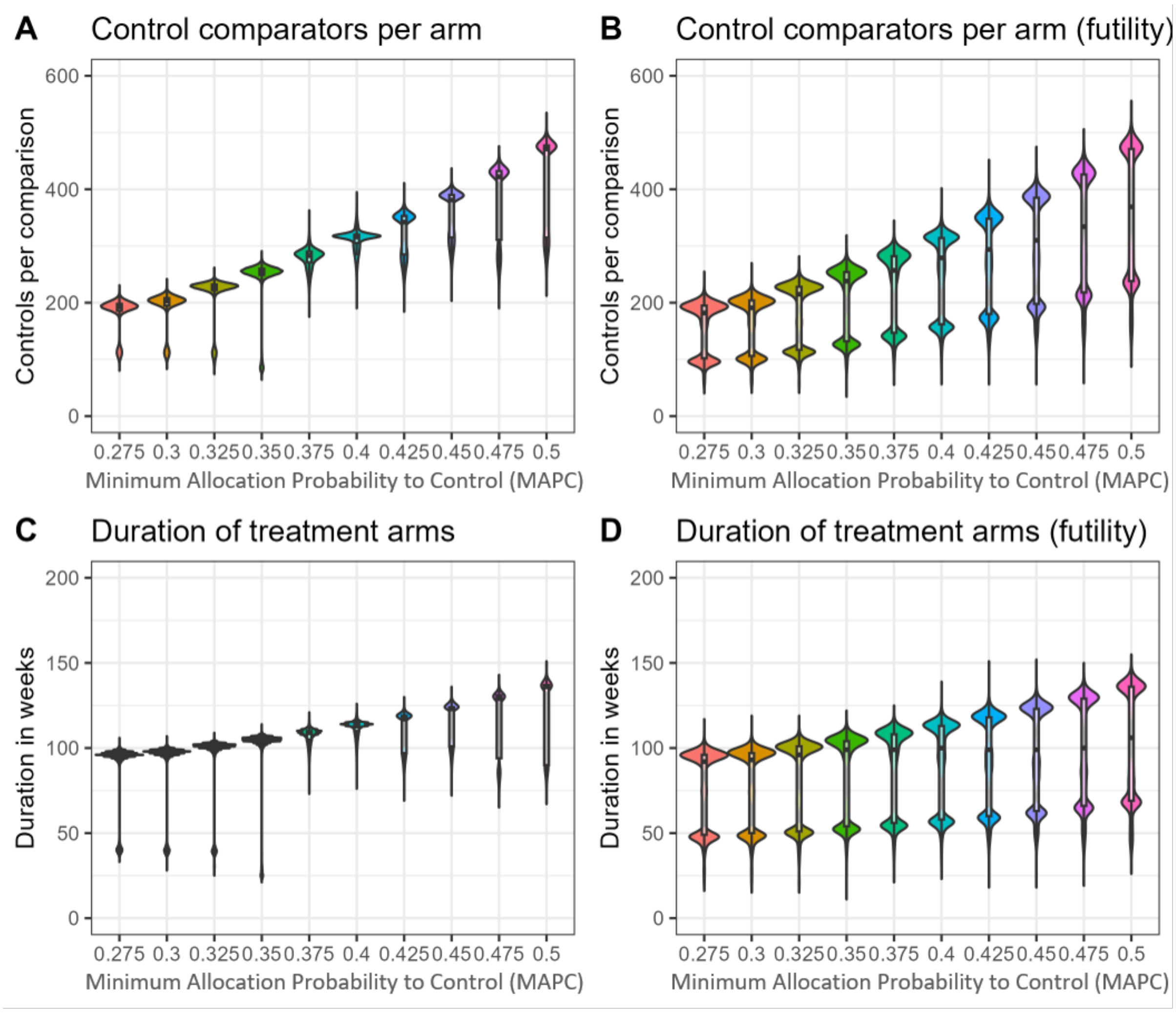

Figure 11: Duration of experimental treatment arms and number of control comparators per experimental treatment arm for different Minimum Allocation Probabilities to Control (MAPC). In the depicted scenario, a pessimistic effect size distribution and a maximal workload are assumed. (A) and (C) give the results without implementation of a futility analysis, and (B) and (D) for a futility boundary of 0.5 for the p-value. The values are depicted as violin plots with integrated box plots.

2.3 Conclusion on the MAPC

A higher MAPC implies a decreasing number of patients on treatment and a growing number on control. The duration of treatment arms also extends as the MAPC rises (see Figures 5, 7, 9, and 11). Figures 4, 6, 8, and 10 show that it also implies increasing power while the number of arms that can be tested decreases.
In our application, the platform trial should be designed for a phase II trial. In this setting, it is important to screen as many treatments as possible in a time frame as short as possible. For this reason, we selected 35% as the MAPC. This value is just above the one-third level the clinicians recommended as a lower limit and allows for the most arms to be tested. Additionally, the power is still quite high and does not differ much from the power reached by implementing slightly higher MAPC.

3 Additional scenarios for the design options presented in the main paper

In the main paper, results for platform trials running at an expected capacity are presented. We always show the standardised number of treatment arms and the rejection rates. Here, we additionally give the duration of treatment arms and the size of the individual concurrent control groups for the different analyses. The accrual rate was assumed to have a mean of 7 patients per month.
For the comparison of platform trials to a series of two-arm trials, a scenario for the maximal workload is presented. Here we show the results for this scenario in the other investigations. Also, with the exception of the futility section, in the main manuscript, only results for the equal distribution of effect sizes are shown. However, there are many possible effect size distributions that can be assumed. Here we present results for the more pessimistic effect size scenario that is also applied in the futility section of the main paper. If not otherwise stated, the per-arm sample size is set to 80, and the futility boundary is set to 0.5 for the p-value at the interim analysis.

3.1 Additional scenarios for the selection of the allocation method

3.1.1 Results for the expected workload scenario

A) Equal effect size distribution

Table 5: Mean number of concurrently running experimental treatment arms (without control arm) in platform trials running at expected capacity with an equal effect size distribution for different allocation methods. The first line gives the values if no futility stopping is implemented, and the second line gives the ones for a futility boundary of 0.5 for the p-value.

	Allocation method			
Futility rule	balanced	k	$\sqrt{k}$	$\sqrt{k}$ with MAPC
no stop	2.42	3.92	3.11	3.26
0.5	2.24	3.80	2.93	3.07

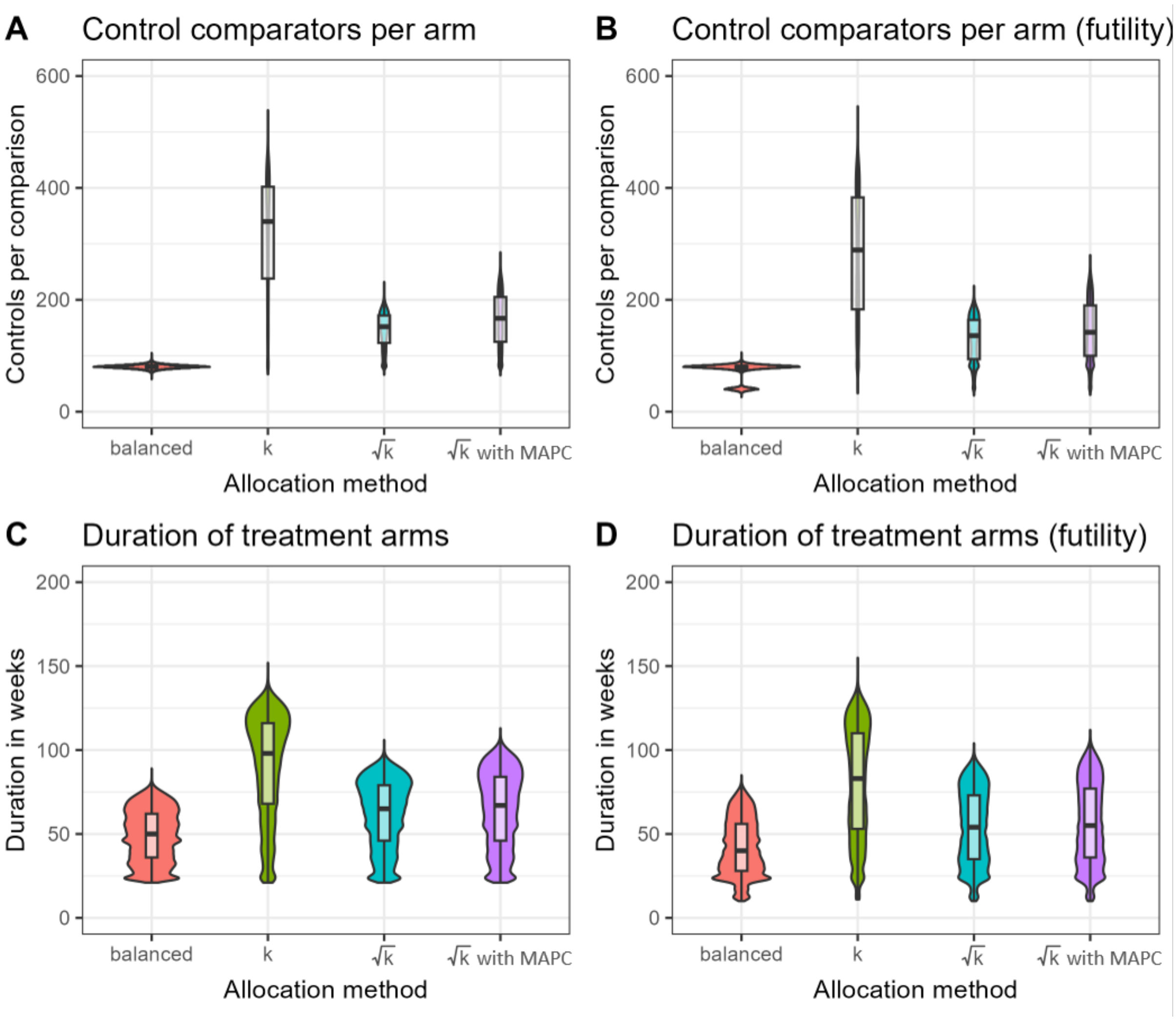

Figure 12: Duration of experimental treatment arms and number of control comparators per experimental treatment arm for different allocation methods. In the depicted scenario, all effect sizes are assumed to be equally likely and an expected workload is assumed. (A) and (C) give the results without implementation of a futility analysis, and (B) and (D) for a futility boundary of 0.5 for the p-value. The values are depicted as violin plots with integrated box plots.

B) Pessimistic effect size distribution

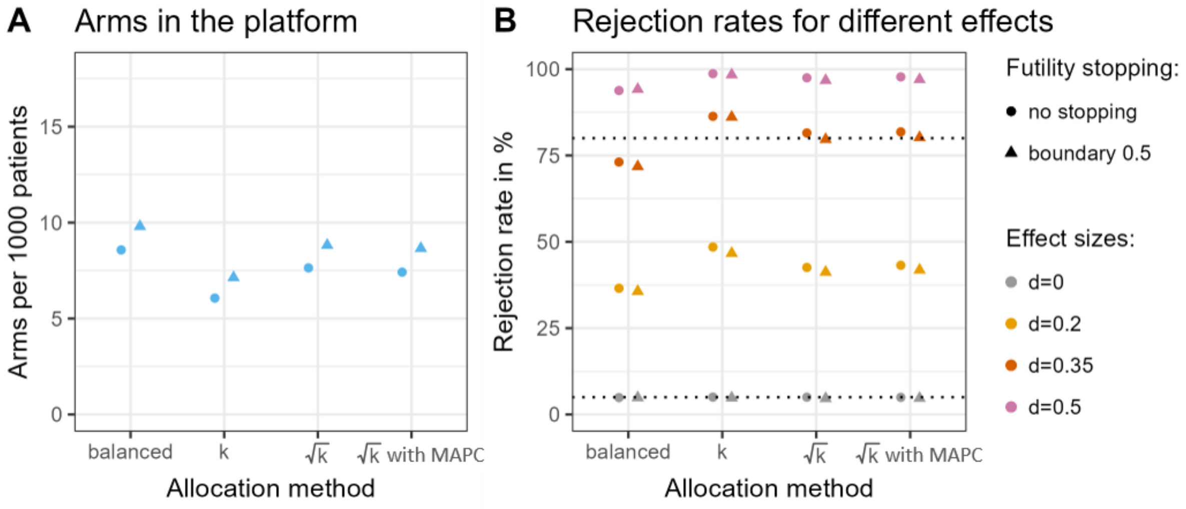

Figure 13: Standardised number of experimental treatment arms (without control arm) during the run-time of the platform trial and rejection rates for different allocation methods. In the depicted scenario, a pessimistic effect size distribution and an expected workload are assumed. (A) gives the number of experimental treatment arms (without control arm) standardised for 1000 patients in the platform. In (B), the percentage of rejected null hypotheses stratified by treatment effect is depicted. It equals the type I error rate for $d = 0$ and the power for the other values of d. The dotted lines mark the 5% and the 80% levels.

Table 6: Mean number of concurrently running experimental treatment arms (without control arm) in platform trials running at expected capacity with a pessimistic effect size distribution for different allocation methods. The first line gives the values if no futility stopping is implemented, and the second line gives the ones for a futility boundary of 0.5 for the p-value.

	Allocation method			
Futility rule	balanced	k	$\sqrt{k}$	$\sqrt{k}$ with MAPC
no stop	2.41	3.91	3.10	3.26
0.5	2.13	3.66	2.77	2.91

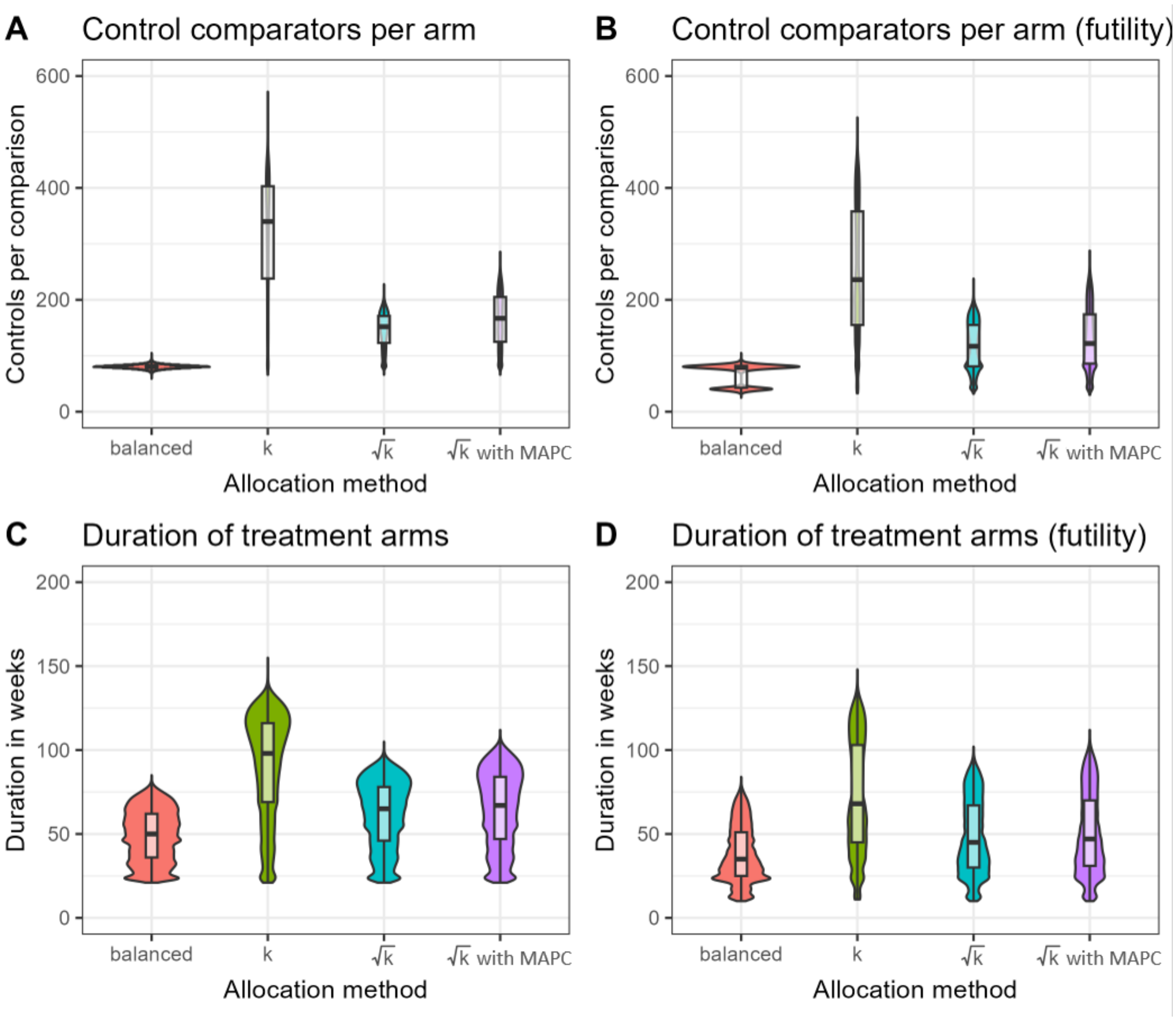

Figure 14: Duration of experimental treatment arms and number of control comparators per experimental treatment arm for different allocation methods. In the depicted scenario, a pessimistic effect size distribution and an expected workload are assumed. (A) and (C) give the results without implementation of a futility analysis, and (B) and (D) for a futility boundary of 0.5 for the p-value. The values are depicted as violin plots with integrated box plots.

3.1.2 Results for the maximal workload scenario

A) Equal effect size distribution

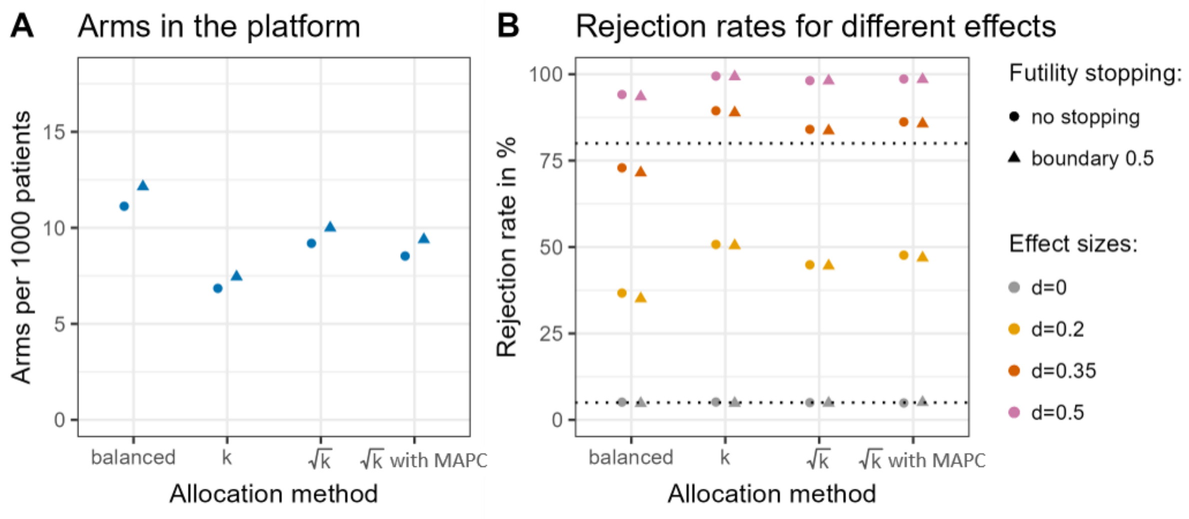

Figure 15: Standardised number of experimental treatment arms (without control arm) during the run-time of the platform trial and rejection rates for different allocation methods. In the depicted scenario, all effect sizes are assumed to be equally likely and a maximal workload is assumed. (A) gives the number of experimental treatment arms (without control arm) standardised for 1000 patients in the platform. In (B), the percentage of rejected null hypotheses stratified by treatment effect is depicted. It equals the type I error rate for $d = 0$ and the power for the other values of d. The dotted lines mark the 5% and the 80% levels.

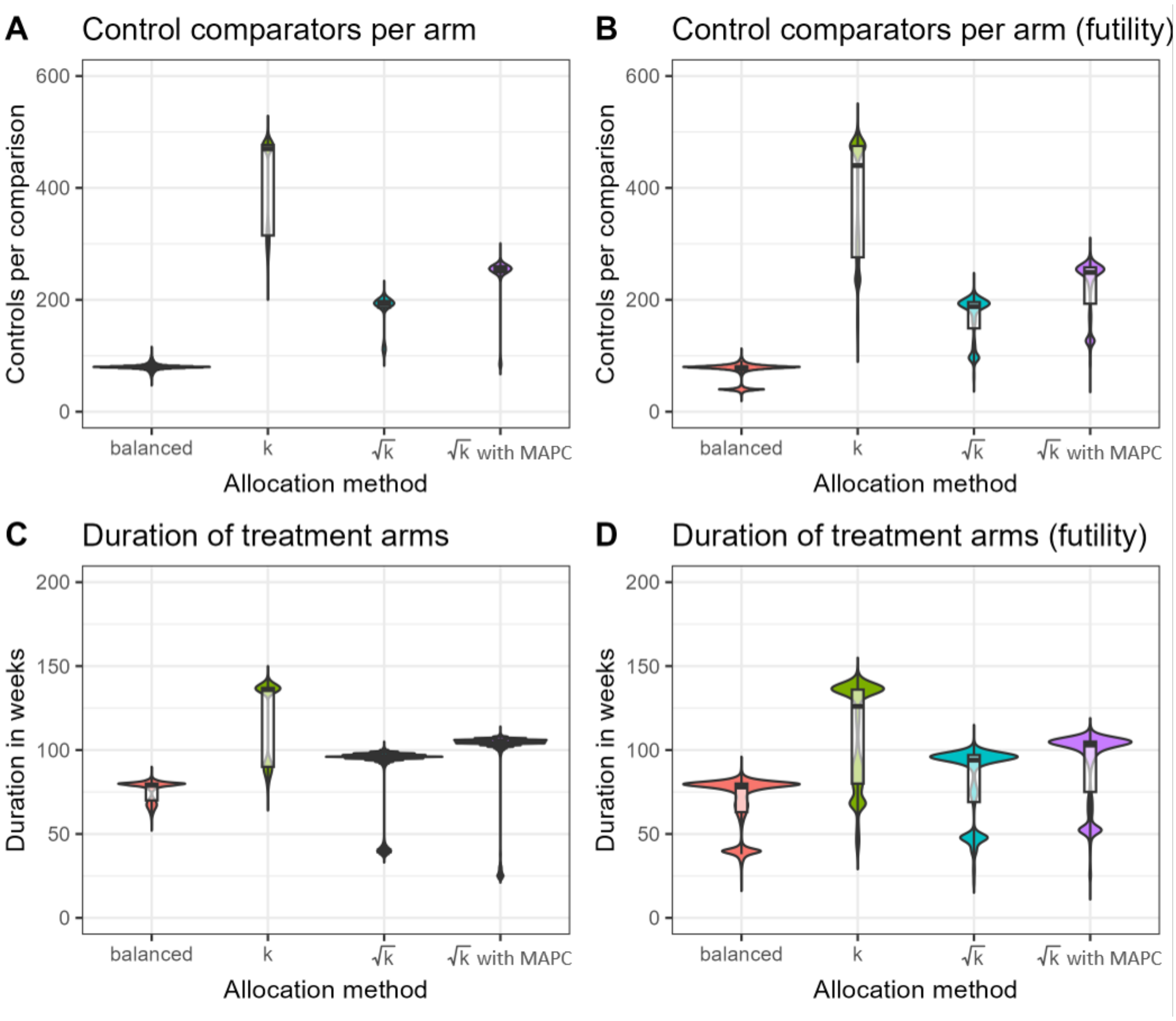

Figure 16: Duration of experimental treatment arms and number of control comparators per experimental treatment arm for different allocation methods. In the depicted scenario, all effect sizes are assumed to be equally likely and a maximal workload is assumed. (A) and (C) give the results without implementation of a futility analysis, and (B) and (D) for a futility boundary of 0.5 for the p-value. The values are depicted as violin plots with integrated box plots.

B) Pessimistic effect size distribution

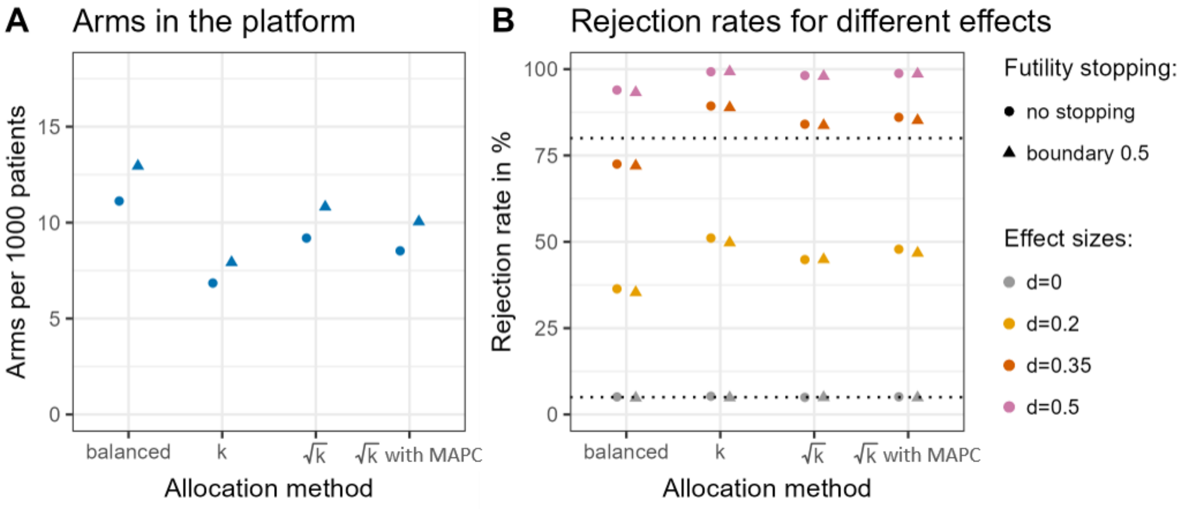

Figure 17: Standardised number of experimental treatment arms (without control arm) during the run-time of the platform trial and rejection rates for different allocation methods. In the depicted scenario, a pessimistic effect size distribution and a maximal workload are assumed. (A) gives the number of experimental treatment arms (without control arm) standardised for 1000 patients in the platform. In (B), the percentage of rejected null hypotheses stratified by treatment effect is depicted. It equals the type I error rate for $d = 0$ and the power for the other values of d. The dotted lines mark the 5% and the 80% levels.

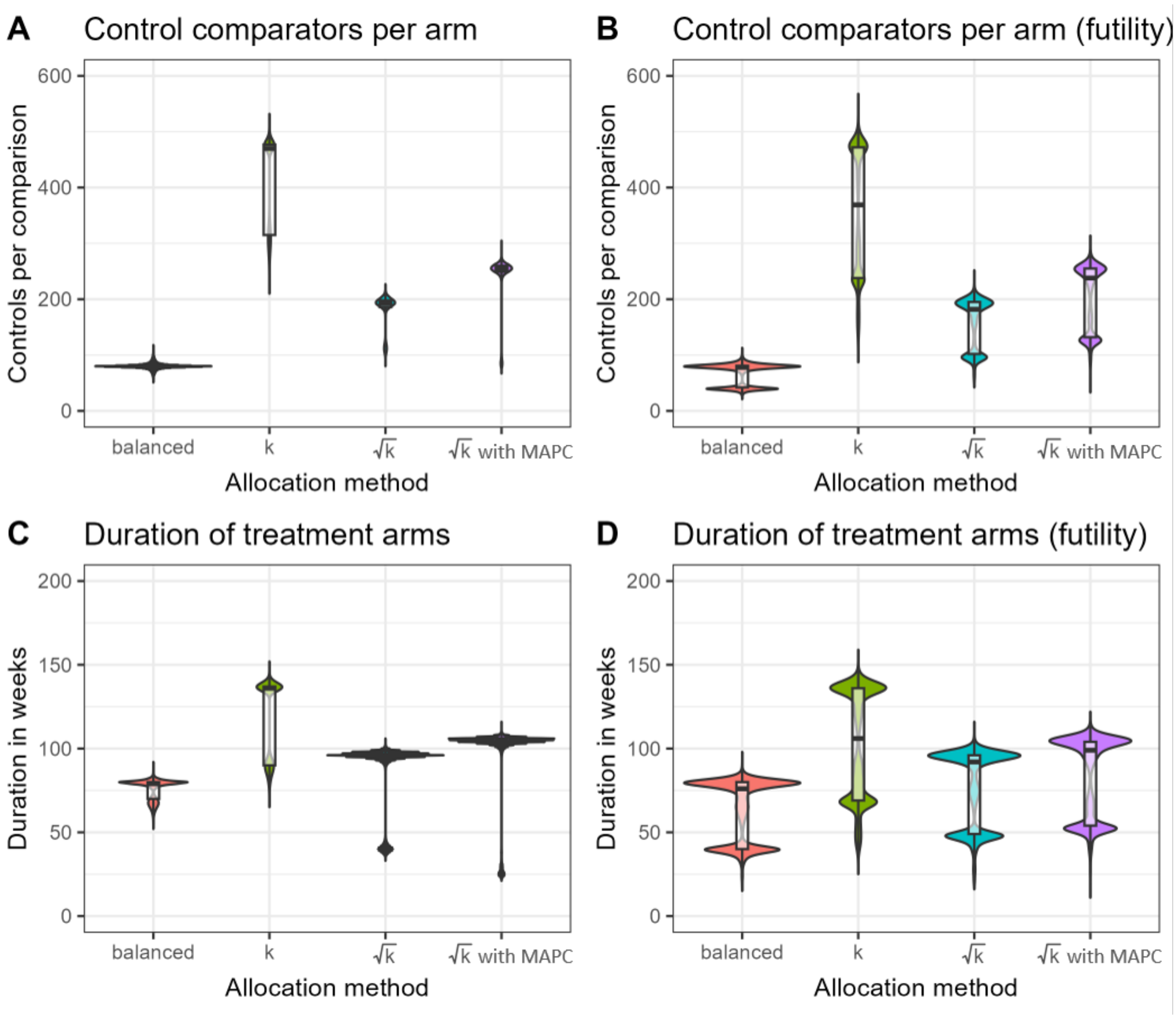

Figure 18: Duration of experimental treatment arms and number of control comparators per experimental treatment arm for different allocation methods. In the depicted scenario, a pessimistic effect size distribution and a maximal workload are assumed. (A) and (C) give the results without implementation of a futility analysis, and (B) and (D) for a futility boundary of 0.5 for the p-value. The values are depicted as violin plots with integrated box plots.

3.2 Additional results for the selection of the futility stopping rule

3.2.1 Results for the expected workload scenario

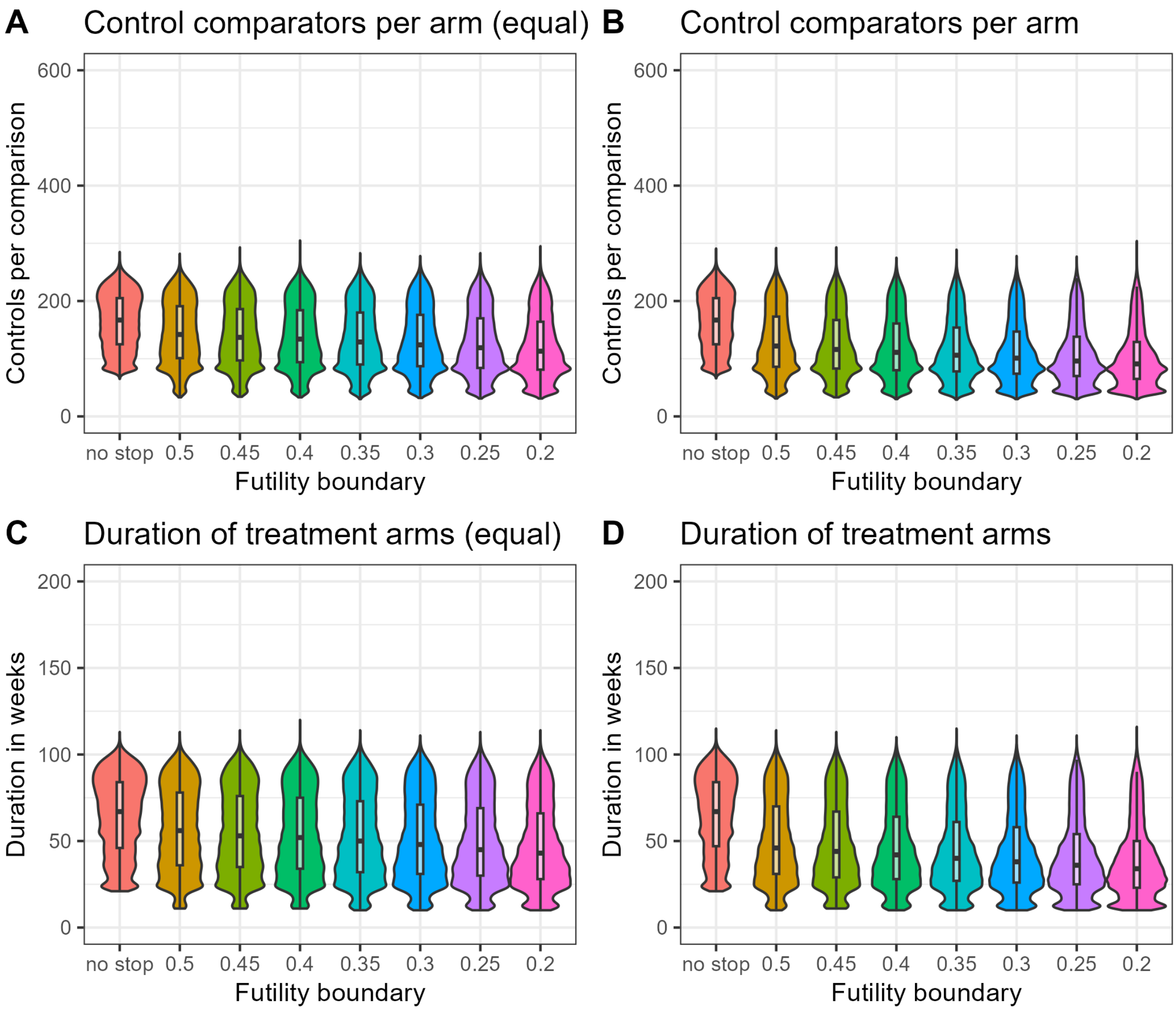

Figure 19: Duration of experimental treatment arms and number of control comparators per experimental treatment arm for different futility boundaries. In the depicted scenario, an expected workload is assumed. (A) and (C) give the results for an equal effect size distribution, and (B) and (D) for a pessimistic effect size distribution. The values are depicted as violin plots with integrated box plots.

Table 7: Mean number of concurrently running experimental treatment arms (without control arm) in platform trials running at expected capacity with an equal effect size distribution for different futility boundaries. The first line gives the values if the effects are distributed equally, and the second line gives the values if they are distributed pessimistically.

	Futility rule							
Effect distribution	no stop	0.5	0.45	0.4	0.35	0.3	0.25	0.2
equal	3.25	3.09	3.03	3.01	2.97	2.93	2. 88	2.81
pessimistic	3.25	2.90	2.84	2.79	2.74	2.68	2.62	2.54

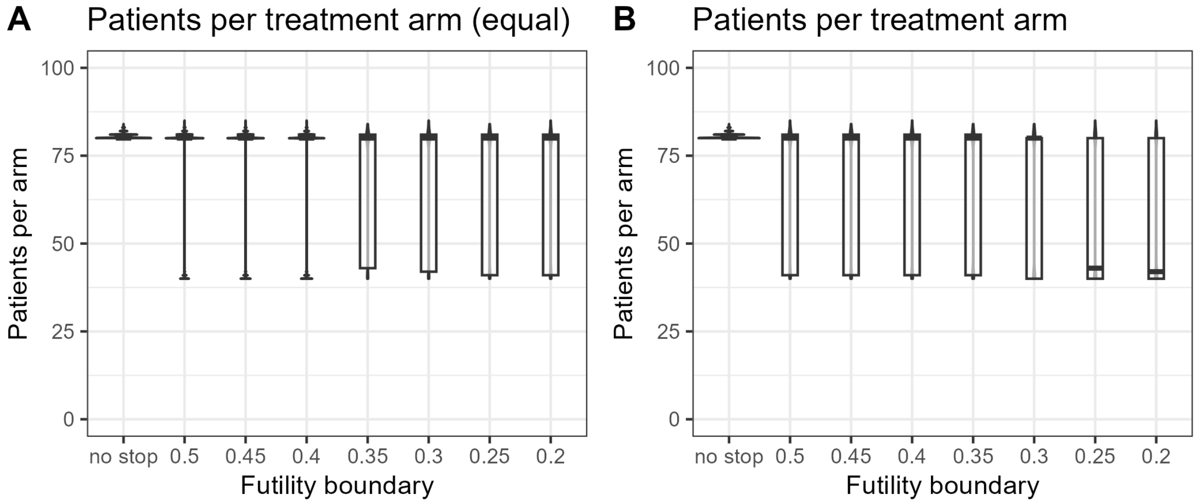

Figure 20: Number of patients per experimental treatment arm for different futility boundaries. In the depicted scenario, an expected workload is assumed. (A) gives the results for an equal effect size distribution and (B) for a pessimistic effect size distribution. The values are depicted as violin plots with integrated box plots.

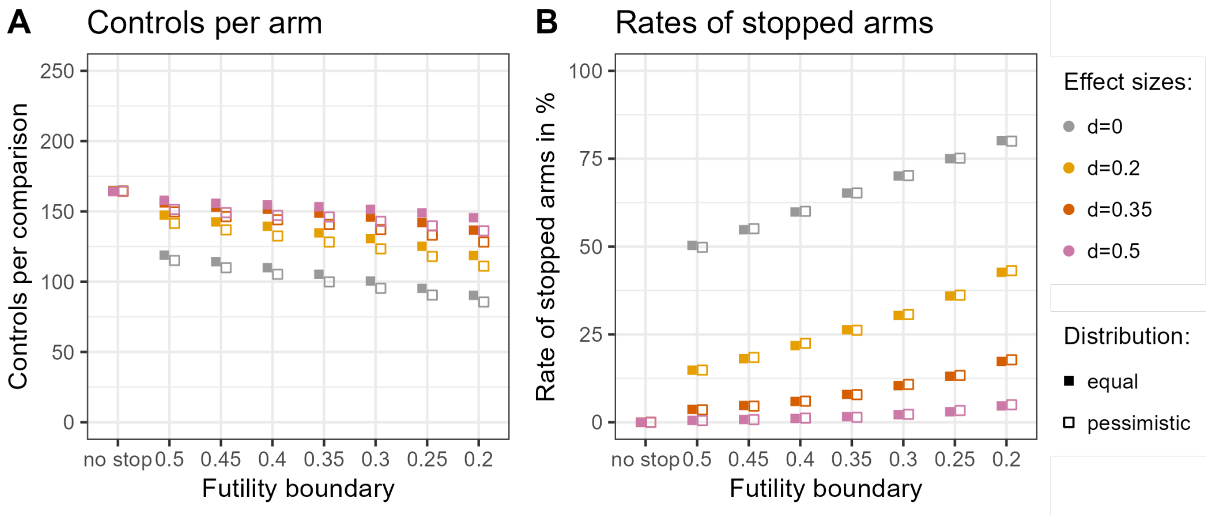

Figure 21: Mean number of patients in the individual control groups and rates of treatment arms stopped for futility for different futility boundaries. An expected workload is assumed. Both, the results of all effect sizes being equally likely and the scenario with a pessimistic effect size distribution are depicted. (A) gives the number of control comparators per treatment arm stratified by treatment effect. In (B), the percentage of treatment arms stopped for futility stratified by treatment effect is depicted.

3.2.2 Results for the maximal workload scenario

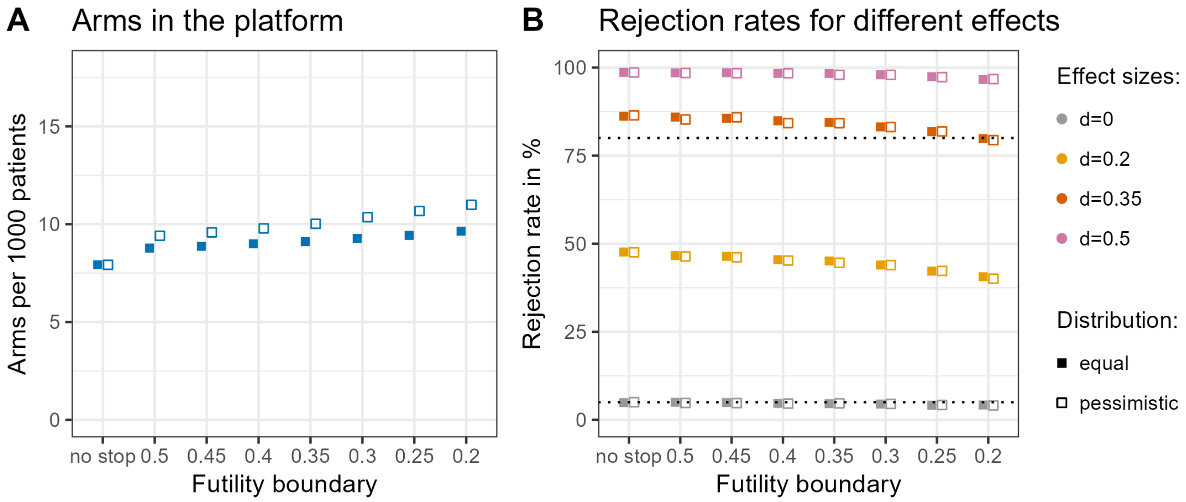

Figure 22: Standardised number of experimental treatment arms (without control arm) during the runtime of the platform trial and rejection rates for different futility boundaries. A maximal workload is assumed. Both, the scenario of all effect sizes being equally likely and the scenario with a pessimistic effect size distribution are depicted. (A) gives the number of experimental treatment arms (without control arm) standardised for 1000 patients in the platform. In (B), the percentage of rejected null hypotheses stratified by treatment effect is depicted. It equals the type I error rate for $d = 0$ and the power for the other values of d. The dotted lines mark the 5% and the 80% levels.

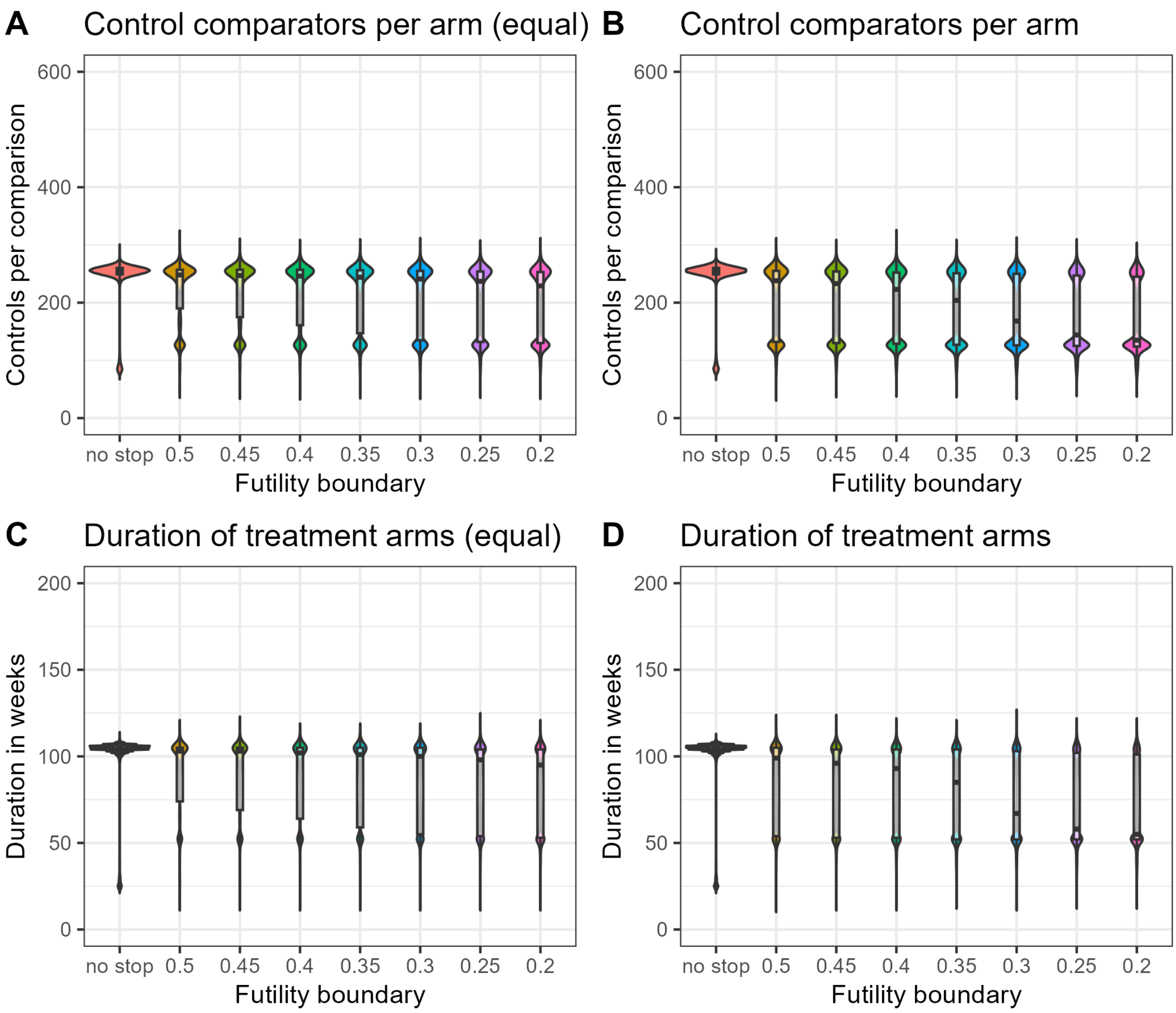

Figure 23: Duration of experimental treatment arms and number of control comparators per experimental treatment arm for different futility boundaries. In the depicted scenario, a maximal workload is assumed. (A) and (C) give the results for an equal effect size distribution, and (B) and (D) for a pessimistic effect size distribution. The values are depicted as violin plots with integrated box plots.

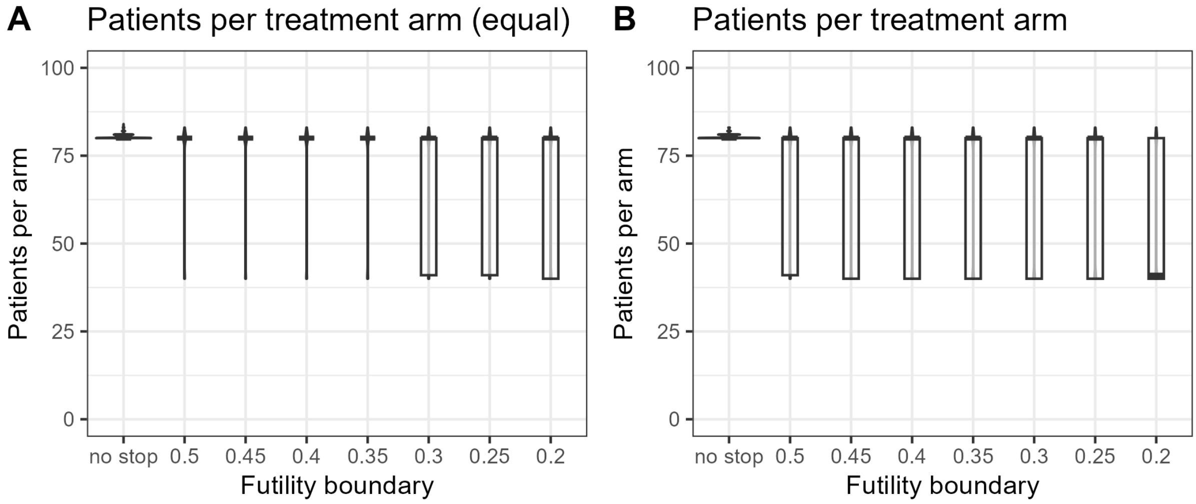

Figure 24: Number of patients per experimental treatment arm for different futility boundaries. In the depicted scenario, a maximal workload is assumed. (A) gives the results for an equal effect size distribution and (B) for a pessimistic effect size distribution. The values are depicted as violin plots with integrated box plots.

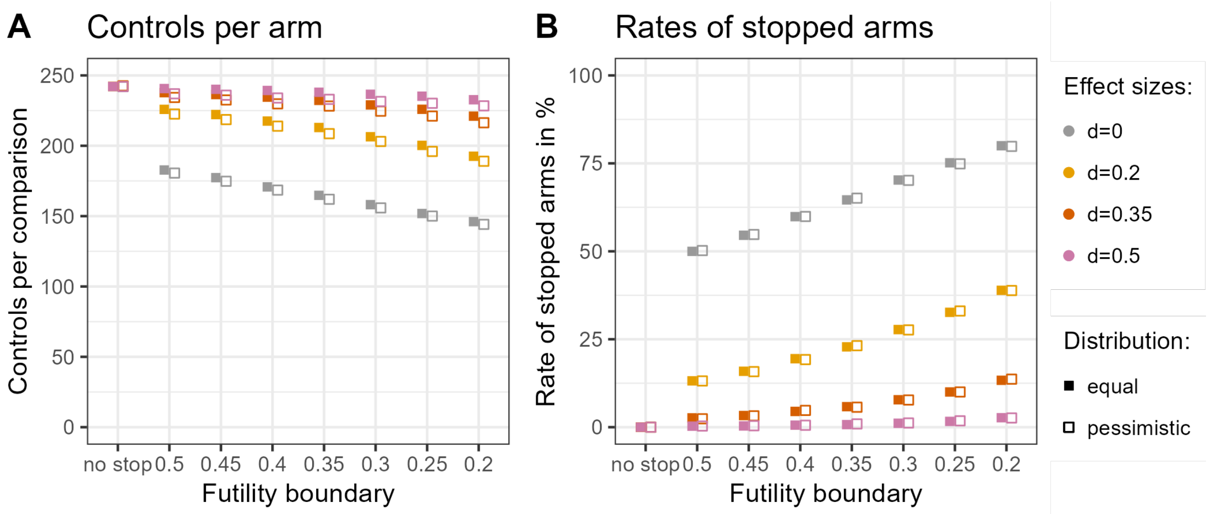

Figure 25: Mean number of patients in the individual control groups and rates of treatment arms stopped for futility for different futility boundaries. A maximal workload is assumed. Both, the results of all effect sizes being equally likely and the scenario with a pessimistic effect size distribution are depicted. (A) gives the number of control comparators per treatment arm stratified by treatment effect. In (B), the percentage of treatment arms stopped for futility stratified by treatment effect is depicted.

3.3 Additional results for the selection of the per arm sample size

3.3.1 Results for the expected workload scenario

A) Equal effect size distribution

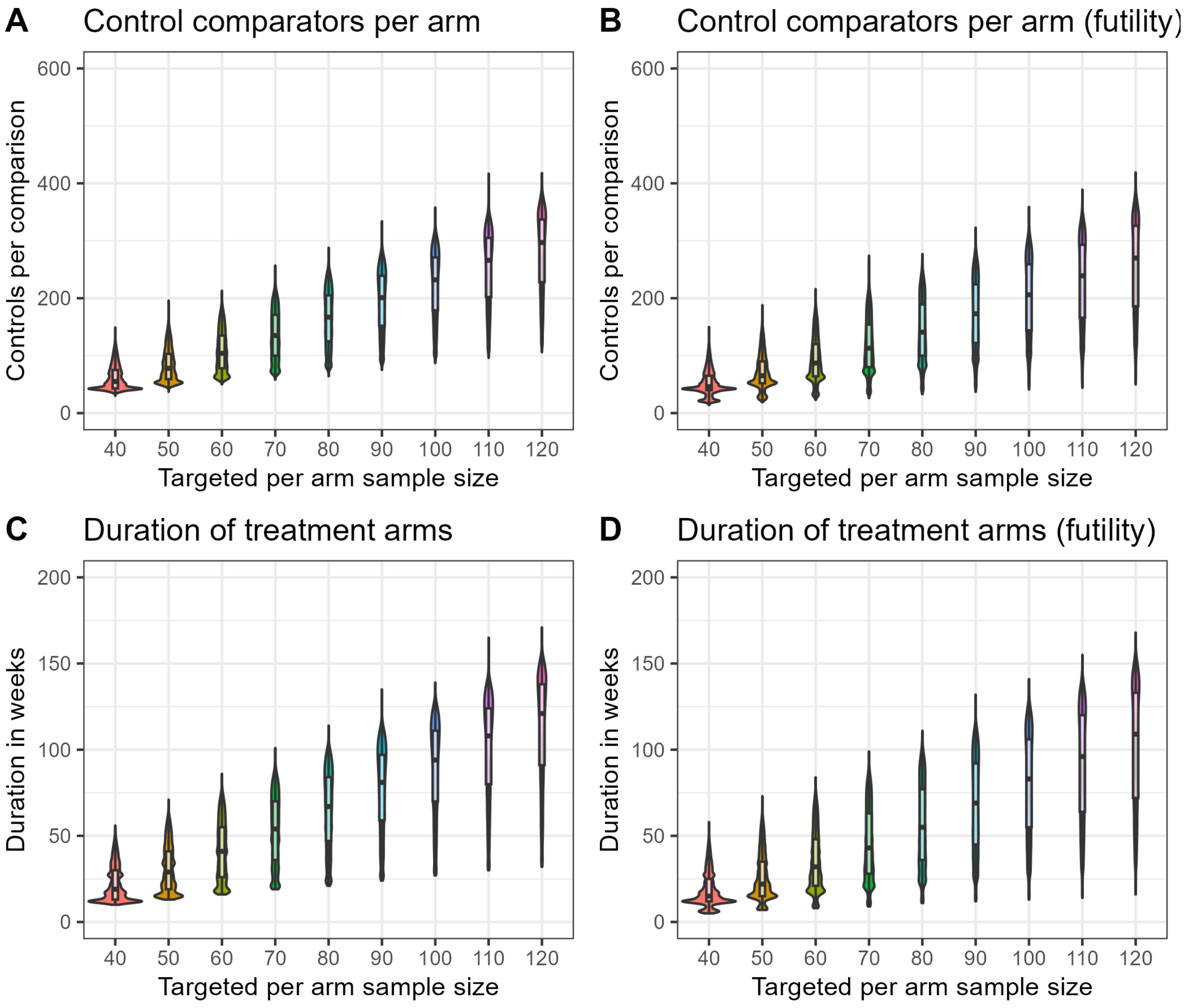

Figure 26: Duration of experimental treatment arms and number of control comparators per experimental treatment arm for different targeted sample sizes per treatment arm. In the depicted scenario, all effect sizes are assumed to be equally likely and an expected workload is assumed. (A) and (C) give the results without implementation of a futility analysis, and (B) and (D) for a futility boundary of 0.5 for the p-value. The values are depicted as violin plots with integrated box plots.

Table 8: Mean number of concurrently running experimental treatment arms (without control arm) in platform trials running at expected capacity with an equal effect size distribution for different targeted sample sizes per treatment arm. The first line gives the values if no futility stopping is implemented, and the second line gives the ones for a futility boundary of 0.5 for the p-value.

	Targeted per arm sample size								
Futility rule	40	50	60	70	80	90	100	110	120
no stop	1.97	2.28	2.62	2.97	3.26	3.52	3.71	3.85	3.95
0.5	1.83	2.12	2.42	2.76	3.06	3.35	3.58	3.77	3.90

B) Pessimistic effect size distribution

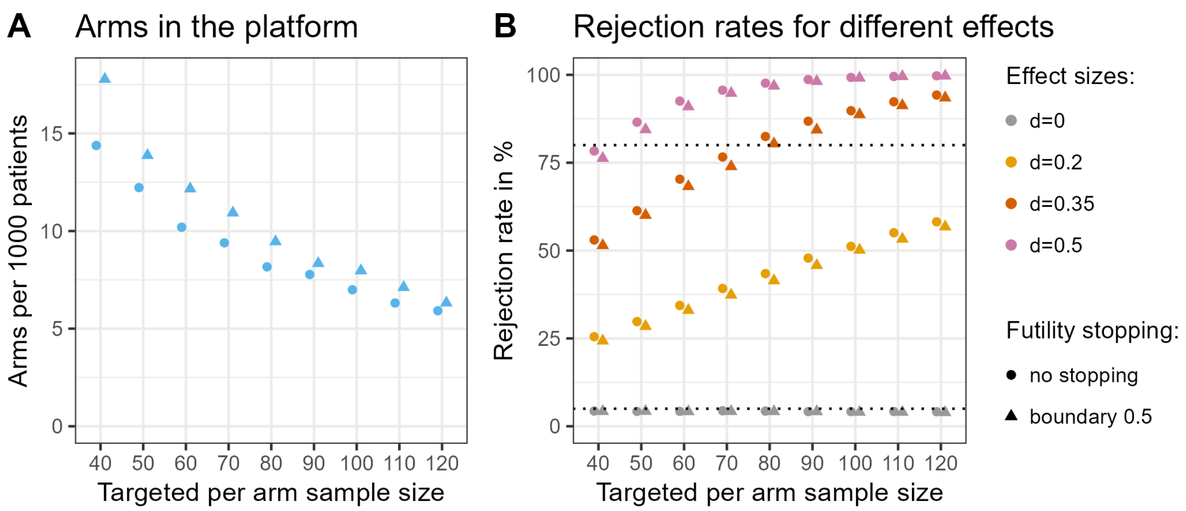

Figure 27: Standardised number of experimental treatment arms (without control arm) during the run-time of the platform trial and rejection rates for different targeted sample sizes per treatment arm. In the depicted scenario, a pessimistic effect size distribution and an expected workload are assumed. (A) gives the number of experimental treatment arms (without control arm) standardised for 1000 patients in the platform. In (B), the percentage of rejected null hypotheses stratified by treatment effect is depicted. It equals the type I error rate for $d = 0$ and the power for the other values of d. The dotted lines mark the 5% and the 80% levels.

Table 9: Mean number of concurrently running experimental treatment arms (without control arm) in platform trials running at expected capacity with a pessimistic effect size distribution for different targeted sample sizes per treatment arm. The first line gives the values if no futility stopping is implemented, and the second line gives the ones for a futility boundary of 0.5 for the p-value.

	Targeted per arm sample size								
Futility rule	40	50	60	70	80	90	100	110	120
no stop	1.96	2.28	2.62	2.95	3.27	3.52	3.70	3.85	3.96
0.5	1.76	2.00	2.28	2.60	2.91	3.19	3.44	3.65	3.81

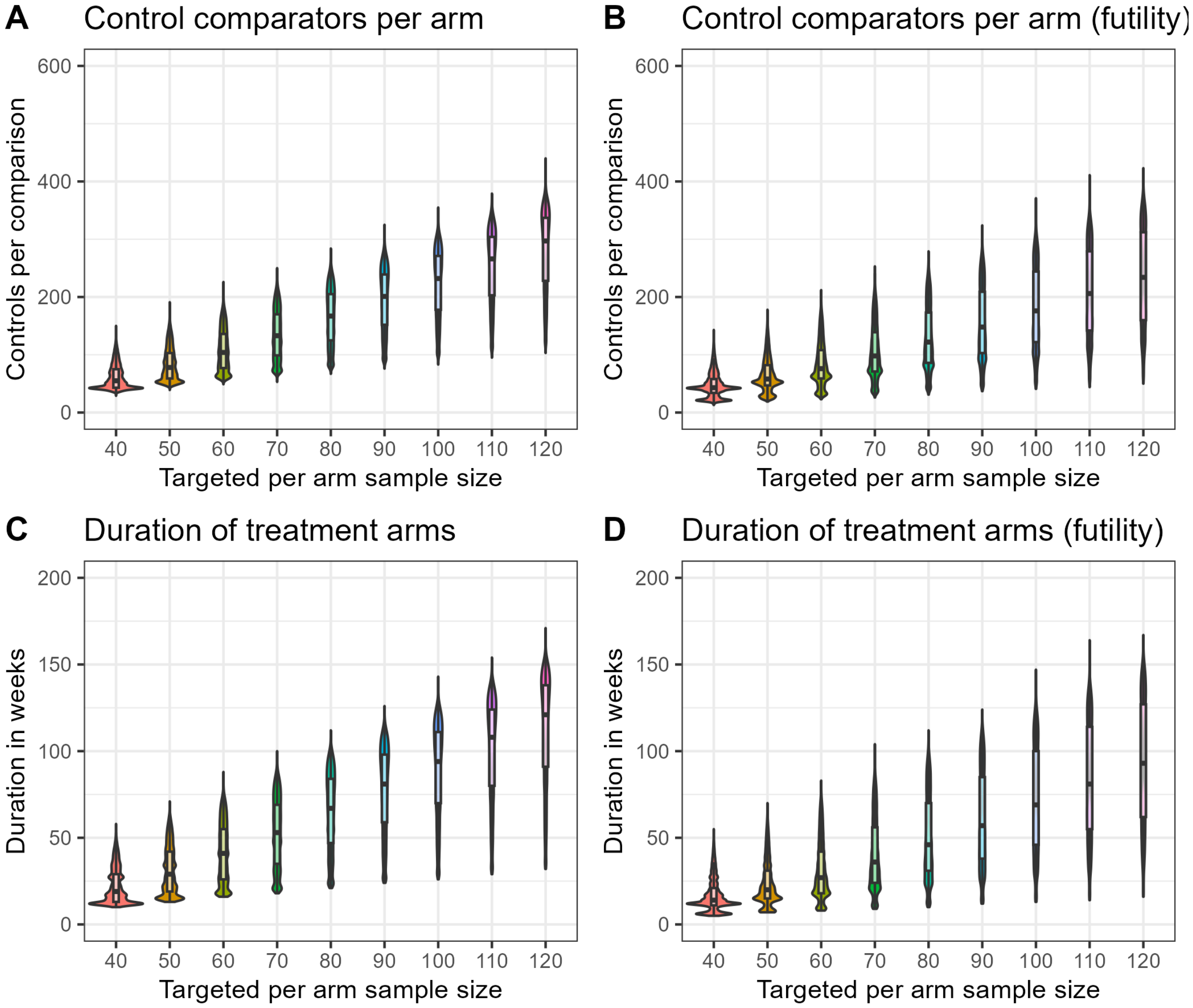

Figure 28: Duration of experimental treatment arms and number of control comparators per experimental treatment arm for different targeted sample sizes per treatment arm. In the depicted scenario, a pessimistic effect size distribution and an expected workload are assumed. (A) and (C) give the results without implementation of a futility analysis, and (B) and (D) for a futility boundary of 0.5 for the p-value. The values are depicted as violin plots with integrated box plots.

3.3.2 Results for the maximal workload scenario

A) Equal effect size distribution

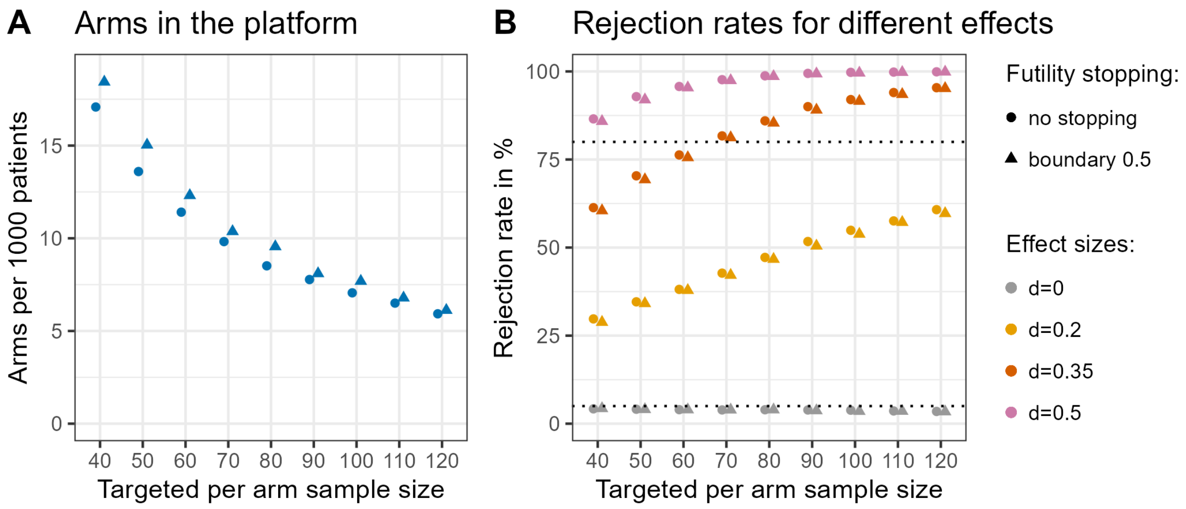

Figure 29: Standardised number of experimental treatment arms (without control arm) during the runtime of the platform trial and rejection rates for different targeted sample sizes per treatment arm. In the depicted scenario, all effect sizes are assumed to be equally likely and a maximal workload is assumed. (A) gives the number of experimental treatment arms (without control arm) standardised for 1000 patients in the platform. In (B), the percentage of rejected null hypotheses stratified by treatment effect is depicted. It equals the type I error rate for $d = 0$ and the power for the other values of d. The dotted lines mark the 5% and the 80% levels.

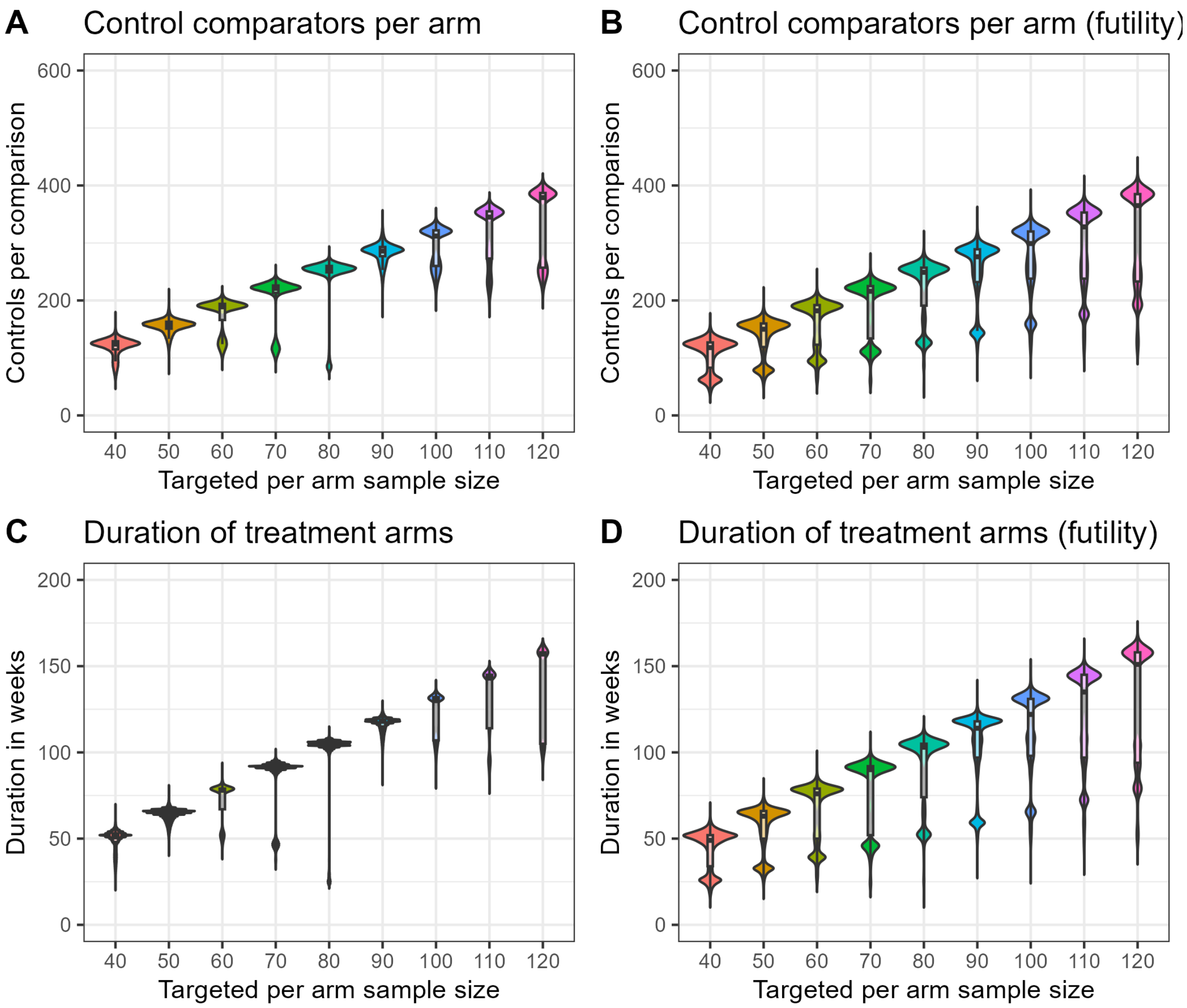

Figure 30: Duration of experimental treatment arms and number of control comparators per experimental treatment arm for different targeted sample sizes per treatment arm. In the depicted scenario, all effect sizes are assumed to be equally likely and a maximal workload is assumed. (A) and (C) give the results without implementation of a futility analysis, and (B) and (D) for a futility boundary of 0.5 for the p-value. The values are depicted as violin plots with integrated box plots.

B) Pessimistic effect size distribution

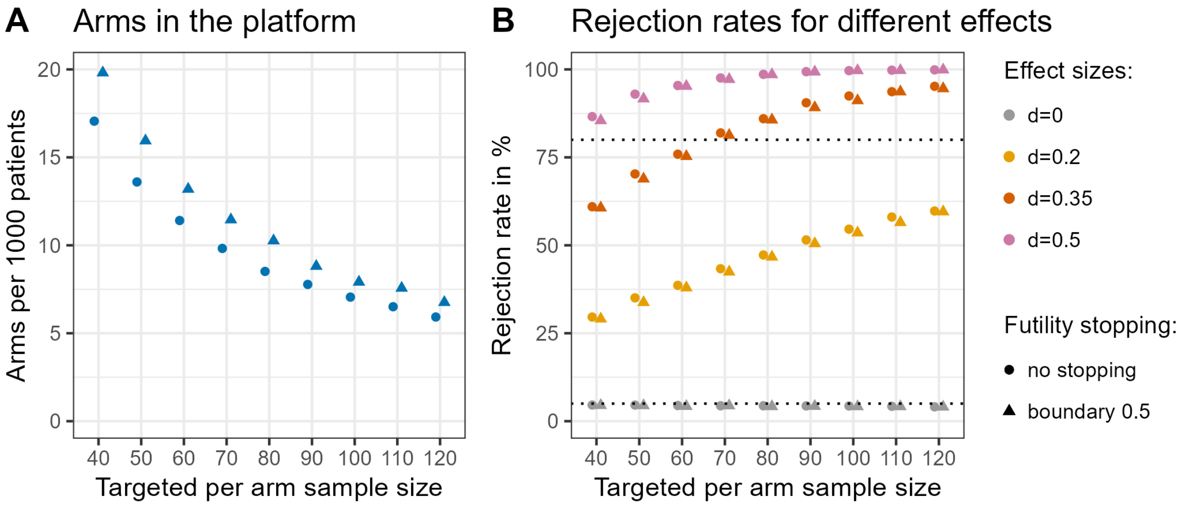

Figure 31: Standardised number of experimental treatment arms (without control arm) during the runtime of the platform trial and rejection rates for different targeted sample sizes per treatment arm. In the depicted scenario, a pessimistic effect size distribution and a maximal workload are assumed. (A) gives the number of experimental treatment arms (without control arm) standardised for 1000 patients in the platform. In (B), the percentage of rejected null hypotheses stratified by treatment effect is depicted. It equals the type I error rate for $d = 0$ and the power for the other values of d. The dotted lines mark the 5% and the 80% levels.

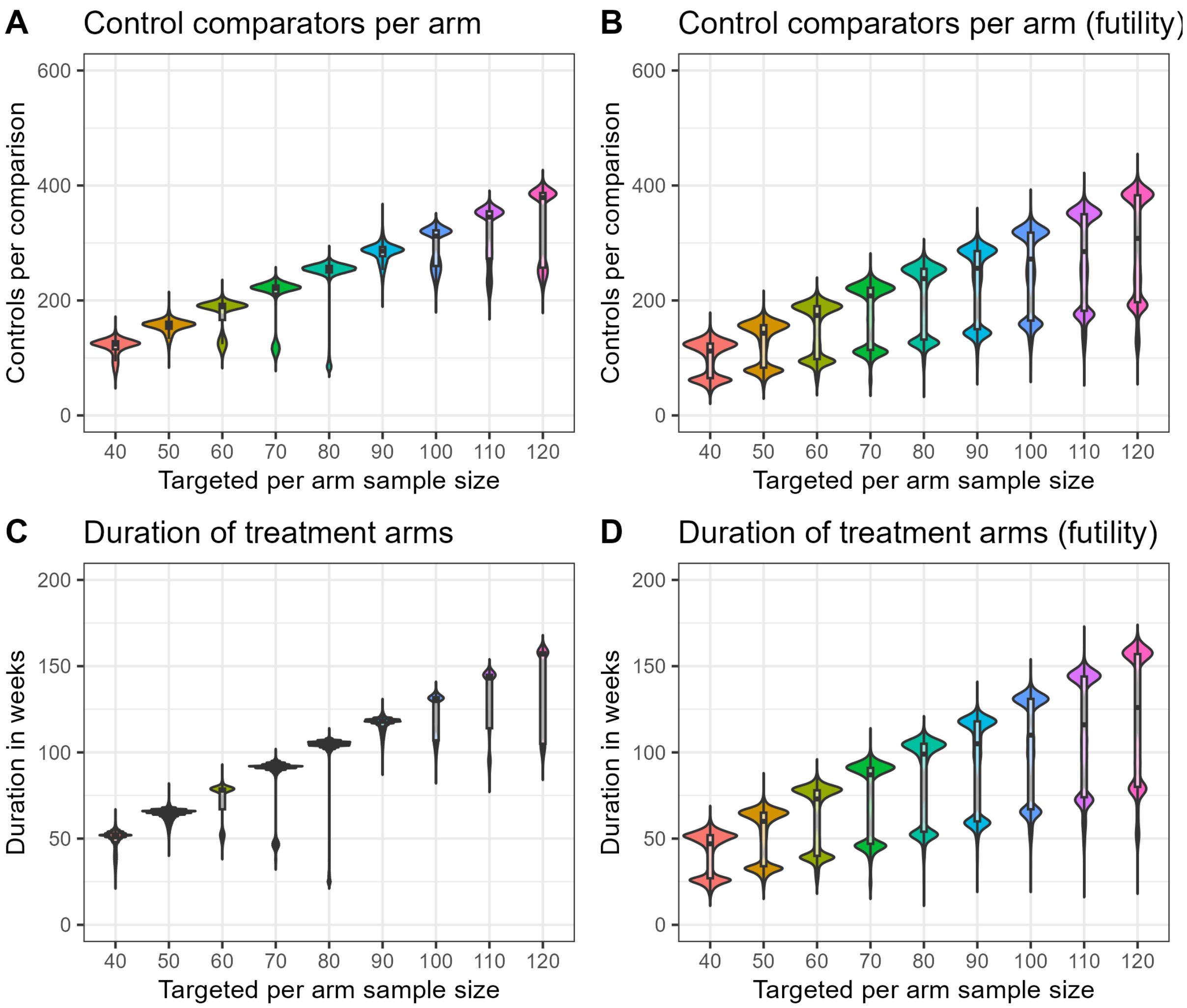

Figure 32: Duration of experimental treatment arms and number of control comparators per experimental treatment arm for different targeted sample sizes per treatment arm. In the depicted scenario, a pessimistic effect size distribution and a maximal workload are assumed. (A) and (C) give the results without implementation of a futility analysis, and (B) and (D) for a futility boundary of 0.5 for the p-value. The values are depicted as violin plots with integrated box plots.

4 Additional results for the comparison of trial types

A) Equal effect size distribution

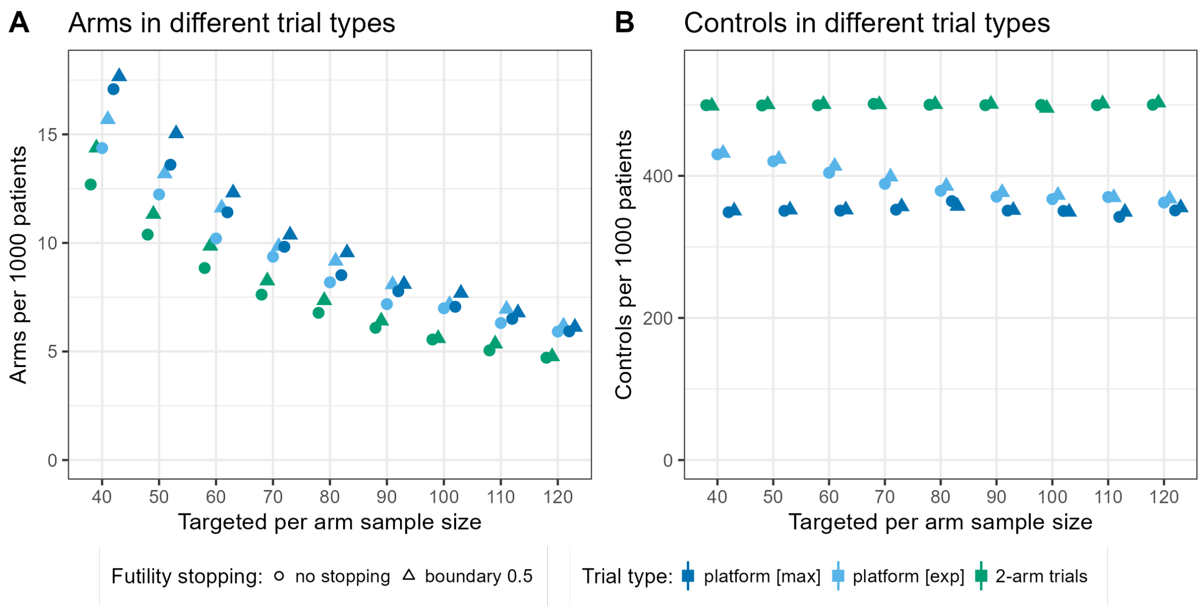

Figure 33: Comparison of operating characteristics in different trial types. All effect sizes are assumed to be equally likely. The circles give the values without the implementation of an interim analysis and the triangles give the corresponding values when a futility boundary of 0.5 for the p-value is applied. The sample size depicted on the x-axis was examined in steps of 10. The small variation in the x-direction is based on jittering for better readability. (A) shows the mean number of arms per 1000 patients for the three different trial types: platform trial with maximal capacity utilisation, platform trial with expected load in the MDD case, and the traditional approach with a series of individual two-arm randomised controlled trials. (B) gives the number of controls per 1000 patients for the same types of trials. The dark blue marks are at about 350 since the MAPC is set to 35%. For higher sample sizes, more experimental arms are concurrently in the platform trial with an expected workload, so the light blue marks converge to the dark blue ones.

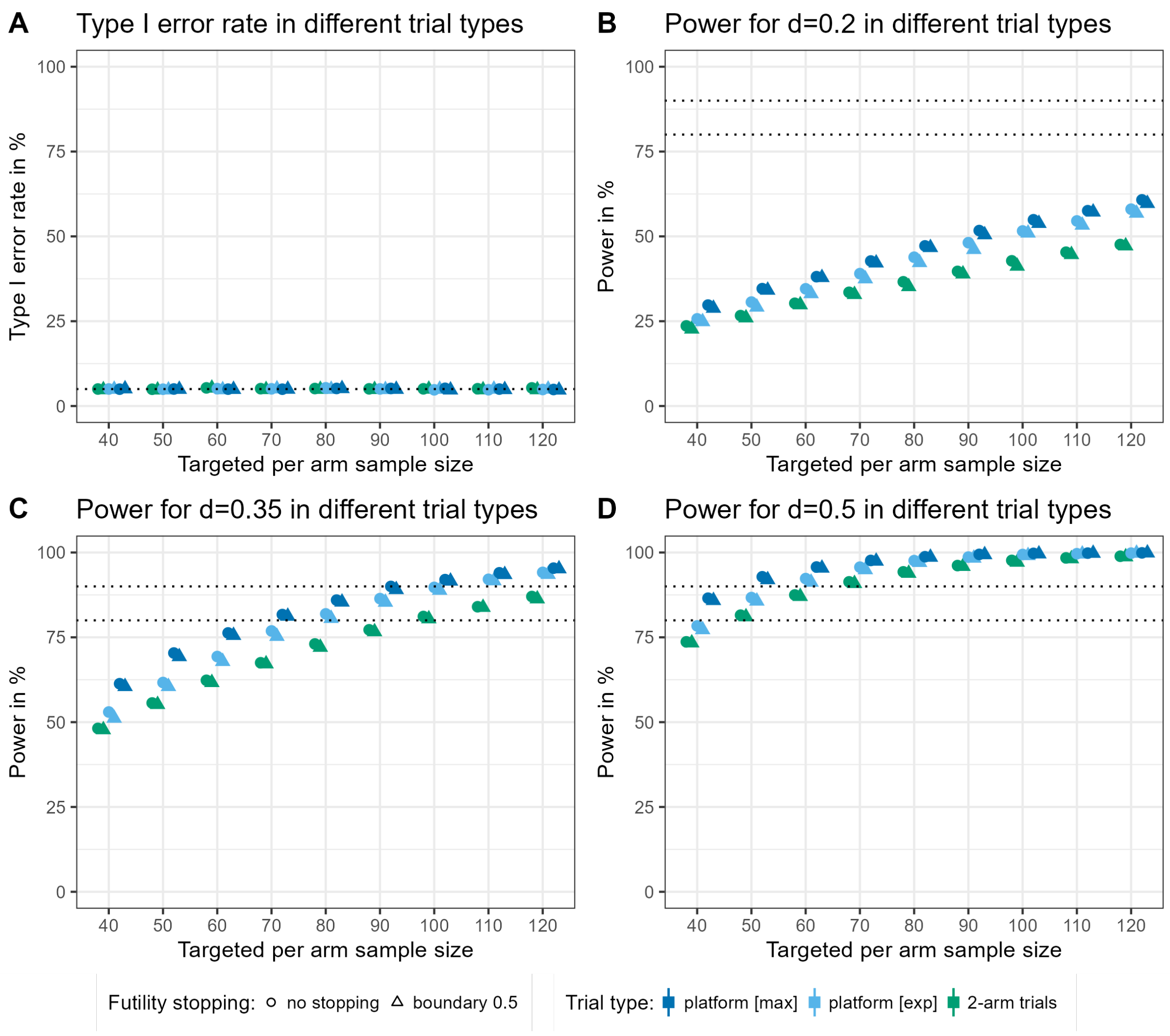

Figure 34: Comparison of operating characteristics in different trial types. All effect sizes are assumed to be equally likely. The circles give the values without the implementation of an interim analysis and the triangles give the corresponding values when a futility boundary of 0.5 for the p-value is applied. The sample size depicted on the x-axis was examined in steps of 10. The small variation in the x-direction is based on jittering for better readability. The rejection rates are depicted stratified by the four different investigated effect sizes d. The rejection rate equals the type I error rate for $d = 0$ and the power for the other values of d. The dotted line in (A) highlights the significance level of 5%. The dotted lines in (B), (C) and (D) indicate the 80% and 90% marks.

B) Pessimistic effect size distribution

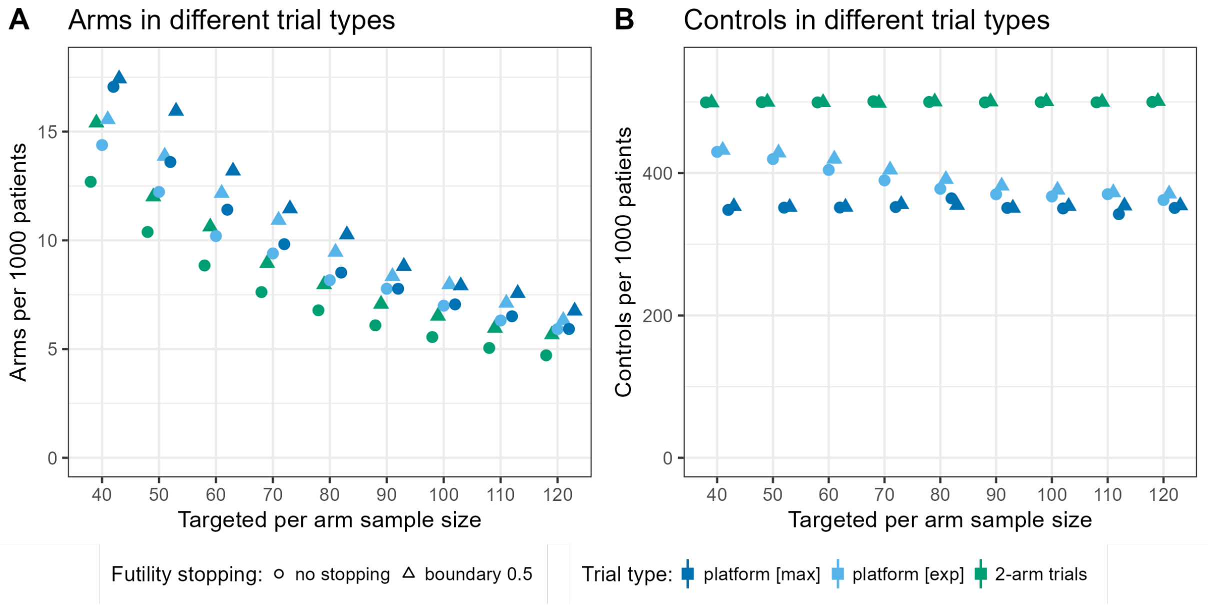

Figure 35: Comparison of operating characteristics in different trial types. The effect sizes are assumed to be pessimistically distributed. The circles give the values without the implementation of an interim analysis and the triangles give the corresponding values when a futility boundary of 0.5 for the p-value is applied. The sample size depicted on the x-axis was examined in steps of 10. The small variation in the x-direction is based on jittering for better readability. (A) shows the mean number of arms per 1000 patients for the three different trial types: platform trial with maximal capacity utilisation, platform trial with expected load in the MDD case, and the traditional approach with a series of individual two-arm randomised controlled trials. (B) gives the number of controls per 1000 patients for the same types of trials. The dark blue marks are at about 350 since the MAPC is set to 35%. For higher sample sizes, more experimental arms are concurrently in the platform trial with an expected workload, so the light blue marks converge to the dark blue ones.

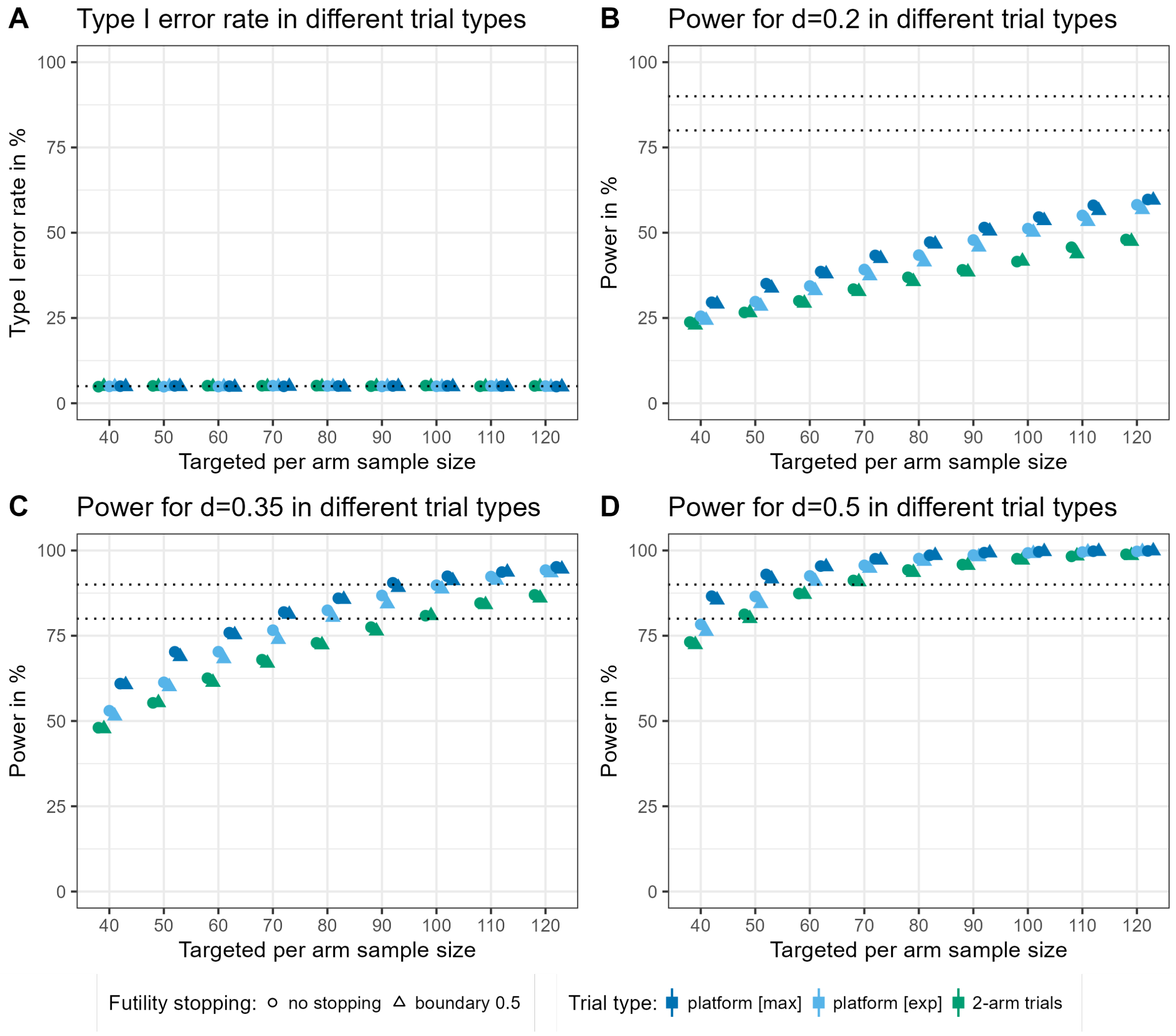

Figure 36: Comparison of operating characteristics in different trial types. The effect sizes are assumed to be pessimistically distributed. The circles give the values without the implementation of an interim analysis and the triangles give the corresponding values when a futility boundary of 0.5 for the p-value is applied. The sample size depicted on the x-axis was examined in steps of 10. The small variation in the x-direction is based on jittering for better readability. The rejection rates are depicted stratified by the four different investigated effect sizes d. The rejection rate equals the type I error rate for $d = 0$ and the power for the other values of d. The dotted line in (A) highlights the significance level of 5%. The dotted lines in (B), (C) and (D) indicate the 80% and 90% marks.

5 Comparison of platform end criteria

Platform trials can potentially run perpetually if no end criterion is specified. For the platform trial in MDD, the idea is to enable new treatment arms to enter the platform up to month 60. After this time point, all arms still in the trial continue enrolling patients until a decision is made. This rule leads to quite varying overall sample sizes in the platform trial if different design options are applied, like different allocation methods, see Figure 37 (B). In order to facilitate better comparability between different design options, an additional condition was introduced in the simulation study that must be fulfilled for new treatment arms to enter the platform trial. They can only enter if it is expected that at least 20% of the desired per arm sample size can be accrued until month 60. This rule leads to a much more homogeneous overall sample size of the platform trial. Therefore, we applied the 20% rule in all simulations. Figure 37 shows the results exemplary for the different allocation methods.

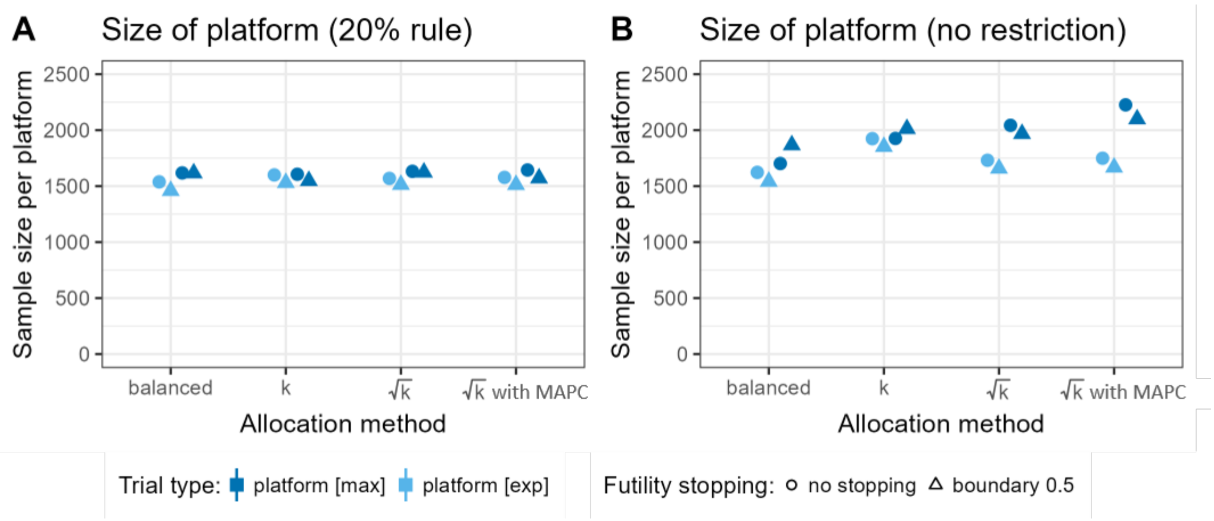

Figure 37: Comparison of different end criteria for platform trial at expected workload and maximal workload. The circles give the values without the implementation of an interim analysis and the triangles give the corresponding values when a futility boundary of 0.5 for the p-value is applied. (A) shows the overall sample size of the platform trial for different allocation methods if treatment arms can enter the platform only if at least 20% of the desired per-arm sample size can be accrued up to month 60. (B) shows the corresponding values if treatment arms can enter the platform up to month 60 with no additional rule applied. The per-arm sample size was set to 80 and an equal effect size distribution was assumed.

Abbreviations

Table 10: Table of all the abbreviations that are used throughout the manuscript.

Abbreviation	Description
ANCOVA	analysis of covariance
EMA	European Medicines Agency
EU-PEARL	EU Patient-Centric clinicAl tRial pLatforms
FDA	Food and Drug Administration
FDR	false discovery rate
FWER	family-wise error rate
IMI	Innovative Medicines Initiative
ITF	Innovation Task Force
MADRS	Montgomery-Åsberg Depression Rating Scale
MAPC	Minimum Allocation Probability to Control
MDD	Major Depressive Disorder
NASH	Non-Alcoholic Steatohepatitis
SNRI	serotonin norepinephrine reuptake inhibitors
SSRI	selective serotonin reuptake inhibitor
TRD	Treatment-resistant Depression

Acknowledgements

The authors are grateful to the EU-PEARL investigators who contributed to the development of the MDD master protocol. The EU-PEARL MDD investigators are: Jelena Brasanac, Woo Ri Chae, Michaela Maria Freitag, Stefan Gold, Eugenia Kulakova, Christian Otte, Dario Zocholl, Francesco Benedetti, Witte Hoogendijk, Marta Bofill-Roig, Franz König, Martin Posch, Yanina Flossbach, Tasneem Arsiwala, Alexandra Bobirca, Fernanda Baroso de Sousa, Pol Ibanez-Jimenez, Gabriela Perez-Fuentes, Toni Ramos-Quiroga, Melissa Kose, Giulia Lombardi, Carmine Pariante, Luca Sforzini, Courtney Worrell

Funding

EU-PEARL (EU Patient-cEntric clinicAl tRial pLatforms) project has received funding from the Innovative Medicines Initiative (IMI) Joint Undertaking (JU) under grant agreement No 853966. This Joint Undertaking receives support from the European Union's Horizon 2020 research and innovation programme and EFPIA and Children's Tumor Foundation, Global Alliance for TB Drug Development non-profit organisation, Springworks Therapeutics Inc. This publication reflects the authors' views. Neither IMI nor the European Union, EFPIA, or any Associated Partners are responsible for any use that may be made of the information contained herein.
MMF additionally received funding from the German Research Foundation (Project number RA 2347/11-1).
